# Design and synthesis of isoxazolidine core-based selective COX-2 inhibitors: *in vitro* inhibitor activity and *in silico* modeling

**DOI:** 10.1039/d6ra03616c

**Published:** 2026-08-10

**Authors:** Arwa Al-Adhreai, Mohammed ALSaeedy, Demokrat Nuha, Muhammed Trawally, Ramzan ullah, Syed Mansoor Ali, ZabnAllah M Alaizeri, Selahaddin Güner, Mansour K. Gatasheh

**Affiliations:** a Department of Chemistry “Giacomo Ciamician” University of Bologna 40126 Bologna BO Italy; b University for Business and Technology, Faculty of Medical Biochemistry Prishtina Kosovo; c Department of Pharmaceutical Chemistry, Faculty of Pharmacy, Istanbul University Istanbul Türkiye; d Shanghai Ultra-Precision Optical Manufacturing Engineering Center, Department of Optical Science and Engineering, Fudan University Shanghai 200433 China; e Department of Physics and Astronomy, College of Science, King Saud University Riyadh 11451 Saudi Arabia; f Kocaeli University, Faculty of Arts & Sciences, Department of Chemistry 41001 Umuttepe-Kocaeli Turkey mohammedali.alsaeedy@kocaeli.edu.tr; g Department of Biochemistry, College of Science, King Saud University P.O. Box 2455 Riyadh 11451 Saudi Arabia

## Abstract

A novel series of tricyclic isoxazolidine–imidazole derivatives (3a–l) bearing diverse substituted phenyl groups were rationally designed, synthesized, and evaluated as selective cyclooxygenase-2 (COX-2) inhibitors. The target compounds were efficiently obtained *via* regio- and diastereoselective 1,3-dipolar cycloaddition of C-aryl-3-methylphenyl nitrones with 1-vinylimidazole. Comprehensive spectroscopic characterization was performed using FTIR, ^1^H NMR, ^13^C NMR, and LC-MS spectrometry. *In vitro* assays demonstrated potent COX-2 inhibition with minimal COX-1 activity, achieving high selectivity. Compound 3a exhibited very potent COX-2 inhibition (pIC_50_ = 6.74 ± 0.05) but weak COX-1 inhibition (pIC_50_ = 4.19 ± 0.02), the highest selectivity index (SI = 361.1) in the series compared with the reference drug celecoxib (SI = 74.9), compounds 3e and 3j also exhibited higher selectivity than celecoxib. Molecular docking studies elucidated key binding interactions within the COX-2 active site, confirming favorable steric and electronic complementarity. *In silico* ADMET predictions also revealed the ideal drug-likeness, oral bioavailability, and pharmacokinetic characteristics. The combined synthetic, biological, and computational findings identify these derivatives as promising lead compounds for the development of selective COX-2 inhibitors with possible therapeutic value in the treatment of inflammatory diseases. Density functional theory (DFT) calculations were conducted to investigate the electronic structure and reactivity of compounds 3a, 3e, and 3j. The results indicated that compound 3a was the most reactive and electrophilic derivative. The study also revealed that molecular size and substituent distribution significantly influenced the stability, reactivity, and electronic properties of the investigated compounds.

## Abstract 2

A novel series of tricyclic isoxazolidine–imidazole derivatives (3a–l) bearing substituted phenyl groups was designed, synthesized, and evaluated as selective cyclooxygenase-2 (COX-2) inhibitors. The graphical abstract image was partly generated with Google Gemini.

## Graphical abstract

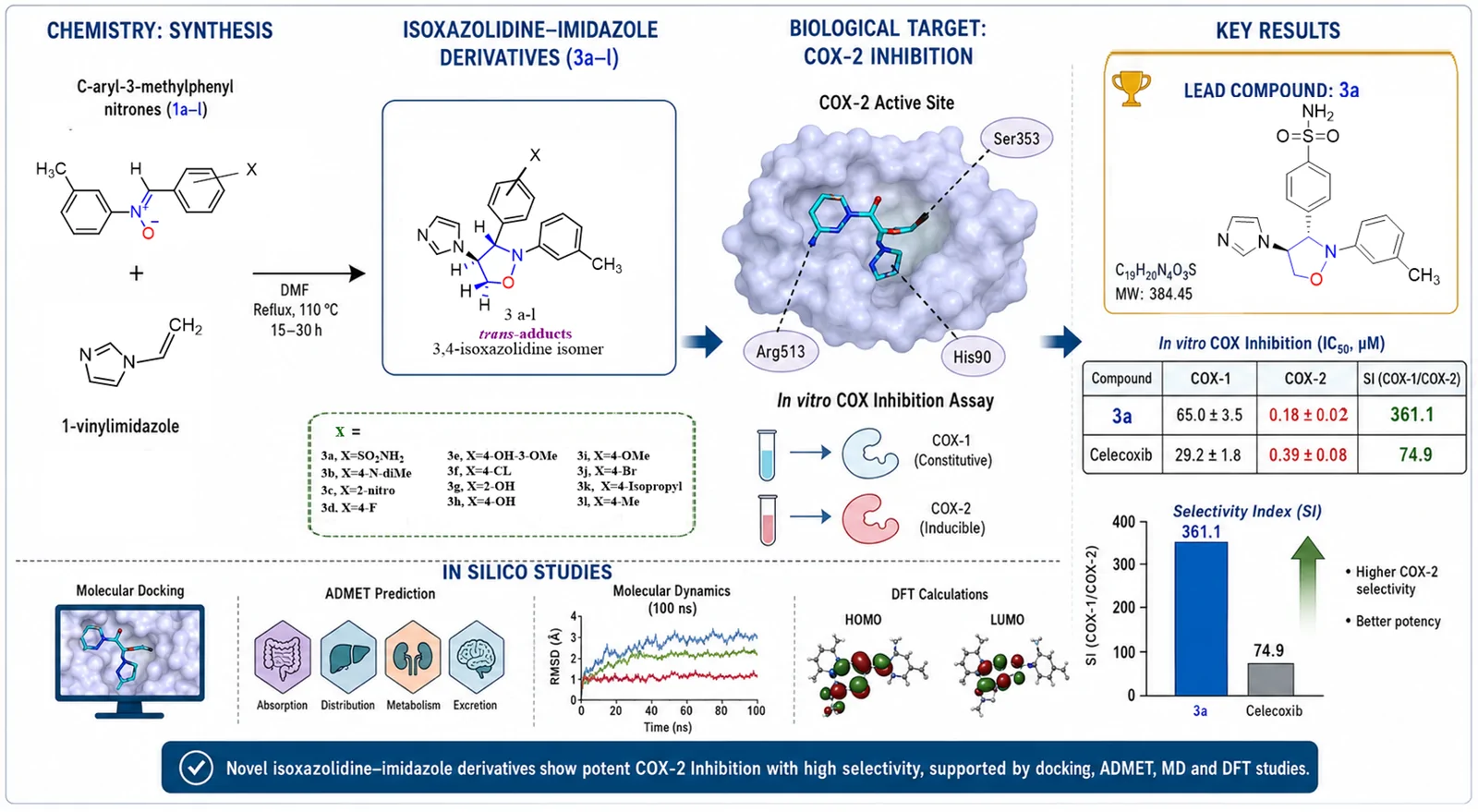

## 1. Introduction

**Fig. 1:**
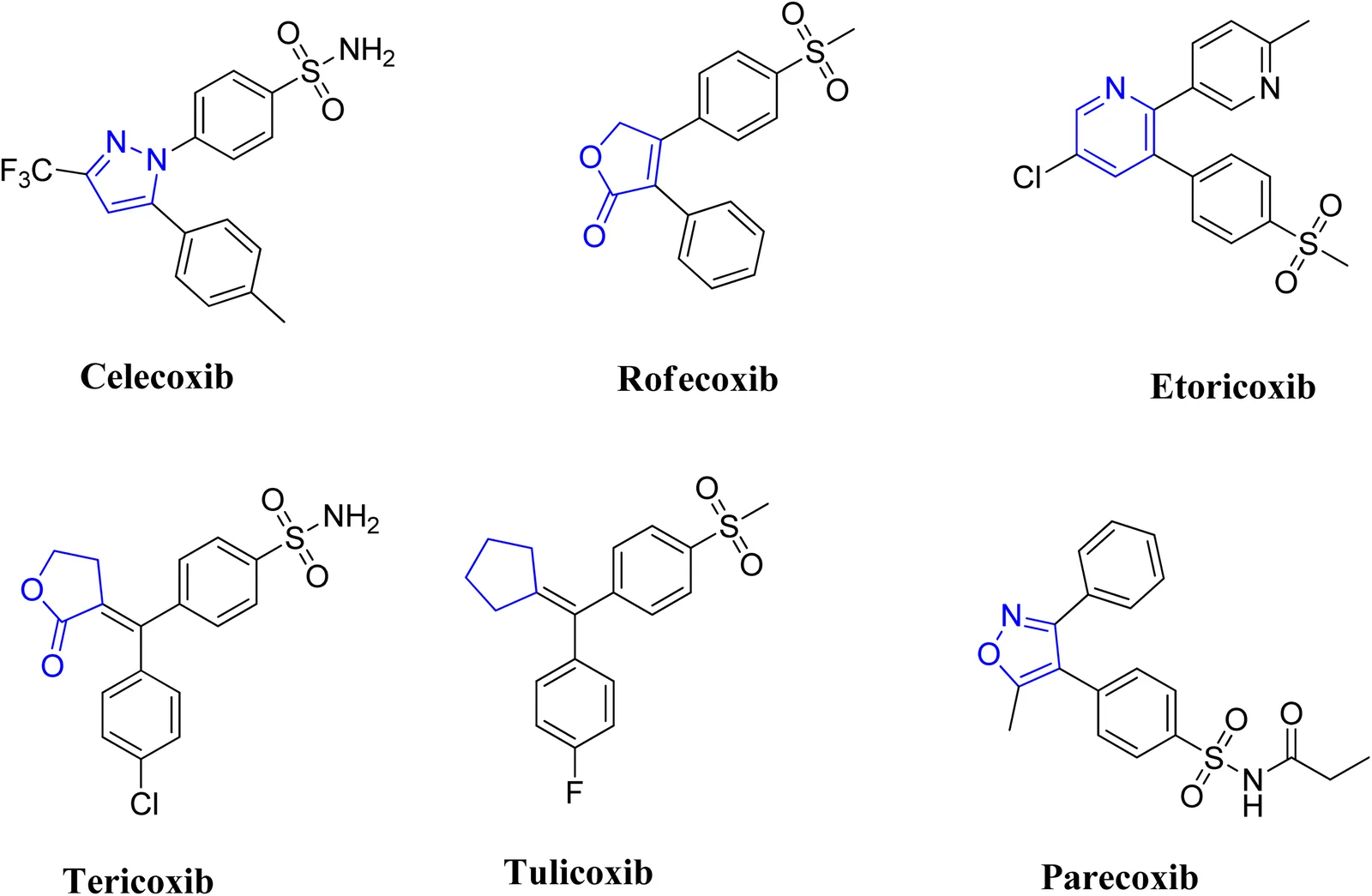
Representative examples of selective COX-2 inhibitors.

Inflammation is a natural and a basic defense mechanism that protects the human body against pathogenic attacks by viruses, bacteria, and fungi.^1,2^ It is a complex biological process involving the coordinated activation of immune cells, enzymes, and inflammatory mediators to eliminate harmful stimuli and promote tissue repair.^3^ The classical signs of inflammation, such as redness, swelling, pain, heat, loss of tissue or organ functions, are clinical characteristics of inflammation.^4^ Though acute inflammation has a crucial role in host defense, its continuance or deregulation is one of the factors in the pathogenesis of many chronic diseases including arthritis, cardiovascular diseases, neurodegenerative diseases and cancer.^5,6^ Cyclooxygenases (COXs) are key enzymes in the inflammatory pathway that catalyze the conversion of arachidonic acid to prostaglandins, which are major mediators of inflammation and pain ^7,8^. Two major cyclooxygenase (COX) isoforms have been identified: COX-1, a constitutively expressed enzyme responsible for maintaining physiological homeostasis, particularly in the gastrointestinal tract and platelets, and COX-2, an inducible isoform that is upregulated by inflammatory stimuli, cytokines, and growth factors.^9,10^ The chemical structures of representative selective COX-1 and COX-2 inhibitors are illustrated in Fig. 1, highlighting the structural evolution of cyclooxygenase-targeting anti-inflammatory agents. Traditional nonsteroidal anti-inflammatory drugs (NSAIDs) possess both nonselective and selective inhibitory properties, which differently result in severe side effects, the most commonly reported of which are gastrointestinal irritation, ulceration and bleeding mostly attributed to the inhibition of the COX-1 enzyme.^11,12^ The development of selective COX-2 inhibitors represented a major advance in anti-inflammatory drug therapy. Celecoxib, the first selective COX-2 inhibitor approved for clinical use, was introduced in 1999.^13,14^ It effectively reduces inflammation and pain while producing fewer gastrointestinal adverse effects than non-selective NSAIDs. However, concerns regarding the cardiovascular risks associated with prolonged use of certain coxibs have emphasized the need to develop new selective COX-2 inhibitors with improved safety profiles and pharmacological properties.^15,16^ Over the past decades, considerable research has been devoted to the development of nonsteroidal anti-inflammatory drugs (NSAIDs) with improved efficacy and safety profiles. Particular attention has been directed toward the discovery of selective COX-2 inhibitors that retain anti-inflammatory activity while minimizing the gastrointestinal adverse effects associated with non-selective NSAIDs.^17^ Nitrogen- and oxygen-containing heterocycles, particularly isoxazolidine derivatives, represent indispensable scaffolds in modern drug design due to their electron-rich nature and diverse biological potential.^18,19^ The 1,3-dipolar cycloaddition (1,3-DC) of nitrones with alkenes stands as a premier synthetic methodology, offering a highly efficient route to functionalized isoxazolidines. Beyond their direct application, these reactions provide access to intricate carbon architectures and serve as a robust gateway to γ-amino alcohols, β-lactams, and β-amino acids—key intermediates that are vital in the total synthesis of complex organic molecules.^20,21^ A major advantage of this methodology is its high regio- and stereoselectivity, which is governed by the electronic and steric properties of both the nitrone and the dipolarophile. This selectivity enables the formation of multiple contiguous stereocenters in a single synthetic step, affording structurally defined products in high stereochemical purity. Specifically, the diastereoselective nature of this cycloaddition ensures the formation of specific isomers, while the regioselectivity dictates the orientation of the incoming dipolarophile, which is crucial for optimizing interactions with biological macromolecules. Furthermore, the structural rigidity of isoxazolidine systems restricts conformational flexibility, ensuring predictable stereochemical behavior. Such structural precision has underpinned their success as potent antimicrobial, antifungal,^22,23^ anticonvulsant, antiviral,^24^ antioxidant,^25^ anti-tuberculosis, anti-inflammatory, and antitumor agents.^26^ Consequently, the rational design of selective COX-2 inhibitors remains an effective strategy for improving drug-like properties and developing novel therapeutic candidates for the treatment of inflammatory diseases. In this work, the design, synthesis and biological evaluation of a novel series of tricyclic isoxazolidine–imidazole derivatives (3a–l) with varying substituted phenyl groups are reported. These were synthesized *via* regio- and diastereoselective 1,3-dipolar cycloaddition reactions between C-aryl-3-methylphenyl nitrones with 1-vinylimidazole to afford structurally well-defined isoxazolidine analogues. The resultant synthesized compounds were fully characterized using spectroscopic techniques and consequently evaluated for their inhibitory activity against COX-2 and COX-1 enzymes. In addition, and *in silico* ADMET analyses were conducted to assess their drug-likeness, oral bioavailability, and pharmacokinetic suitability. Also, molecular docking analyses were performed to elucidate the binding interactions of the most active compounds in the COX-2 active site, density functional theory calculations were conducted to study the electronic structure and reactivity of compounds 3a, 3e, and 3j.

## 2. Experimental

### 2.1. Materials and methods

All reagents and chemicals were purchased from Sigma-Aldrich (purity: 99–99.9%) and used without further purification. Reaction progress was monitored by thin-layer chromatography (TLC) using silica gel plates, with a mixture of ethyl acetate and hexane (7 : 3 v/v) as the mobile phase. Melting points of the synthesized derivatives were determined using a CL-726 digital melting point apparatus (IndiaMART, Noida, India). Fourier-transform infrared (FTIR) spectra were recorded on a Nicolet iS10 spectrophotometer (Thermo Scientific, Waltham, MA, USA) equipped with a diamond Attenuated Total Reflectance (ATR) accessory. The analysis was performed over a range of 4000–500 cm^−1^ with a resolution of 4 cm^−1^ and an accumulation of 32 scans. ^1^H and ^13^C nuclear magnetic resonance (NMR) spectra were acquired on a JEOL ECP400 spectrometer (JEOL Ltd, Tokyo, Japan) operating at 400 MHz. Chemical shifts are reported in parts per million (ppm) relative to tetramethylsilane (TMS) as an internal standard. Structural characterization was based on ^1^H and ^13^C NMR data and coupling constants (*J*) are given in Hz. Liquid chromatography-mass spectrometry (LC-MS) analysis was carried out using an electrospray ionization (ESI) source operated in positive ion mode. Mass spectra were acquired in Q1 scan mode using a quadrupole mass analyzer over an *m*/*z* range of 100–700. The analytical method employed in this study involved using an Agilent 1260 HPLC instrument equipped with a Diode-Array detector, and the resulting chromatographic data were analyzed using the Agilent ChemStation software.

### 2.2. General procedures

The synthesis of nitrones was accomplished *via* a two-step procedure.

#### 2.2.1. Step 1: Synthetic procedures of preparation of *N*-(3-methylphenyl) hydroxylamine

All procedures were modified and derived from ref. 27 Ammonium chloride (25 g, 470 mmol) and 3-nitrotoluene (48.59 g, 410 mmol) were placed in (600 mL) water and (200 mL) methanol in a three-necked round bottomed flask. This yellow solution was stirred with a mechanical stirrer within a duration of 60 minutes and within a period of 30 minutes (62 g, 1529 mmol) of zinc powder was gradually added. After 15 min stirring was stopped, and the reaction was considered finished as indicated by a decrease in the temperature of the yellow/grey reaction mixture, the solution suction filtered whilst still hot to eliminate the zinc oxide and washed with hot distilled water. The filtrate was saturated with sodium chloride (NaCl) and kept in an ice bath for 2 h in the dark. The product was extracted with diethyl ether (3 × 100 mL). The combined organic layer was dried over anhydrous sodium sulfate, and the solvent was removed under reduced pressure to afford *N*-(3-methylphenyl)hydroxylamine. The product was further dried under vacuum to give *N*-(3-methylphenyl)hydroxylamine as light-yellow needle-shaped crystals in 98% yield.

#### 2.2.2. Step 2: preparation of nitrones (1a–l)

Nitrones were synthesized according to the reported procedures^25,26^ with minor modifications. *N*-(3-methylphenyl)hydroxylamine (739 mg, 6 mmol) was dissolved in absolute ethanol (20 mL), and the mixture was stirred and heated to 40 °C. An additional 10 mL of absolute ethanol was added gradually to ensure complete dissolution. Sodium acetate (737 mg, 9 mmol) was then added, followed by the dropwise addition of the appropriate substituted benzaldehyde. The reaction mixture was stirred magnetically at room temperature in the dark overnight. The reaction progress was monitored by TLC. Upon completion, the precipitated nitrone was collected by filtration, washed with cold ethanol, dried under vacuum, and recrystallized from hot ethanol.

##### 2.2.2.1. Preparation of (*Z*)-3-methyl-*N*-(4-sulfamoylbenzylidene)aniline oxide (1a)

4-Formylbenzenesulfonamide (1.11 g, 6 mmol) was used for the synthesis as described in the general procedure. The mixture was stirred for (24 h). Molecular formula: C_14_H_14_N_2_O_3_S; *M*_W_: 290.34 g mol^−1^; appearance: yellowish white; Yield: 80%; m. *p* = syrup; *R*_f_ = 0.58. ^1^H NMR (400 MHz, DMSO) *δ* (ppm): 7.80 (d, *J* = 7.9 Hz, 1H), 7.64–7.59 (m, 2H), 7.32 (s, 1H, (CH=N)), 7.22 (s, 2H, NH_2_), 7.00–6.95 (m, 2H), 6.90 (d, *J* = 4.3 Hz, 1H), 6.74 (s, 1H), 6.51 (d, *J* = 14.6 Hz, 1H), 2.13 (s, 3H). ^13^C NMR (101 MHz, DMSO) *δ* (ppm): 145.1, 140.1, 136.8, 135.2, 131.9, 131.5, 131.0, 130.1, 128.9, 122.7, 121.0, 22.3.

##### 2.2.2.2. Preparation of (*Z*)-*N*-(4-(dimethylamino)benzylidene)-3-methylaniline oxide (1b)

4-*N*-Dimethyl benzaldehyde (895 mg, 6 mmol) was used for the synthesis as described in the general procedure. The mixture was stirred for (24 h). Molecular formula: C_16_H_18_N_2_O; *M*_W_: 254.33 g mol^−1^; appearance: brown; yield: 72%; m. *p* = 108–110; *R*_f_ = 0.65. ^1^H NMR (400 MHz, DMSO) *δ* (ppm): 8.05–8.00 (m, 2H), 7.96 (s, 1H, (CH=N), 7.87–7.85 (m, 2H), 7.41–7.37 (m, 1H), 7.32–7.29 (m, 1H), 6.98 (d, *J* = 6.9 Hz, 2H), 3.66 (s, 6H), 2.05 (s, 3H). ^13^C NMR (101 MHz, DMSO) *δ* (ppm): 152.1, 145.1, 140.1, 135.6, 131.9, 130.5, 128.9, 125.2, 122.7, 121.0, 112.6, 53.6, 40.1.

##### 2.2.2.3. Preparation of (*Z*)-3-methyl-*N*-(2-nitrobenzylidene)aniline oxide (1c)

2-Nitrobenzaldehyde (907 mg, 6 mmol) was used for the synthesis as described in the general procedure. The mixture was stirred for (24 h). Molecular formula: C_14_H_12_N_2_O_3_; *M*_W_: 256.26 g mol^−1^; appearance: yellow; yield: 88%; m. *p* = 86–88; *R*_f_ = 0.80. ^1^H NMR (400 MHz, DMSO) *δ* (ppm): 8.20 (s, 1H, (CH=N)), 8.18–8.14 (m, 2H), 8.06–7.99 (m, 2H), 7.73–7.60 (m, 2H), 7.42–7.35 (m, 1H), 7.34–7.28 (m, 1H), 2.34 (s, 3H). ^13^C NMR (101 MHz, DMSO) *δ* (ppm):145.90, 144.74, 140.12, 133.01, 131.98, 131.35, 130.43, 129.73, 128.93, 127.10, 125.86, 122.74, 120.99, 21.37.

##### 2.2.2.4. Preparation of (*Z*)-*N*-(4-fluorobenzylidene)-3-methylaniline oxide (1d)

4-Floro benzaldehyde (745 mg, 6 mmol) was used for the synthesis as described in the general procedure. The mixture was stirred for (24 h). Molecular formula: C_14_H_12_FNO; *M*_W_: 229.25 g mol^−1^; appearance: light yellow; yield: 86%; m. *p* = 160–162 °C; *R*_f_ = 0.57. ^1^H NMR (400 MHz, DMSO) *δ* (ppm): 8.09–7.97 (m, 4H), 7.94 (s, 1H, (CH=N)), 7.42–7.28 (m, 4H), 2.15 (s, 3H). ^13^C NMR (101 MHz, DMSO) *δ* (ppm): 163.0, 161.0, 145.1, 140.1, 135.6, 132.2, 132.2, 131.9, 129.4, 129.4, 128.9, 122.7, 121.0, 117.0, 116.9, 21.3.

##### 2.2.2.5. Preparation of (*Z*)-*N*-(4-hydroxy-3-methoxybenzylidene)-3-methylaniline oxide (1e)

4-Hydroxy-3-methoxy benzaldehyde (913 mg, 6 mmol) was used for the synthesis as described in the general procedure. The mixture was stirred for (24 h). Molecular formula: C_15_H_15_NO_3_; *M*_W_: 257.28 g mol^−1^, appearance: light yellow; yield: 90%; m. *p* = 171–173 °C; *R*_f_ = 0.40. ^1^H NMR (400 MHz, DMSO) *δ* (ppm): 8.13 (d, *J* = 1.9 Hz, 1H), 7.67 (s, 1H, (CH=N)), 7.57 (m, 2H), 7.56 (d, *J* = 1.9 Hz, 2H), 7.55 (d, *J* = 1.9 Hz, 2H), 6.84 (d, *J* = 8.3 Hz, 2H), 3.77 (s, 3H), 2.28 (s, 3H). ^13^C NMR (101 MHz, DMSO) *δ* (ppm): 149.6, 149.5, 148.5, 139.4, 132.9, 131.5, 129.9, 126.1, 120.7, 119.2, 117.4, 114.4, 55.9, 23.3.

##### 2.2.2.6. Preparation of (*Z*)-*N*-(4-chlorobenzylidene)-3-methylaniline oxide (1f)

4-Chloro benzaldehyde (843 mg, 6 mmol) was used for the synthesis as described in the general procedure. The mixture was stirred for (24 h). Molecular formula: C_14_H_12_ClNO; *M*_W_: 245.70 g mol^−1^, appearance: light yellow; yield: 87%; m. *p* = 68–71 °C; *R*_f_ 0.72. ^1^H NMR (400 MHz, DMSO) *δ* (ppm): 8.05–8.01 (m, 2H), 7.92 (s, 1H, (CH=N)), 7.89–7.86 (m, 2H), 7.47–7.45 (d, *J* = 8.7 Hz, 2H), 7.41–7.37 (m, 1H), 7.31–7.29 (dt, *J* = 7.8, 1.6 Hz, 1H), 2.05 (s, 3H).^13^C NMR (101 MHz, DMSO) *δ* (ppm): 145.1, 140.1, 136.8, 135.2, 131.9, 131.5, 131.0, 130.1, 128.9, 122.7, 121.0, 21.3.

##### 2.2.2.7. Preparation of (*Z*)-*N*-(2-hydroxybenzylidene)-3-methylaniline oxide (1g)

2-Hydroxy benzaldehyde (733 mg, 6 mmol) was used for the synthesis as described in the general procedure. The mixture was stirred for (24 h). Molecular formula: C_14_H_13_NO_2_; *M*_W_: 227.26 g mol^−1^; appearance: light yellow; yield: 65%; m. *p* = 134–136 °C; *R*_f_ = 0.48. ^1^H NMR (400 MHz, DMSO) *δ* (ppm): 8.57 (s, 1H), 8.19 (s, 1H, (CH=N)), 8.07–7.99 (m, 2H), 7.96 (d, *J* = 8.2 Hz, 1H), 7.42–7.35 (m, 1H), 7.34–7.27 (m, 2H), 7.17–7.10 (m, 2H), 2.37 (s, 3H). ^13^C NMR (101 MHz, DMSO) *δ* (ppm): 157.87, 144.75, 140.12, 136.25, 132.76, 131.98, 131.05, 128.94, 122.73, 122.25, 121.82, 120.98, 116.28, 21.37.

##### 2.2.2.8. Preparation of (*Z*)-*N*-(4-hydroxybenzylidene)-3-methylaniline oxide (1h)

4-Hydroxy benzaldehyde (733 mg, 6 mmol) was used for the synthesis as described in the general procedure. The mixture was stirred for (24 h). Molecular formula: C_14_H_13_NO_2_; *M*_W_: 227.26 g mol^−1^, appearance: light brown; yield: 83%; m. *p* = 97–100 °C; *R*_f_ = 0.54. ^1^H NMR (400 MHz, DMSO) *δ* (ppm): 8.06–8.01 (m, 2H), 7.95–7.92 (d, *J* = 10.7 Hz, 3H), 7.68 (s, 1H, (CH=N)), 7.41–7.37 (m, 1H), 7.32–7.29 (m, 1H), 7.03–7.01 (d, *J* = 7.8 Hz, 2H), 2.06 (s, 3H).^13^C NMR (101 MHz, DMSO) *δ* (ppm): 155.2, 145.1, 140.1, 135.7, 131.9, 131.8, 128.9, 125.7, 122.7, 121.0, 116.9, 21.1.

##### 2.2.2.9. Preparation of (*Z*)-*N*-(4-methoxybenzylidene)-3-methylaniline oxide (1i)

4-Methoxy benzaldehyde (817 mg, 6 mmol) was used for the synthesis as described in the general procedure. The mixture was stirred for (24 h). Molecular formula: C_15_H_15_NO_2_; *M*_W_: 241.29 g mol^−1^; appearance: brown; yield: 75%; m. *p* = 60–63 °C; *R*_f_ = 0.55.

##### 2.2.2.10. Preparation of (*Z*)-*N*-(4-bromobenzylidene)-3-methylaniline oxide (1j)

4-Bromo benzaldehyde (1.11 g, 6 mmol) was used for the synthesis as described in the general procedure. The mixture was stirred for (24 h). Molecular formula: C_14_H_12_BrNO; *M*_W_: 290.16 g mol^−1^, appearance: dark brown; yield: 90%; m. *p* = 76–78 °C; *R*_f_ = 0.52.

##### 2.2.2.11. Preparation of (*Z*)-*N*-(4-isopropylbenzylidene)-3-methylaniline oxide (1k)

4-Isopropyl benzaldehyde (889 mg, 6 mmol) was used for the synthesis as described in the general procedure. The mixture was stirred for (24 h); molecular formula: C_17_H_19_NO; *M*_W_: 253.34 g mol^−1^; appearance: light yellow; yield: 85%; m. *p* = syrup; *R*_f_ = 0.66.

##### 2.2.2.12. Preparation of (*Z*)-3-methyl-*N*-(4-methylbenzylidene)aniline oxide (1l)

4-Methylbenzaldehyde (721 mg, 6 mmol) was used for the synthesis as described in the general procedure. The mixture was stirred for (24 h). Molecular formula: C_15_H_15_NO; *M*_W_: 225.29 g mol^−1^, appearance: light yellow; yield: 72%; m. *p* = syrup; *R*_f_ = 0.68. ^1^H NMR (400 MHz, DMSO) *δ* (ppm): 8.05–8.01 (m, 2H), 7.92 (s, 1H, (CH=N)), 7.85–7.82 (m, 2H), 7.39 (dd, *J* = 8.6, 7.9 Hz, 1H), 7.30–7.28 (m, 3H), 2.51 (s, 3H), 2.32 (s, 3H). ^13^C NMR (101 MHz, DMSO) *δ* (ppm): 142.4, 140.1, 135.6, 135.0, 134.2, 129.3, 128.9, 128.9, 128.2, 122.7, 121.0, 21.6, 21.3.

#### 2.2.3. The general synthetic procedure of isoxazolidine derivatives (3a–l)

The target isoxazolidine derivatives were synthesized following modified literature protocols.^25,26^ In a 100 mL round-bottom flask, the appropriate substituted nitrones (1.5 mmol, 1.0 equiv.) were dissolved in anhydrous DMF (25 mL) under constant stirring. Subsequently, 1-vinylimidazole (1.5 mmol, 1.0 equiv.) was added to the mixture. The reaction was maintained under reflux at 110 °C for 15–25 hours, with the progress monitored by thin-layer chromatography (TLC). Upon completion, the reaction mixture was cooled to room temperature, and the solvent was removed under reduced pressure. The resulting residue was partitioned between distilled water (20 mL) and chloroform (30 mL). The organic phase was separated, dried over anhydrous sodium sulphate (Na_2_ SO_4_), and filtered. After evaporation of the solvent, the crude product was purified *via* silica gel column chromatography, using an ethyl acetate : hexane (4 : 6, v/v) mixture as the eluent, to afford the pure isoxazolidine compounds.

##### 2.2.3.1. Preparation of isoxazolidines (3a–l) according to the previous method

###### 2.2.3.1.1. Preparation of 4-((3*R*,4*R*)-4-(1*H*-imidazole-1-yl)-2-(*m*-tolyl)isoxazolidin-3-yl)benzenesulfonamide (3a)

Nitrone (1a) (435 mg, 1.5 mmol) and 1-vinylimidazole (141.5 mg, 1.5 mmol) were reacted following the general synthetic procedure. The reaction mixture was stirred for 20 h. Molecular formula: C_19_H_20_N_4_O_3_S; *M*_W_: 384.45 g mol^−1^; purity = 98.07%; yield: 80%; *R*_f_ = 0.75; appearance: light brown syrup. IR (KBr, cm^−1^): *υ*_max_ = 3367(NH_2_), 3094 (C–H), 1645 (C=N), 1578 (C=C), 1530–1445 (aromatic), 1373 (C–O), 1171 (N–O), 1092, 1014 (C–N), 910, 812, 754 (*p*-disubstituted benzene).^1^H NMR (400 MHz, DMSO-*d*_6_) *δ* (ppm): 8.74 (s, 1H,H6), 8.24 (d, *J* = 15.3 Hz, 1H, H8), 7.66 (d, *J* = 15.7 Hz, 1H, H7), 7.29 (s, 2H, NH_2_), 7.27–6.90 (m, 7H, aromatic H),6.83 (d, *J* = 8.8 Hz, 1H, aromatic H), 5.83 (dd, *J* = 10.8, 8.8 Hz, 1H, H5), 5.56 (dd, *J* = 10.5, 4.3 Hz, 1H, H5^\^), 4.28–4.24 (dt, *J* = 10.5, 1.7 Hz, 1H, H4), 4.03–4.01 (dt, *J* = 10.8, 1.8 Hz, 1H, H3), 3.00 (s, 3H, CH_3_).^13^C NMR (101 MHz, DMSO-*d*_6_) *δ* (ppm): 149.1, 136.9, 134.7, 130.3, 129.2, 127.06, 124.9, 123.9, 123.6, 116.9, 83.7, 76.3, 56.5, 22.9. LC-MS *m*/*z*: [M + H]^+^ found 385.39 calculated for C_19_H_20_N_4_O_3_S 384.45.

###### 2.2.3.1.2. Preparation of 4-((3*R*,4*R*)-4-(1*H*-imidazole-1-yl)-2-(*m*-tolyl)isoxazolidin-3-yl)-*N*,*N*-dimethylaniline (3b)

Nitrone (1b) (381 mg, 1.5 mmol) and 1-vinylimidazole (141.5 mg, 1.5 mmol) were reacted according to the general synthetic procedure. The reaction mixture was stirred for 20 h to afford the target compound. Molecular formula: C_21_H_24_N_4_O; *M*_W_: 348.44 g mol^−1^; purity = 98.87%; yield: 96%; *R*_f_ = 0.89; appearance: red syrup. IR (KBr, cm^−1^): *υ*_max_ = 3550 (N–CH_3_), 3040 (C–H), 1649 (C=N), 1593 (C=C), 1541–1448 (aromatic C=C), 1244 (C–O), 1167 (N–O), 1042, 1040 (C–N), 912, 818, 755 (*p*-disubstituted benzene).^1^H NMR (400 MHz, DMSO-*d*_6_) *δ* (ppm): 9.61 (s, 1H, H6), 9.08 (d, *J* = 15.3 Hz, 1H, H8), 8.02 (d, *J* = 15.5 Hz, 1H, H7), 7.64–6.75 (m, 8H, aromatic H), 5.48–5.45 (dd, *J* = 10.8, 8.8 Hz, 1H, H5), 5.08–5.05 (dd, *J* = 10.5, 4.3 Hz, 1H, H5^\^), 4.28–4.26(dt, *J* = 10.7, 1.2 Hz, 1H, H4), 4.01 (dt, *J* = 10.4, 1.7 Hz, 1H, H3), 2.99 (s, 6H, N–CH_3_), 2.75 (s, 3H, CH_3_).^13^C NMR (101 MHz, DMSO-*d*_6_) *δ* (ppm): 159.1, 149.9, 146.9, 138.4, 135.2, 130.4, 129.9, 129.5, 128.8, 123.6, 118.1, 117.8, 116.1, 112.9, 112.8, 78.2, 77.1, 52.3, 40.3, 21.8. LC-MS *m*/*z*: [M + H]^+^ found 349.11 calculated for C_21_H_24_N_4_O 348.44.

###### 2.2.3.1.3. Preparation of (3*R*,4*R*)-4-(1*H*-imidazole-1-yl)-3-(2-nitrophenyl)-2-(*m*-tolyl)isoxazolidine (3c)

Nitrone (1c) (384 mg, 1.5 mmol) and 1-vinylimidazole (141.5 mg, 1.5 mmol) were reacted following the general synthetic procedure. The reaction mixture was stirred for 25 h to afford the desired product. Molecular formula: C_19_H_18_N_4_O_3_; *M*_W_: 350.37 g mol^−1^; purity = 96.88%; yield: 78%; *R*_f_ = 0.92; appearance: yellow syrup. IR (KBr, cm^−1^): *υ*_max_ = 3048 (C–H), 1645 (C=N), 1576 (C=C), 1535–1443 (aromatic C=C), 1362, 1313 (NO_2_), 1304 (C–O), 1167 (N–O), 1086, 1040 (C–N), 910, 866, 748 (*o*-disubstituted benzene).^1^H NMR (400 MHz, DMSO-*d*_6_) *δ* (ppm): 8.81 (dd, *J* = 8.6, 1.2 Hz, 1H, aromatic H), 8.52 (s, 1H, H6), 7.78 (d, *J* = 15.2 Hz, 1H, H8), 7.70 (d, *J* = 15.4 Hz, 1H, H7),7.64–6.96 (m, 7H, aromatic H), 5.76–5.74 (dd, *J* = 10.4, 8.6 Hz, 1H,H5), 5.27–5.26(dd, *J* = 10.1, 4.7 Hz, 1H, H5^\^), 4.05–4.03 (dt, *J* = 9.9, 1.8 Hz, 1H, H4), 3.83 (d, *J* = 10.8, Hz, 1H,H3), 3.37 (s, 3H, CH_3_).^13^C NMR (101 MHz, DMSO-*d*_6_) *δ* (ppm): 146.7, 146.3, 138.2, 135.2, 132.2, 131.8, 129.1, 128.3, 124.3, 123.8, 117.9, 116.0, 83.6, 74.1, 57.1, 22.4. LC-MS *m*/*z*: [M + H]^+^ found 351.05 calculated for C_19_H_18_N_4_O_3_ 350.37.

###### 2.2.3.1.4. Preparation of (3*R*,4*R*)-3-(4-fluorophenyl)-4-(1*H*-imidazole-1-yl)-2-(*m*-tolyl)isoxazolidine (3d)

Nitrone (1d) (344 mg, 1.5 mmol) and 1-vinylimidazole (141.5 mg, 1.5 mmol) were reacted according to the general synthetic procedure. The reaction mixture was stirred for 20 h to afford the target compound. Molecular formula: C_19_H_18_FN_3_O; *M*_W_: 323.36 g mol^−1^; purity = 95.88%; yield: 91%; *R*_f_ = 0.83; appearance: yellowish-brown syrup. IR (KBr, cm^−1^): *υ*_max_ = 3028 (C–H), 1649 (C=N), 1590 (C=C), 1552, 1439 (aromatic C=C), 1319 (C–O), 1241 (C–F), 1165 (N–O), 1084, 1042 (C–N), 982, 849, 779 (*p*-disubstituted benzene).^1^H NMR (400 MHz, DMSO-*d*_6_) *δ* (ppm): 8.44 (s, 1H, H6), 7.62 (d, *J* = 15.3 Hz, 1H, H8), 7.20 (d, *J* = 15.2 Hz, 1H, H7), 7.26–6.83 (m, 8H, aromatic H), 5.60 (dd, *J* = 10.4, 8.7 Hz, 1H, H5), 5.14 (dd, *J* = 10.1, 4.2 Hz, 1H, H5^\^), 4.29 (dt, *J* = 10.7, 1.4 Hz, 1H, H4), 4.07 (dt, *J* = 10.3, 1.2 Hz, 1H, H3), 2.54 (s, 3H, CH_3_).^13^C NMR (101 MHz, DMSO-*d*_6_) *δ* (ppm): 156.3, 154.4, 146.9, 138.4, 135.2, 134.2, 132.3, 131.1, 129.4, 128.7, 123.9, 121.5, 119.9, 119.4, 118.6, 83.3, 74.6, 56.4, 22.3. LC-MS *m*/*z*: [M + H]^+^ found 324.10 calculated for C_19_H_18_FN_3_O 323.36.

###### 2.2.3.1.5. Preparation of 4-((3*R*,4*R*)-4-(1*H*-imidazole-1-yl)-2-(*m*-tolyl)isoxazolidin-3-yl)-2-methoxyphenol (3e)

Nitrone (1e) (386 mg, 1.5 mmol) and 1-vinylimidazole (141.5 mg, 1.5 mmol) were reacted according to the general synthetic procedure. The reaction mixture was stirred for 30 h to afford the target compound. Molecular formula: C_20_H_21_N_3_O_3_; *M*_W_: 351.40 g mol^−1^; purity = 98.85%; yield: 79%; *R*_f_ = 0.76; appearance: brown syrup. IR (KBr, cm^−1^): *υ*_max_ = 3345 (O–H), 3053, 2847 (C–H), 1649 (C=N), 1576 (C=C), 1545, 1439 (aromatic C=C), 1371, 1304, 1246 (C–O), 1171 (N–O), 1090, 1013 (C–N), 957, 818 (*p*-substituted), 752, 734 (*m*-trisubstituted benzene).^1^H NMR (400 MHz, DMSO-*d*_6_) *δ* (ppm): 8.51 (s, 1H, H6), 7.97 (d, *J* = 15.4 Hz, 1H, H8), 7.96 (d, *J* = 8.3 Hz, 1H, aromatic H), 7.94 (d, *J* = 15.1 Hz, 1H, H7), 7.93 (s, 1H, OH), 7.88–7.25 (m, 6H, aromatic H), 5.40–5.38 (dd, *J* = 10.3, 8.4 Hz, 1H, H5), 5.06–5.05 (dd, *J* = 10.3, 4.5 Hz, 1H, H5^\^), 4.29–4.28 (dt, *J* = 10.5, 1.6 Hz, 1H, H4), 4.07–4.05 (dt, *J* = 10.8, 1.8 Hz, 1H, H3), 3.81 (s, 3H, OCH_3_), 2.54 (s, 3H, CH_3_).^13^C NMR (101 MHz, DMSO-*d*_6_) *δ* (ppm): 168.2, 148.6, 147.0, 138.4, 135.2, 131.1, 129.5, 128.8, 123.6, 118.1, 117.8, 116.0, 114.9, 113.6, 83.4, 76.2, 62.8, 48.1, 21.8. LC-MS *m*/*z*: [M + H]^+^ found 352.35 calculated for C_20_H_21_N_3_O_3_ 351.40.

###### 2.2.3.1.6. Preparation of (3*R*,4*R*)-3-(4-chlorophenyl)-4-(1*H*-imidazole-1-yl)-2-(*m*-tolyl)isoxazolidine (3f)

Nitrone (1f) (369 mg, 1.5 mmol) and 1-vinylimidazole (141.5 mg, 1.5 mmol) were reacted following the general synthetic procedure. The reaction mixture was stirred for 18 h to afford the desired product. Molecular formula: C_19_H_18_ClN_3_O; *M*_W_: 339.82 g mol^−1^; purity = 98.87%; yield: 95%; *R*_f_ = 0.81; appearance: light yellow syrup. IR (KBr, cm^−1^): *υ*_max_ = 3035 (C–H), 1646 (C=N), 1590 (C=C), 1553–1446 (aromatic C=C), 1346 (C–O), 1177 (N–O), 1088, 1018 (C–N), 959, 902, 836 (*p*-disubstituted benzene), 757 (C–Cl).^1^H NMR (400 MHz, DMSO-*d*_6_) *δ* (ppm): 8.08 (s, 1H, H6), 7.62 (d, *J* = 15.4 Hz, 1H, H8), 7.48 (d, *J* = 15.3 Hz, 1H, aromatic H), 7.37 (d, *J* = 15.2 Hz, 1H, H7), 7.20–6.83 (m, 7H, aromatic H), 5.28–5.26 (dd, *J* = 10.4, 8.7 Hz, 1H, H5), 5.12–5.11 (dd, *J* = 10.1, 4.2 Hz, 1H, H5^\^), 4.28–4.26 (dt, *J* = 10.2, 1.7 Hz, 1H, H4), 4.07–4.05 (dt, *J* = 10.3, 1.2 Hz, 1H, H3), 2.62 (s, 3H, CH_3_).^13^C NMR (101 MHz, DMSO-*d*_6_) *δ* (ppm): 154.8, 146.9, 138.4, 135.6, 134.7, 131.3, 129.5, 128.8, 128.6, 123.6, 118.1, 117.8, 116.1, 81.9, 69.4, 53.7, 24.4. LC-MS *m*/*z*: [M + H]^+^ found 340.35 calculated for C_19_H_18_ClN_3_O 339.82.

###### 2.2.3.1.7. Preparation of 2-((3*R*,4*R*)-4-(1*H*-imidazole-1-yl)-2-(*m*-tolyl)isoxazolidin-3-yl)phenol (3g)

Nitrone (1g) (341 mg, 1.5 mmol) was reacted with 1-vinylimidazole (141.5 mg, 1.5 mmol) according to the general procedure. The reaction mixture was stirred for 15 h to afford the target compound. Molecular formula: C_19_H_19_N_3_O_2_; *M*_W_: 321.37 g mol^−1^; purity = 98.79%; yield: 86%; *R*_f_ = 0.87; appearance: light yellow syrup. IR (KBr, cm^−1^): *υ*_max_ = 3317 (O–H), 2984 (C–H), 1604 (C=N), 1589 (C=C), 1514–1400 (aromatic C=C), 1312, 1261 (C–O), 1144 (N–O), 1086, 1040 (C–N), 944, 827, 761 (*o*-disubstituted benzene).^1^H NMR (400 MHz, DMSO-*d*_6_) *δ* (ppm): 8.77 (s, 1H, H6), 8.42–8.40 (d, *J* = 15.5 Hz, 1H, H8), 8.08 (d, *J* = 15.1 Hz, 1H, H7), 8.00–7.73 (m, 6H, aromatic H), 7.59 (s, 1H, OH), 7.58–7.54 (m, 2H, aromatic H), 5.56–5.50 (dd, *J* = 10.4, 8.6 Hz, 1H, H5), 5.26–5.24 (dd, *J* = 10.1, 4.7 Hz, 1H, H5^\^), 4.28–4.26 (m, 1H, H4), 4.04 (dt, *J* = 10.8, 1.8 Hz, 1H, H3), 2.54 (s, 3H, CH_3_).^13^C NMR (101 MHz, DMSO-*d*_6_) *δ* (ppm):153.5, 146.1, 138.4, 135.4, 130.6, 129.5, 129.2, 128.8, 123.6, 123.5, 121.0, 118.0, 117.8, 116.0, 115.9, 74.2, 67.9, 57.2, 21.4. LC-MS *m*/*z*: [M + H]^+^ found 322.08 calculated for C_19_H_19_N_3_O_2_ 321.37.

###### 2.2.3.1.8. Preparation of 4-((3*R*,4*R*)-4-(1*H*-imidazole-1-yl)-2-(*m*-tolyl)isoxazolidin-3-yl)phenol (3h)

Nitrone (1h) (341 mg, 1.5 mmol) was reacted with 1-vinylimidazole (141.5 mg, 1.5 mmol) following the general procedure. The reaction mixture was stirred for 20 h to afford the desired product. Molecular formula: C_19_H_19_N_3_O_2_; *M*_W_: 321.37 g mol^−1^; purity = 95.00%; yield: 90%; *R*_f_ = 0.50; appearance: brown syrup. IR (KBr, cm^−1^): *υ*_max_ = 3365 (O–H), 3046 (C–H), 1649 (C=N), 1574 (C=C), 1549–1445 (aromatic C=C), 1369, 1302 (C–O), 1167 (N–O), 1090, 1010 (C–N), 952, 814, 752 (*p*-disubstituted benzene).^1^H NMR (400 MHz, DMSO-*d*_6_) *δ* (ppm): 8.72 (s, 1H, H6), 8.59 (s, 1H, OH), 8.25 (d, *J* = 15.8 Hz, 1H, H8), 7.70 (d, *J* = 15.5 Hz, 1H, H7), 7.29–6.80 (m, 8H, aromatic H), 5.59 (dd, *J* = 10.3, 8.4 Hz, 1H, H5), 5.17 (dd, *J* = 10.3, 4.5 Hz, 1H, H5^\^), 4.32 (dt, *J* = 10.8, 1.8 Hz, 1H, H4), 4.08 (dt, *J* = 10.8, 1.8 Hz, 1H, H3), 2.34 (s, 3H, CH_3_).^13^C NMR (101 MHz, DMSO-*d*_6_) *δ* (ppm): 159.4, 149.3, 136.0, 134.3, 131.2, 130.3, 129.9, 128.7, 125.2, 124.9, 124.6, 117.9, 84.4, 76.3, 54.6, 22.9. LC-MS *m*/*z*: [M + H]^+^ found 322.32 calculated for C_19_H_19_N_3_O_2_ 321.37.

###### 2.2.3.1.9. Preparation of (3*R*,4*R*)-4-(1*H*-imidazole-1-yl)-3-(4-methoxyphenyl)-2-(*m*-tolyl)isoxazolidine (3i)

Nitrone (1i) (362 mg, 1.5 mmol) was allowed to react with 1-vinylimidazole (141.5 mg, 1.5 mmol) according to the general synthetic protocol. The reaction mixture was stirred for 25 h to yield the target compound. Molecular formula: C_20_H_21_N_3_O_2_; *M*_W_: 335.40 g mol^−1^; purity = 97.55%; yield: 87%; *R*_f_ = 0.67; appearance: brown syrup. IR (KBr, cm^−1^): *υ*_max_ = 3145 (O–CH_3_), 3012 (C–H), 1651 (C=N), 1581 (C=C), 1534–1448 (aromatic C=C), 1316, 1268 (C–O), 1172 (N–O), 1094, 1016 (C–N), 954, 822, 776 (*p*-disubstituted phenyl).^1^H NMR (400 MHz, DMSO-*d*_6_) *δ* (ppm): 9.08 (s, 1H, H6), 8.02 (d, *J* = 15.8 Hz, 1H, H8), 7.64 (d, *J* = 15.5 Hz, 1H, H7), 7.63–6.76 (m, 8H, aromatic H), 5.50–5.48 (dd, *J* = 10.3, 8.4 Hz, 1H, H5), 5.23–5.21 (dd, *J* = 10.3, 4.5 Hz, 1H, H5^\^), 4.27–4.26 (m, 1H, H4), 4.11–4.09 (dt, *J* = 10.8, 1.8 Hz, 1H, H3), 3.64 (s, 3H, OCH_3_), 2.99 (s, 3H, CH_3_).^13^C NMR (101 MHz, DMSO-*d*_6_) *δ* (ppm): 158.9, 146.9, 138.4, 135.2, 131.1, 130.6, 129.5, 128.8, 123.6, 118.1, 117.8, 81.2, 74.1, 56.6, 55.4, 24.7. LC-MS *m*/*z*: [M + H]^+^ found 336.21 calculated for C_20_H_21_N_3_O_2_ 335.40.

###### 2.2.3.1.10. Preparation of (3*R*,4*R*)-3-(4-bromophenyl)-4-(1*H*-imidazole-1-yl)-2-(*m*-tolyl)isoxazolidine (3j)

Nitrone (1j) (435 mg, 1.5 mmol) was reacted with 1-vinylimidazole (141.5 mg, 1.5 mmol) following the general synthetic procedure. The reaction mixture was stirred for 24 h to afford the desired product. Molecular formula: C_19_H_18_BrN_3_O; *M*_W_: 384.27 g mol^−1^; purity = 98.13%; yield: 77%; *R*_f_ = 0.88; appearance: dark yellow syrup. IR (KBr, cm^−1^): *υ*_max_ = 3145 (C–H), 3056 (–CH_3_), 1649 (C=N), 1574 (C=C), 1549–1442 (aromatic C=C), 1369 (C–O), 1167 (N–O), 1090, 1058 (C–N), 992, 833, 801 (*p*-disubstituted phenyl), 688 (C–Br).^1^H NMR (400 MHz, DMSO-*d*_6_) *δ* (ppm): 8.43 (s, 1H, H6), 8.08 (d, *J* = 15.5 Hz, 1H, H8), 7.99–7.77 (m, 3H, aromatic H), 7.75 (d, *J* = 15.3 Hz, 1H, H7),7.73–7.25 (m, 5H, aromatic H), 6.27–6.25 (dd, *J* = 10.4, 8.7 Hz, 1H, H5), 5.51–5.50 (dd, *J* = 10.1, 4.2 Hz, 1H, H5^\^), 4.14 (m, 1H, H4), 3.53 (dt, *J* = 10.3, 1.2 Hz, 1H, H3), 2.54 (s, 3H, CH_3_).^13^C NMR (101 MHz, DMSO-*d*_6_) *δ* (ppm): 149.5, 137.4, 137.0, 134.8, 134.3, 133.8, 130.7, 129.8, 129.5, 125.2, 124.8, 118.3, 83.4, 67.7, 54.6, 23.5. LC-MS *m*/*z*: [M + H]^+^ found 385.10 calculated for C_19_H_18_BrN_3_O 384.27.

###### 2.2.3.1.11. Preparation of (3*R*,4*R*)-4-(1*H*-imidazole-1-yl)-3-(4-isopropylphenyl)-2-(*m*-tolyl)isoxazolidine (3k)

Nitrone (1k) (380 mg, 1.5 mmol) was reacted with 1-vinylimidazole (141.5 mg, 1.5 mmol) according to the general synthetic protocol. The reaction mixture was stirred for 26 h to afford the target compound. Molecular formula: C_22_H_25_N_3_O; *M*_W_: 347.45 g mol^−1^; purity = 96.25%; yield: 89%; *R*_f_ = 0.77; appearance: brown syrup. IR (KBr, cm^−1^): *υ*_max_ = 3194, 2972 (C–H), 1650 (C=N), 1573 (C=C), 1528–1420 (aromatic C=C), 1308 (C–O), 1163 (N–O), 1094, 1040 (C–N), 986, 856, 829 (*p*-disubstituted phenyl).^1^H NMR (400 MHz, DMSO-*d*_6_) *δ* (ppm): 8.27 (s, 1H, H6), 7.62 (d, *J* = 15.3 Hz, 1H, H8), 7.17–6.85 (m, 8H, aromatic H), 6.83 (d, *J* = 15.7 Hz, 1H, H7), 5.50 (dd, *J* = 10.8, 8.8 Hz,1H, H5), 5.13 (dd, *J* = 10.5, 4.3 Hz, 1H, H5^\^), 4.28 (m, 1H, H4), 4.05 (dt, *J* = 10.8, 1.8 Hz, 1H, H3), 2.86 (m, 1H, CH), 2.51 (s, 3H, CH_3_), 1.88 (d, *J* = 7.3 Hz, 6H, 2CH_3_). ^13^C NMR (101 MHz, DMSO-*d*_6_) *δ* (ppm): 149.0.146,9, 136.6, 134.7, 130.7, 129.2, 128.9, 127.0, 124.9, 123.9, 119.0, 118.0, 117.1, 83.7, 76.3, 56.5, 40.3, 29.5, 21.8. LC-MS *m*/*z*: [M + H]^+^found 348.22 calculated for C_22_H_25_N_3_O 347.45.

###### 2.2.3.1.12. Preparation of (3*R*,4*R*)-4-(1*H*-imidazole-1-yl)-2-(*m*-tolyl)-3-(p-tolyl)isoxazolidine (3l)

Nitrone (1l) (338 mg, 1.5 mmol) was reacted with 1-vinylimidazole (141.5 mg, 1.5 mmol) following the general synthetic procedure. The reaction mixture was stirred for 25 h to afford the desired product. Molecular formula: C_20_H_21_N_3_O; *M*_W_: 319.40 g mol^−1^; purity = 97.96%; yield: 92%; *R*_f_ = 0.78; appearance: light brown syrup. IR (KBr, cm^−1^): *υ*_max_ = 3375, 3102 (C–H), 1651 (C=N), 1574 (C=C), 1545–1445 (aromatic C=C), 1290 (C–O), 1177 (N–O), 1092, 1053 (C–N), 958, 825, 758 (*p*-disubstituted phenyl).^1^H NMR (400 MHz, DMSO-*d*_6_) *δ* (ppm): 8.80 (s, 1H, H6), 8.26 (d, *J* = 15.8 Hz, 1H, H8), 7.70 (d, *J* = 15.5 Hz, 1H, H7), 7.29–6.82 (m, 8H, aromatic H), 5.59 (dd, *J* = 10.3, 8.4 Hz, 1H, H5), 5.15 (dd, *J* = 10.3, 4.5 Hz, 1H, H5^\^), 4.75 (m, 1H, H4), 4.22 (dt, *J* = 10.8, 1.8 Hz, 1H, H3), 2.44 (s, 3H, CH_3_), 2.36 (s, 3H, CH_3_).^13^C NMR (101 MHz, DMSO-*d*_6_) *δ* (ppm): 148.9, 136.2, 134.5, 130.7, 130.3, 129.8, 128.1, 125.8, 124.8, 124.5, 117.9, 84.3, 76.3, 54.6, 29.5, 23.4. LC-MS *m*/*z*: [M + H]^+^ found 320.23 calculated for C_20_H_21_N_3_O 319.40.

### 2.3 Biological evaluation

#### 2.3.1. Enzyme inhibitory activity

The inhibitory effects against COX-1 and COX-2 were assessed using a colorimetric COX inhibitor screening assay kit (Cayman Chemical, USA) according to the manufacturer's protocol. Assays were performed in a final volume of 220 µL, and enzyme inhibition was quantified at 590 nm by measuring the formation of oxidized *N*,*N*,*N*′,*N*′-tetramethyl-*p*-phenylenediamine (TMPD) following incubation of ovine COX-1 or COX-2 with arachidonic acid. The enzymes were preincubated with the test compounds (3a–l) concentrations: 0.1, 1, 5, 10, 50 and 100 µM at 25 °C for 5 min before the addition of arachidonic acid (1.10 mM) and TMPD, followed by further incubation for 5 min at the same temperature. Celecoxib served as the reference inhibitor. COX inhibition (%) was calculated by comparing sample absorbance with the vehicle control after blank subtraction.^28,29^% Inhibition = [1 −(*A*_sample_ − *A*_blank_) / (*A*_vehicle control_ − *A*_blank_)] × 100.

The selectivity index (SI) was defined as the IC_50_ ratio of COX-1 to COX-2.SI = (COX-1_IC_50__/COX-2_IC_50__)

All experiments were conducted in triplicate, and data are expressed as mean values.

### 2.4. Computational studies

#### 2.4.1 ADME Study

The tested isoxazolidines were evaluated for their physicochemical properties and ADME profiles Absorption, Distribution, Metabolism and Excretion by the SwissADME platform.^30–32^ Key parameters including molecular weight (MW), hydrogen bond donors/acceptors, rotatable bonds and topological polar surface area (TPSA) were calculated to assess compliance with drug-likeness rules as lipeneski rule. Pharmacokinetic behavior was studed focusing on: Blood–Brain Barrier (BBB) and Gastrointestinal (GI) absorption permeability using the BOILED-Egg model. P-glycoprotein (P-gp) substrate potential. Inhibition profiles for major cytochrome P450 isoforms (CYP1A2, CYP2C19, CYP2C9, CYP2D6, and CYP3A4). The Bioavailability Radar was using to visualize the drug-likeness medicinal chemistry space ensuring the compounds' properties fell within the optimal range (represented by the pink area).

#### 2.4.2 Molecular docking study

##### 2.4.2.1. Preparation of ligands and protein structures

The three-dimensional (3D) structures of the compounds were constructed with the 2D-sketcher in Schrödinger (v2025-2; Schrödinger, Inc., New York), where their absolute configurations were assigned. The compounds were prepared using the Ligprep module by energy-minimizing with force OPLS4 force field, generating possible states with Epik and maintaining the assigned chirality from the 3D structure. The crystal structure of COX-1 and COX-2 enzymes were retrieved from the RCSB Protein Data Bank (PDB: 3KK6 and 6COX,^33^ respectively) in complex with celecoxib and 1-phenylsulfonamide-3-trifluoromethyl-5-parabromophenylpyrazole, respectively. Chain A of 6COX and its corresponding co-crystallized ligand were retained, and the rest were deleted. The protein was subsequently prepared using the protein preparation wizard module, where hydrogen atoms were added and optimized, missing side chains and loops were filled, N- and C- terminals were capped with *N*-acetyl and *N*-methylamide groups, respectively, and eventually, energy-minimized using the OPLS4 force field.

##### 2.4.2.2. Induced-fit docking

To allow the flexibility of the sidechain residues in the binding pocket of 6COX, an Induced-Fit Docking (IFD) was conducted using the IFD module as reported in the literature. The centroid of the co-crystallized ligand was selected as the centre of the Glide grid, and ligands with similar sizes to the co-crystallized ligand were allowed to dock. Ligands were initially docked into the receptor by applying a scaling factor of 0.5 to both ligand and protein van der Waals radius. The standard protocol was applied to generate 20 preliminary poses per ligand; nonplanar amide bonds were penalized, and planarity of conjugated pi groups was enhanced. The side chains of residues within 5 Å of the ligand and the docked ligands were refined with Prime. Subsequently, a a standard precision (SP) Glide redocking of each protein–ligand complex structure within 30 kcal mol^−1^ of the lowest energy structure was performed, generating up to 5 poses per ligand.^34^

##### 2.4.2.3. Rescoring with molecular mechanics, general born surface area (MM/GBSA)

The best poses obtained were further rescored with MM-GBSA free binding energy calculations using the Prime MM-GBSA module with default parameters. The VSGB implicit solvation model and residues within 5 Å of the co-crystallized ligands were included in the flexible region. The OPLS4 Force Field and minimized sampling method were used to calculate the binding energies. To this end, the binding energies were evaluated, and the docked poses were visually inspected to select the best pose.

##### 2.4.2.4. Molecular dynamics simulations

The molecular dynamics simulation was conducted utilizing the Schrödinger (v2025-2; Schrödinger, Inc., New York). Using the System Builder, the selected docked pose was placed at the center of an orthorhombic box with periodic boundary conditions (minimum distance of 10 Å between protein and boundary). Afterwards, the system was solvated with TIP5P and neutralized with NaCl (0.15 M). The system was energy minimized with OPLS4 while restraining the protein and ligand atoms. After minimization, the system was simulated for 100 ns without any restraints at a constant temperature (300 K, Nose–Hoover Chain, default settings) and pressure (1 bar, Martyna-Tobias-Klein, default values), using the RESPA integrator with a time step of 0.002 fs. Analysis of the trajectory was conducted using the Simulation Interaction Diagram (SID) module in Schrödinger (v2025-2; Schrödinger, Inc., New York).^35^

#### 2.4.3. DFT studies

The computational investigation of the selected active compounds was carried out using the Gaussian 09W software suite, with molecular structures visualized through Gauss View 5.0. The electronic properties of compounds 3a, 3e, and 3j were examined by applying density functional theory (DFT) calculations at the B3LYP/6-31G(d,p) level, following methodologies consistent with previously reported studies.^36,37^

## 3. Results and discussion

### 3.1 Chemistry

**Scheme 1:**
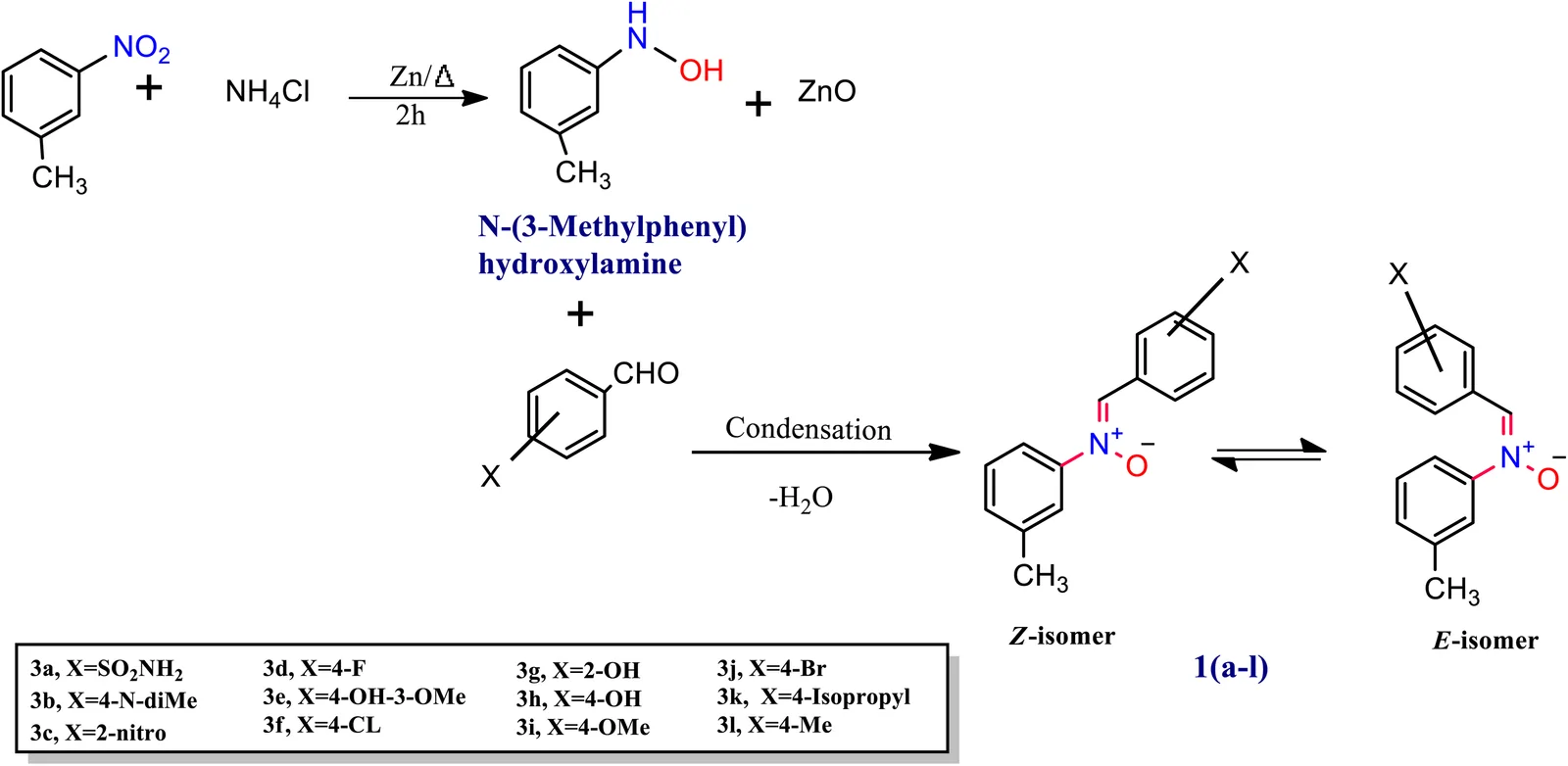
Synthesis of nitrones (1a–l).

**Scheme 2:**
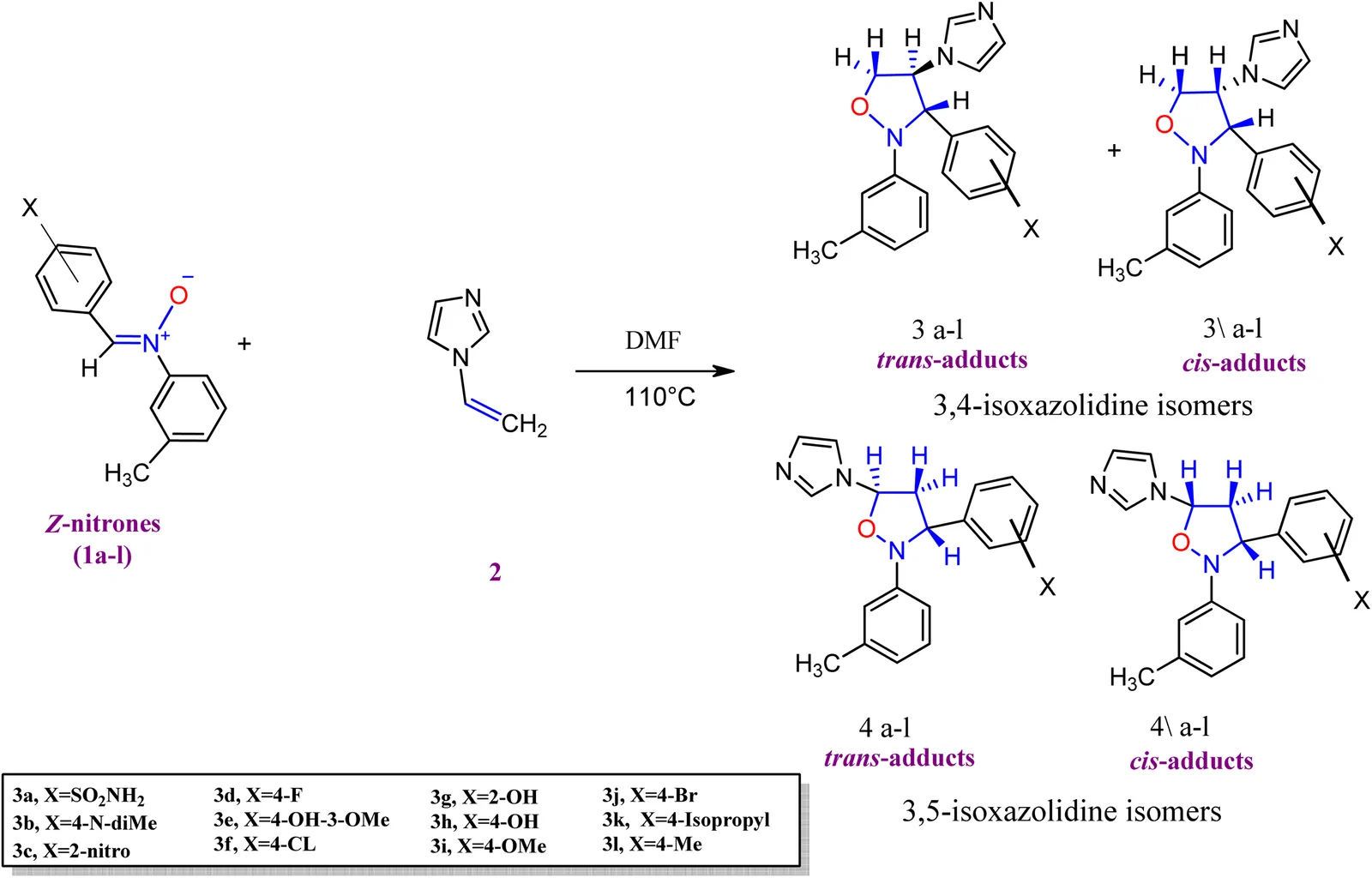
Synthesis of isoxazolidine derivatives (3a–l).

The target nitrones (1a–l) were synthesized through the following two-step procedure. The target nitrones (1a–l) were synthesized through the following two-step procedure. In the first step, *N*-(3-methylphenyl)hydroxylamine was prepared by the selective reduction of 3-nitrotoluene using zinc dust and ammonium chloride (Zn/NH_4_Cl) as the reducing system in an aqueous ethanolic medium. After completion of the reaction, the mixture was filtered, and the product was isolated to afford *N*-(3-methylphenyl)hydroxylamine as light yellow needle-like crystals. In the second step, nitrones (1a–l) were synthesized by the condensation of *N*-(3-methylphenyl)hydroxylamine with the appropriate substituted aromatic aldehydes in absolute ethanol. The reaction mixture was stirred at room temperature in the dark overnight. Upon completion, the products were isolated by filtration and recrystallized from hot ethanol to afford the corresponding aryl nitrones in good to excellent yields, as shown in Scheme 1. The synthesized nitrones were subsequently subjected to regio- and diastereoselective 1,3-dipolar cycloaddition with equimolar amounts of 1-vinylimidazole in DMF under reflux at 110 °C for 15–30 h. The cycloaddition afforded the corresponding isoxazolidine derivatives (3a–l) as *cis* and *trans* cycloadducts in good to high yields, as outlined in Scheme 2.

The cycloaddition reactions proceeded with high regio- and diastereoselectivity, affording structurally well-defined isoxazolidine derivatives. The synthesized compounds were structurally characterized by FTIR, ^1^H NMR, ^13^C NMR, and LC-MS analyses. The compounds were subsequently evaluated for their COX-2 inhibitory activity and exhibited promising biological activity. In addition, *in silico* ADMET analyses were performed to assess their drug-likeness, oral bioavailability, and pharmacokinetic properties, revealing favorable pharmacokinetic profiles.

### 3.2 Spectral analysis

#### 3.2.1. FTIR ansalysis

The FTIR spectra of the synthesized isoxazolidine derivatives exhibited characteristic absorption bands at 1094–1010, 1177–1144, and 1373–1244 cm^−1^, corresponding to the C–N, N–O, and C–O stretching vibrations of the isoxazolidine ring, respectively. In addition, characteristic absorption bands observed at 1640–1680 cm^−1^ and around 1576 cm^−1^ were assigned to the C=N and C=C stretching vibrations of the imidazole ring, respectively.

#### 3.2.2. NMR analysis

**Scheme 3:**
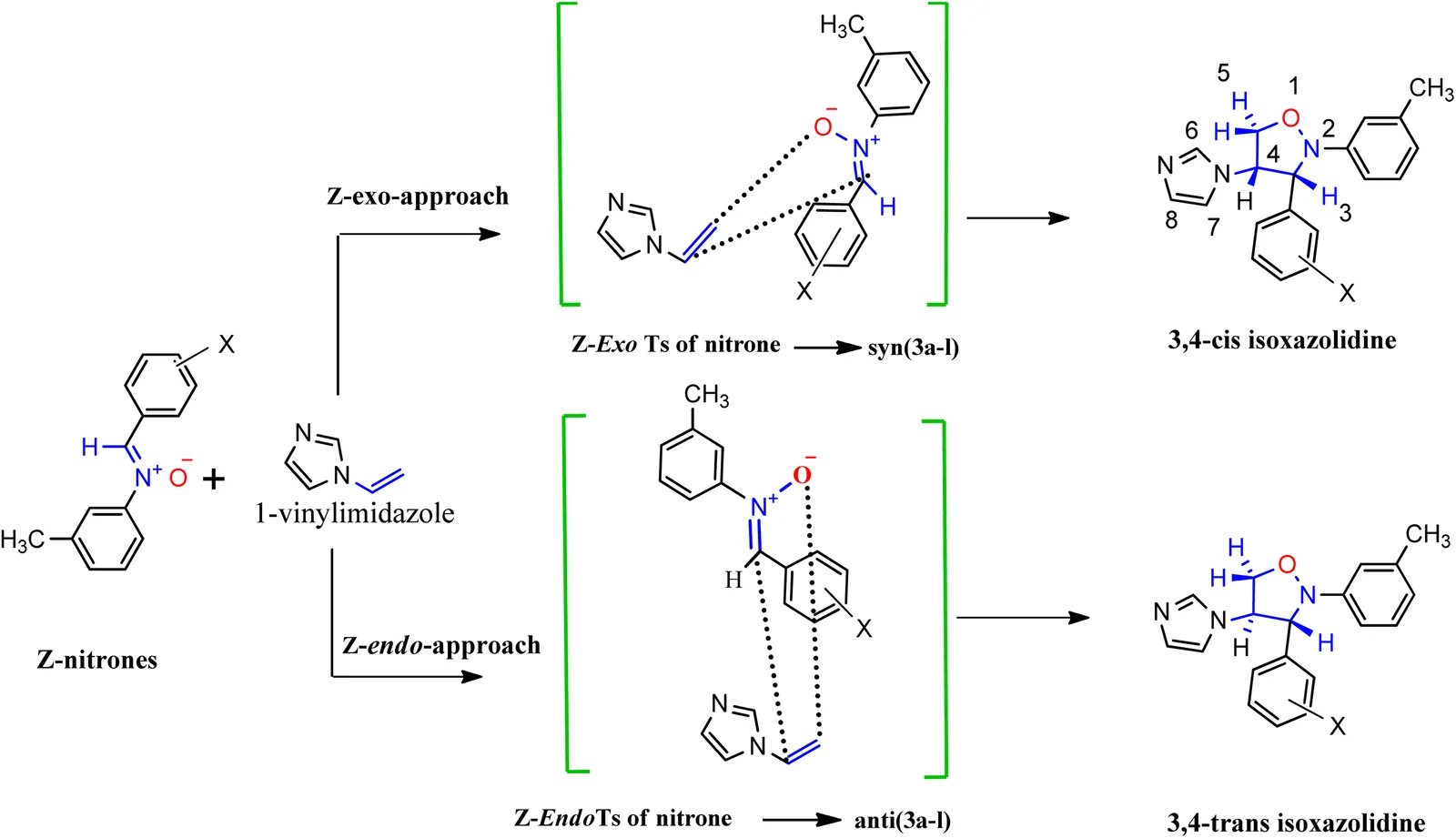
Two possible attacks to *Z*-nitrone.

The structures and relative stereochemistry of the synthesized isoxazolidine derivatives were established by FTIR, ^1^H NMR, ^13^C NMR, and LC-MS analyses. According to frontier molecular orbital (FMO) theory, monosubstituted alkenes bearing electron-donating substituents favor interaction between the LUMO of the nitrone and the HOMO of the dipolarophile, predominantly affording the 3,5-substituted isoxazolidine regioisomer. In the present study, 1-vinylimidazole, a monosubstituted alkene containing an electron-donating imidazole moiety, was employed as the dipolarophile. Based on previous studies, the reaction of a *Z*-configured nitrone with such dipolarophiles is expected to produce predominantly the 3,5-substituted isoxazolidine regioisomer. Furthermore, C,N-diaryl nitrones preferentially adopt the *Z* configuration, in which the aryl groups are oriented *trans* to one another, thereby minimizing steric hindrance during the cycloaddition process. Unexpectedly, the reaction afforded the 3,4-substituted isoxazolidine regioisomer as the exclusive product instead of the anticipated 3,5-regioisomer. This unusual regioselectivity may be attributed to steric effects, whereby the cycloaddition proceeds through the less sterically hindered transition state, minimizing repulsive interactions between the imidazole ring and the aryl substituent of the nitrone, as illustrated in Scheme 3. Regio and stereo-selectivity in the cycloaddition reactions is controlled by the structure of nitrones, alkenes, steric hindrance factors, and other factors.^38^

**Fig. 2:**
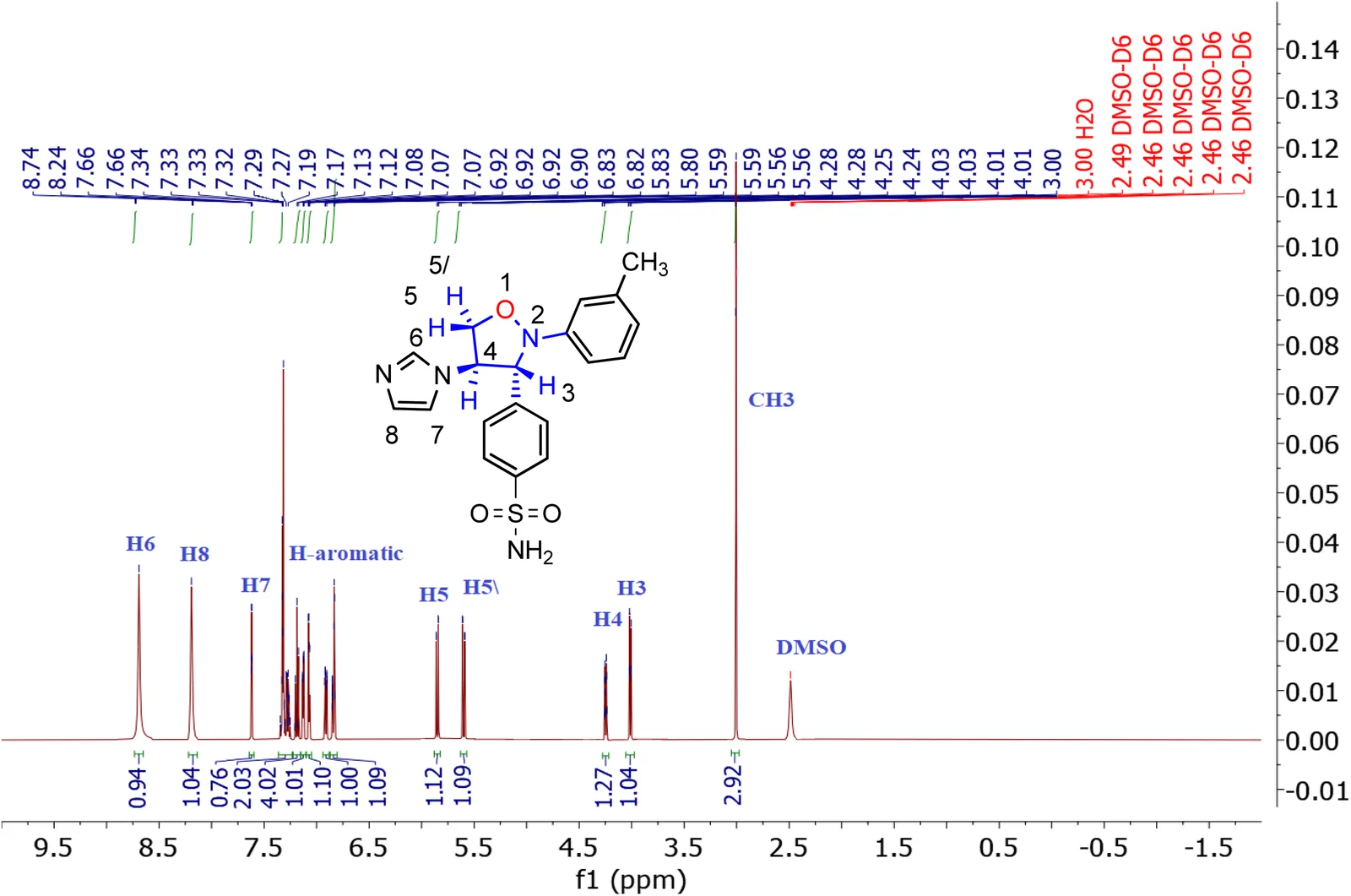
^1^H-NMR of isoxazolidine (3a).

**Fig. 3:**
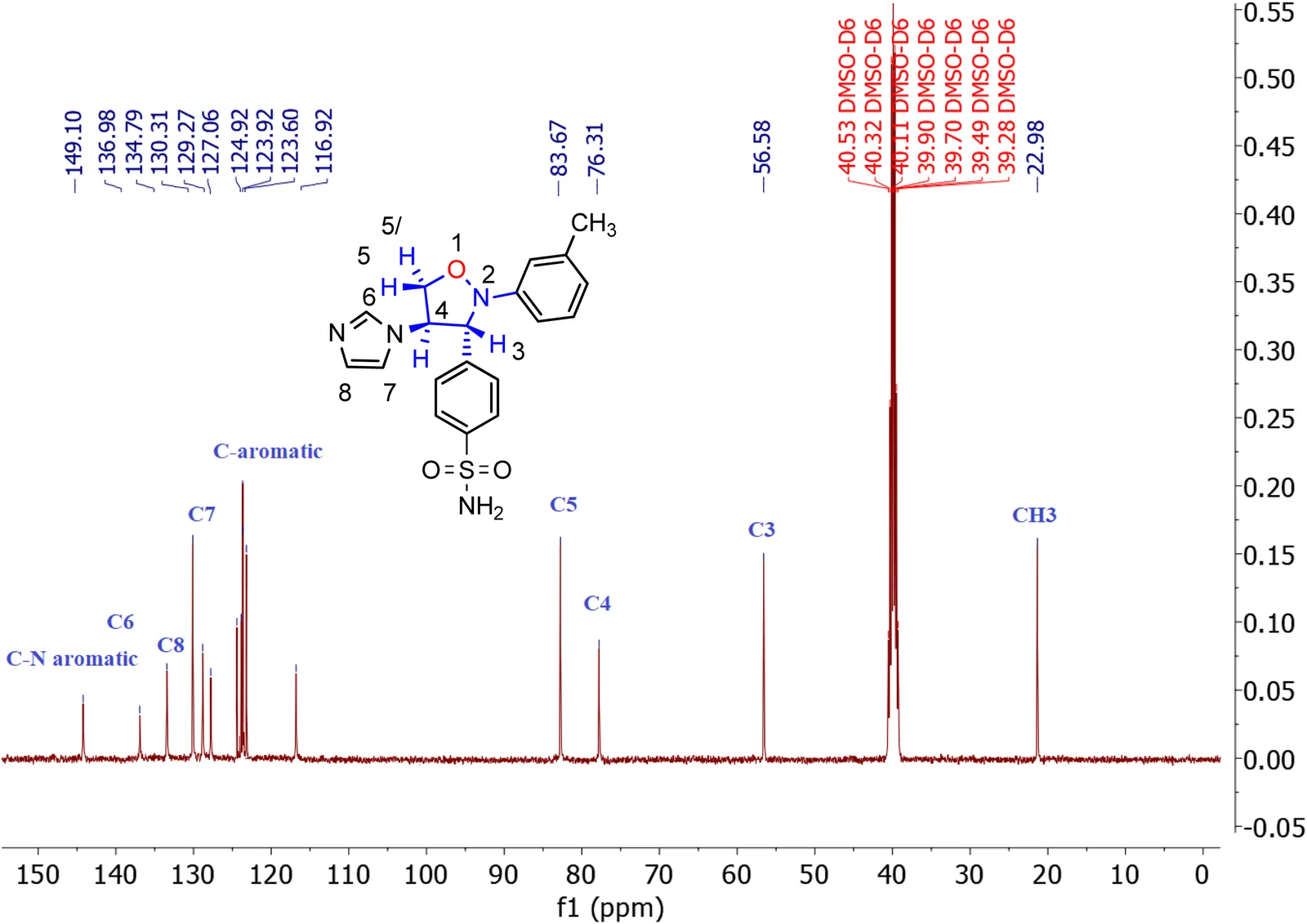
^13^C-NMR Spectrum for isoxazolidine (3a).

The stereoselectivity for this reaction was carried out through the endo-transition state between the *Z*-nitrone and 1-vinylimidazole based on the ^1^H NMR spectra analysis. Compound 3a was selected as the representative model for stereochemical assignment. The ^1^H-NMR spectrum, of compound 3a showed two (dd) at high chemical shift in the range *δ* = 5.83 ppm for H5 as doublet of doublet with a coupling constant of (*J*_H5, H5\_ = 10.8, *J*_H5, H4_ = 8.8Hz). A large coupling constant value *J*_H5, H4_ = 8.8 Hz between H5, H4 indicates their relationship is *trans*. While another methylene proton H5\ observed at *δ* = 5.56 ppm as doublet of doublet with a coupling constant of (*J*_H5\, H5_ = 10.9), *J*_H5, H4_ = 4.3Hz). A small coupling constant value between H5\, H4 indicates their relationship is *cis*. The presence of diastereotopic methylene protons H5, H5\ in isoxazolidine ring in ^1^H-NMR spectrum at a high chemical shift as in Fig. 2 is strong evidence on formation regioisomers 3,4-isoxazolidine compared to a previous study literature for related compounds^39^ and effectively excludes the formation of the another 3,5-isoxazolidine regioisomer. H4 observed as doublet of triplet at *δ* = 4.28–4.24 ppm with a coupling constant of (*J*_H4, H3_ = 10.5, *J*_H3, H5, H5\_ = 1.7 Hz). on the other side, the proton H3 observed as doublet of triplet at *δ* = 4.03–4.01 ppm with a coupling constant of (*J*_H3, H4_ = 10.8, *J*_H3, H7, H8_ = 1.8Hz) from large coupling constant value between H3 and H4 indicates the relationship between them is *trans*. The ^13^C-NMR spectra of compound (3a) displayed peaks at *δ* = 83.7, 76.3 and 56.5 ppm characterize of C5, C4, and C3 in the isoxazolidine ring, respectively. The chemical shift values of three peaks have been found at *δ* = 136.9, 134.7 ppm, and 130.3 ppm the characteristic peak of C6, C8, C7, respectively in imidazole ring as in Fig. 3.

### 3.3. Biological evaluation

#### 3.3.1. Enzyme inhibitory activity

The inhibitory activities of the newly synthesized isoxazolidine derivatives against COX-1 and COX-2 are summarized in Table 1 as pIC_50_ and IC_50_ values. The tested compounds (3a–l) showed a variety of inhibition profiles against both cyclooxygenase isoforms, which made it possible to evaluate their potency and selectivity. Compound 3a, bearing a sulfonamide substituent (–SO_2_NH_2_), exhibited the highest COX-2 inhibitory potency with a COX-2 pIC_50_ value of 6.74 ± 0.05, which is markedly higher than that of the reference drug celecoxib (pIC_50_ = 6.04 ± 0.02). In contrast, compound 3a showed a much lower COX-1 inhibitory potency (COX-1 pIC_50_ = 4.19 ± 0.02), comparable to that of celecoxib (4.17 ± 0.02). Consequently, compound 3a displayed the highest selectivity index (SI = 361.1), which was approximately 4.8-fold greater than that of celecoxib (SI = 74.9), identifying it as the most COX-2-selective derivative in the series. Whereas the majority of the remaining compounds showed weak to moderate inhibition of COX-1, with pIC_50_ values ranging from 4.24 ± 0.02 to 4.43 ± 0.03, suggesting that these compounds have a favorable safety margin associated with poor suppression of COX-1. Among these derivatives, compound 3e also demonstrated excellent COX-2 inhibitory potency (pIC_50_ = 6.60 ± 0.05), exceeding that of celecoxib (6.04 ± 0.02), while maintaining weak COX-1 inhibition (pIC_50_ = 4.24 ± 0.02). This resulted in a selectivity index of 232.0, approximately 3.1-fold higher than that of celecoxib. On the same note, compound 3j that had a *para*-bromo substituent gave good COX-2 inhibitory activity (pIC_50_ of 6.52 ± 0.04) and weak COX-1 activity (pIC_50_ = 4.28 ± 0.02), resulting in a high selectivity index (SI = 173.3), approximately 2.3-fold greater than that of the reference drug. These findings indicate that bulky *para* substitution with halogen has a major role in increasing the COX-2 selectivity due to favorable steric interaction in the larger COX-2 active site, both electron-withdrawing and electron-donating substituents have significant effects on COX-2 binding affinity, but their effects on isoform selectivity are weaker. Such behavior can be explained by the fact that suboptimal electronics or steric properties restrict selective accommodation in the COX-2 binding pocket. All in all, these data underscore the importance of substituent type and substitution position in regulating COX-2 activities and selectivity. Specifically, three of these compounds (compound 3a, 3e and 3j) were most promising COX-2-preferential inhibitors in this series, that is, with potent COX-2 inhibition and low COX-1 activity, but they are attractive lead compounds with respect to further structural optimization and subsequent biological testing.

**Table 1:** Inhibition of COX-1, COX-2 by the newly synthesized isoxazolidine derivatives

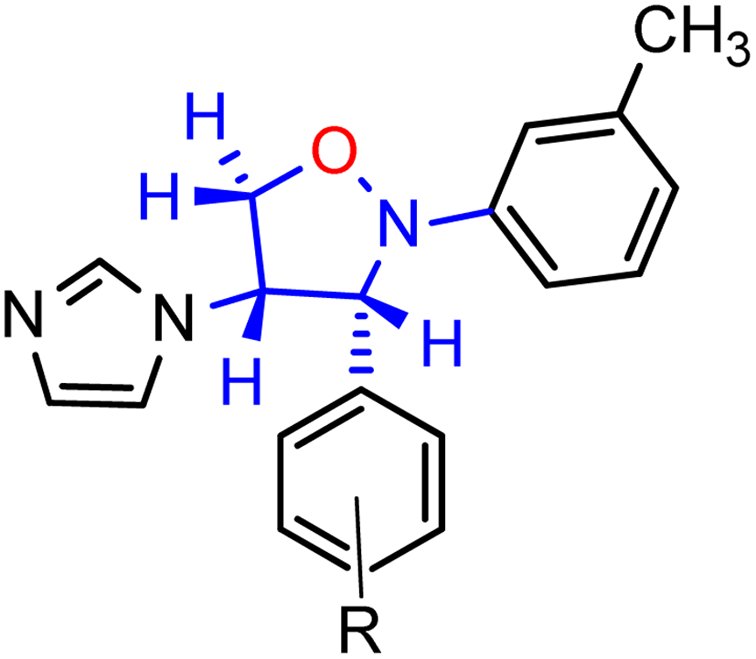						
Compounds	*R*	apIC_50_ ± SD		bIC_50_ ± SD (µM)		Selectivity index c(SI)
		COX-1	COX-2	COX-1	COX-2	
3a	**4-SO**_**2**_ **NH2**	**4.19 ± 0.02**	**6.74 ± 0.05**	**65.0 ± 3.5**	**0.18 ± 0.02**	**361.1**
3b	-4N-diMe	4.40 ± 0.03	6.07 ± 0.04	40.0 ± 2.5	0.85 ± 0.07	47.1
3c	2-Nitro	4.38 ± 0.03	6.10 ± 0.03	42.0 ± 2.8	0.80 ± 0.06	52.5
3d	4-F	4.39 ± 0.03	6.05 ± 0.04	41.0 ± 2.6	0.90 ± 0.08	45.6
3e	**4-OH-3-OMe**	**4.24** ± **0.02**	**6.60** ± **0.05**	**58.0** ± **3.0**	**0.25** ± **0.03**	**232.0**
3f	4-CL	4.37 ± 0.03	6.12 ± 0.03	43.0 ± 2.7	0.75 ± 0.06	57.3
3g	2-OH	4.42 ± 0.03	5.60 ± 0.03	38.0 ± 2.2	2.50 ± 0.20	15.2
3h	4-OH	4.41 ± 0.03	5.42 ± 0.03	39.0 ± 2.3	3.80 ± 0.30	10.3
3i	4-OMe	4.40 ± 0.03	5.54 ± 0.04	40.0 ± 2.4	2.90 ± 0.25	13.8
3j	**4-Br**	**4.28** ± **0.02**	**6.52** ± **0.04**	**52.0** ± **2.9**	**0.30** ± **0.03**	**173.3**
3k	4-Isopropyl	4.44 ± 0.03	5.35 ± 0.03	36.0 ± 2.1	4.50 ± 0.3	8.0
3l	4-Me	4.43 ± 0.03	5.51 ± 0.04	37.0 ± 2.2	3.10 ± 0.28	11.9
Celecoxib	—	4.53 ± 0.03	6.42 ± 0.09	29.2 ± 1.8	0.39 ± 0.08	74.9

a pIC_50_ values represent the negative logarithm of the IC_50_ values (M) and are expressed as the mean ± SD of three independent determinations.

b The concentration of test compounds required to produce 50% inhibition of COX-1/COX-2 is the mean ± SD of three determinations.

c SI= (COX-1_IC_50__/COX-2_IC_50__)

#### 3.3.2. Structure–activity relationship (SAR) analysis

A thorough SAR analysis was performed to rationalize the influence of aromatic substituents on the COX-1 and COX-2 inhibitory potency and isoform selectivity of the synthesized isoxazolidine derivatives 3a–l. All compounds share a common isoxazolidine scaffold bearing an imidazolyl group and a 3-methylphenyl moiety on nitrogen, with structural variation confined to the *para*- or *ortho*-substituted pendant phenyl ring, thereby allowing a systematic evaluation of electronic and steric effects on biological activity. The sulfonamide-containing derivative 3a exhibited the highest COX-2 inhibitory potency (pIC_50_ = 6.74 ± 0.05) and selectivity (SI = 361.1) surpassing the reference drug celecoxib (SI = 74.9) by nearly five-fold. The exceptional activity of this compound may be attributed to the sulfonamide pharmacophore, which is well recognized as a key structural feature in selective COX-2 inhibitors. The presence of the SO_2_NH_2_ group provides additional hydrogen-bond donor and acceptor sites that can interact favorably with residues located within the COX-2 secondary binding pocket, thereby enhancing both potency and selectivity. Among the oxygen-bearing derivatives, compound 3e bearing hydroxyl and methoxy substituents showed excellent COX-2 inhibitory potency (pIC_50_ = 6.60 ± 0.05, SI = 232.0). The superior performance of this compound compared with the mono-substituted analogues 3g (2-OH), 3h (4-OH), and 3i (4-OMe) suggests that the simultaneous presence of both hydroxyl and methoxy groups provides a synergistic effect. These substituents may improve binding through a combination of hydrogen-bonding interactions and favorable electronic donation to the aromatic ring. The halogen-substituted derivatives revealed a clear substituent-size effect. Compound 3j containing a *para*-bromo group displayed markedly stronger COX-2 inhibition (pIC_50_ = 6.52 ± 0.04) than the fluoro (3d) and chloro (3f) analogues. The enhanced activity of 3j may result from the larger size and greater polarizability of bromine, allowing stronger hydrophobic interactions and better occupation of the enlarged COX-2 binding cavity. These findings suggest that steric factors play an important role in optimizing ligand accommodation within the enzyme active site. The *para*-fluorine derivative 3d (*R* = 4-F; pIC_50_ = 6.05 ± 0.04; SI = 45.6) showed the weakest activity among the halogenated compounds, consistent with fluorine's minimal steric contribution despite its strong electronegativity, suggesting that steric bulk rather than electron withdrawal governs halogen-dependent COX-2 selectivity in this scaffold. Regarding nitrogen-containing substituents, the *para*-dimethylamino derivative 3b (*R* = 4-NMe_2_; pIC_50_ = 6.07 ± 0.04; SI = 47.1) and the *ortho*-nitro analogue 3c (*R* = 2-NO_2_; pIC_50_ = 6.10 ± 0.04; SI = 52.5) exhibited moderate COX-2 potency with limited selectivity. While the electron-donating dimethylamino group may engage in weak van der Waals interactions, its bulky geometry at the *para*-position does not appear to confer the directional interactions required for high selectivity. The *ortho*-nitro group in 3c, despite its strong electron-withdrawing character, likely introduces steric strain that prevents optimal docking geometry within the COX-2 binding pocket. Alkyl-substituted derivatives 3k (*R* = 4-isopropyl; pIC_50_ = 5.35 ± 0.03; SI = 8.0) and 3l (*R* = 4-Me; pIC_50_ = 5.51 ± 0.03; SI = 11.9) were the least active compounds in the series. The absence of hydrogen-bonding capability combined with purely hydrophobic character renders these substituents unable to establish the specific polar interactions necessary for potent COX-2 engagement, while the branched isopropyl group of 3k may additionally impose steric penalties that further reduce binding affinity. The SAR analysis revealed a distinct hierarchy in the contribution of different substituents to COX-2 inhibitory potency and isoform selectivity within this isoxazolidine scaffold. The observed activity trend was ranked as follows: sulfonamide (3a) >> *para*-hydroxyl, *meta* methoxy (3e) > *para*-bromo (3j) > *para*-chloro (3f) > nitro (3c) ≈ dimethylamino (3b) > fluoro (3d) > *ortho*-hydroxyl > *para*-methoxy > *para*-hydroxyl >> *para*-alkyl. These findings demonstrate that enhanced COX-2 selectivity is strongly influenced by the presence of substituents capable of establishing favorable interactions with the secondary hydrophilic pocket of the COX-2 active site, particularly through hydrogen-bonding interactions, as observed with the sulfonamide groups (SO_2_NH_2_, OH/OMe combination). Furthermore, the incorporation of additional steric bulk at the *para*-position, as exemplified by the bromine substituent, was found to improve both inhibitory potency and isoform selectivity. Collectively, these results identify compounds 3a, 3e, and 3j as promising lead candidates for further structural optimization toward the development of potent and selective COX-2 inhibitors.

### 3.4. ADMET results

**Fig. 4:**
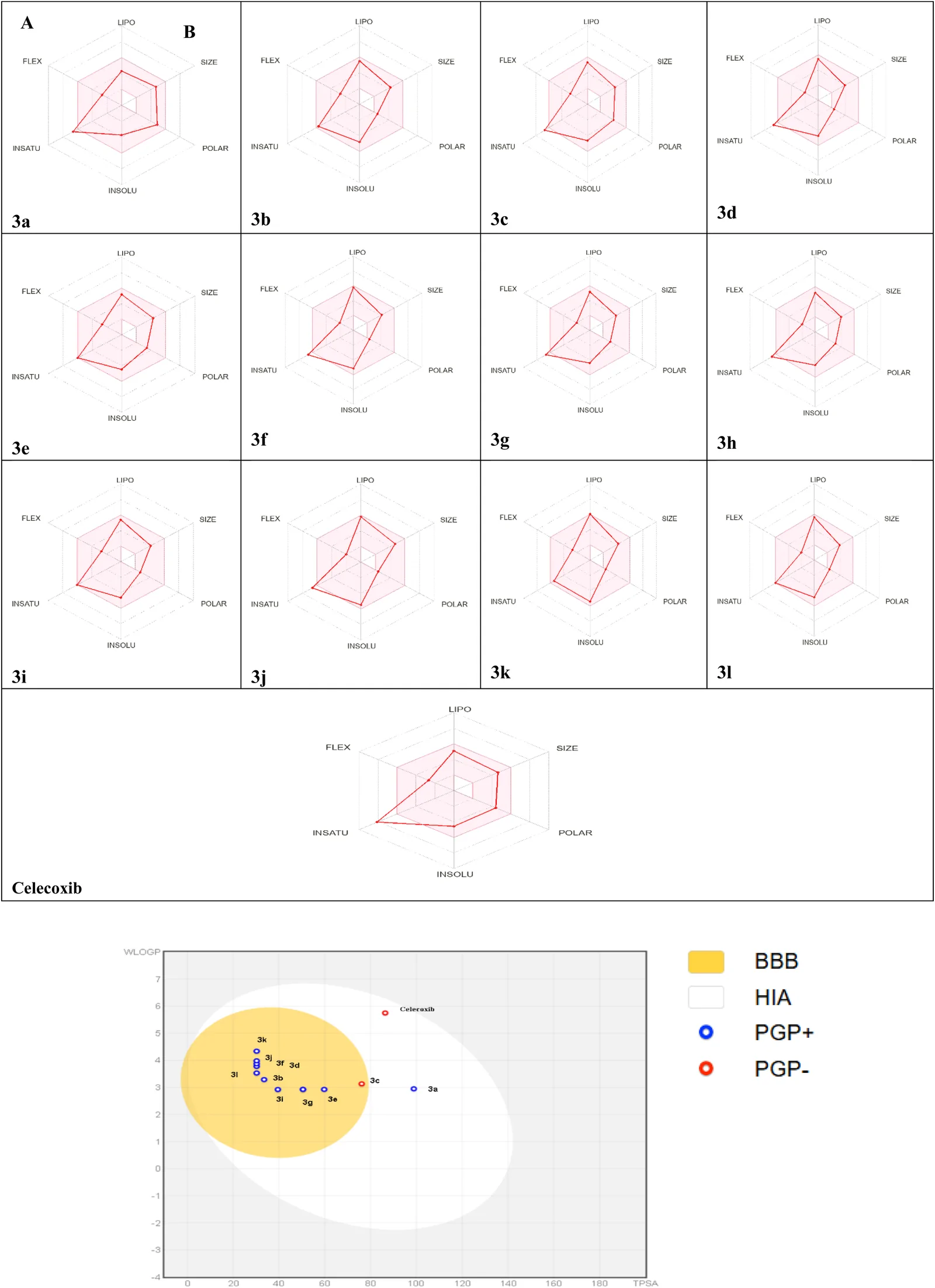
(A) Bioavailability radar; (B) the boiled-egg graph of compounds (3a–l) the yellow ellipse defining brain permeation and the white ellipse defining intestinal absorption.

All compounds (3a–l) demonstrate excellent adherence to Lipinski's Rule of Five, indicating their suitability as potential orally active drugs.^40–45^ Molecular Weight (MW), all compounds are well below the 500 g mol^−1^ threshold (in rang from 319 to 384 g mol^−1^), suggesting they are small enough to predicted pass through cell membranes easily. Lipophilicity (XLOGP3), most compounds fall within the ideal range (2.08 to 5.09). High lipophilicity as in 3k at 5.09 often correlates with better membrane permeability but may require monitoring for water solubility. Hydrogen bonding, the values for HBD (≤1) and HBA (2–5) are significantly below the limits (<5 and <10, respectively), which favors passive diffusion, the TPSA values range mostly between 30 and 98.86 Å2 excluding 3a, TPSA <140 Å2 is good for cell permeability, and <60 Å2 is excellent for brain penetration, as summarized in Table 2. Gastrointestinal absorption (GIA): all the synthesized compounds are predicted to exhibit high GIA, which implies that they will tend to be absorbed successfully in the digestive tract. For the predicted percentage absorption (%ABS), the majority of the tasted compounds were very high with the absorption score of 98.86, was much higher than that predicted for the reference compound, Celecoxib (62.82%). Blood–Brain Barrier (BBB) Permeability, all compounds are predicted to pass the BBB except the therapeutic target compound 3a, which is a critical factor whether the therapeutic target is in the Central Nervous System (CNS). Metabolism & CYP450 Inhibition: The majority of compounds are predicted to inhibit CYP2C19 and CYP2C9, thus may decelerate the metabolism of other drugs. Nevertheless, some (3d, 3e, 3f, 3g, 3h, 3j) do not interact with CYP3A4, which means that there are fewer risks of drug–drug interactions. Efflux (P-gp): although the majority of them are predicted to be P-gp substrates, 3c is predicted not to be a substrate, indicating that is less prone to pumping out of cells (improved cellular retention). Predicted synthetic accessibility (SA): it has a score of 3.85 to 4.19. These compounds have been ranked in a scale of 1(very easy) and 10 (very difficult) to make in a lab. Moreover, the bioavailability radar analysis showed that all compounds fall within the optimal physicochemical space, indicating superior oral bioavailability and supporting their potential as promising drug candidates (Fig. 4A). The predicted permeability of the compounds through biological membranes is illustrated by the BOILED-Egg diagram (Fig. 4B), in which compounds located within the yolk region are predicted to be blood–brain barrier (BBB) permeable, while compounds located within the white region only are predicted to show human intestinal absorption (HIA) without BBB penetration. Blue and red markers represent compounds predicted to be P-glycoprotein substrates (PGP+) and non-substrates (PGP−), respectively. As shown in Fig. 4B, most compounds are predicted to be P-gp substrates (PGP+, blue markers) and are therefore expected to undergo active efflux, whereas 3c is predicted to be a P-gp non-substrate (PGP−, red marker), suggesting reduced susceptibility to efflux and potentially improved cellular retention. In generally, the tested series (3a–l) demonstrates better predicted pharmacokinetic profiles than the reference compound (Celecoxib), especially, in terms of intestinal absorption and compliance with drug-likeness principles. Such compounds can therefore be considered strong candidates for future *in vitro* and *in vivo* research.

**Table 2:** Physicochemical-pharmacokinetic/ADME properties and drug-likeness predictions of tested compounds 3a–l^a^

Entry	3a	3b	3c	3d	3e	3f	3g	3h	3i	3j	3k	3l	S
Pharmacokinetics													
MW (<500 Da)	384.45 g mol^−1^	348.44 g mol^−1^	350.37 g mol^−1^	323.36 g mol^−1^	351.40 g mol^−1^	339.82 g mol^−1^	321.37 g mol^−1^	321.28 g mol^−1^	335.40 g mol^−1^	384.27 g mol^−1^	347.45 g mol^−1^	319.40 g mol^−1^	381.37 g mol^−1^
Rb	4	4	4	3	4	3	3	3	4	3	4	3	4
HBA (<10)	5	2	4	3	4	2	3	3	3	2	2	2	7
HBD (<5)	1	0	0	0	1	0	1	1	0	0	0	0	1
TPSA(Å^2^)	98.8	33.5	76.11	30.2	59.7	30.2	50.5	50.5	39.5	30.2	30.2	30.2	86.3
ABS%	98.8	97.7	82.54	98.8	89.0	98.8	92.1	92.1	95.7	98.8	98.8	98.8	62.8
Fraction Csp3	0.21	0.29	0.21	0.21	0.25	0.21	0.21	0.21	0.25	0.21	0.32	0.25	0.12
XLOGP3 (Log *P* < 5)	2.08	3.64	3.80	4.07	3.58	4.60	3.16	3.16	3.94	4.66	5.09	4.33	−3.40
GIA	High	High	High	High	High	High	High	High	High	High	High	High	High
BBBP	No	Yes	Yes	Yes	Yes	Yes	Yes	Yes	Yes	Yes	Yes	Yes	No
PgPS	Yes	Yes	No	Yes	Yes	Yes	Yes	Yes	Yes	Yes	Yes	Yes	No
CYP1A2 INHIBITION	Yes	Yes	No	Yes	Yes	Yes	No	No	Yes	Yes	Yes	Yes	Yes
CYP2C19 INHIBITION	Yes	Yes	Yes	Yes	Yes	Yes	Yes	Yes	Yes	Yes	Yes	Yes	No
CYP2C9 INHIBITION	No	Yes	Yes	Yes	Yes	Yes	Yes	Yes	Yes	Yes	Yes	Yes	Yes
CYP2D6 INHIBITION	No	Yes	No	No	Yes	No	Yes	Yes	No	No	No	No	No
CYP3A4 INHIBITION	Yes	Yes	Yes	No	No	No	No	No	Yes	No	Yes	Yes	No
Log *K*_p_ (cm s^−1^)	−7.17	−5.52	−5.74	−5.38	−5.90	−5.11	−5.70	−5.70	−5.55	−5.34	−4.81	−5.17	−6.21
LV	0	0	0	0	0	0	0	0	0	0	0	0	0
BS	0.55	0.55	0.55	0.55	0.55	0.55	0.55	0.55	0.55	0.55	0.55	0.55	0.55
LLV	1	1	2	1	2	1	1	1	1	2	1	1	1
SA	4.04	4.16	4.13	3.88	4.02	3.86	3.90	3.85	3.92	3.91	4.19	3.97	2.74

a S* = Celecoxib.

### 3.5. Molecular docking studies

**Fig. 5:**
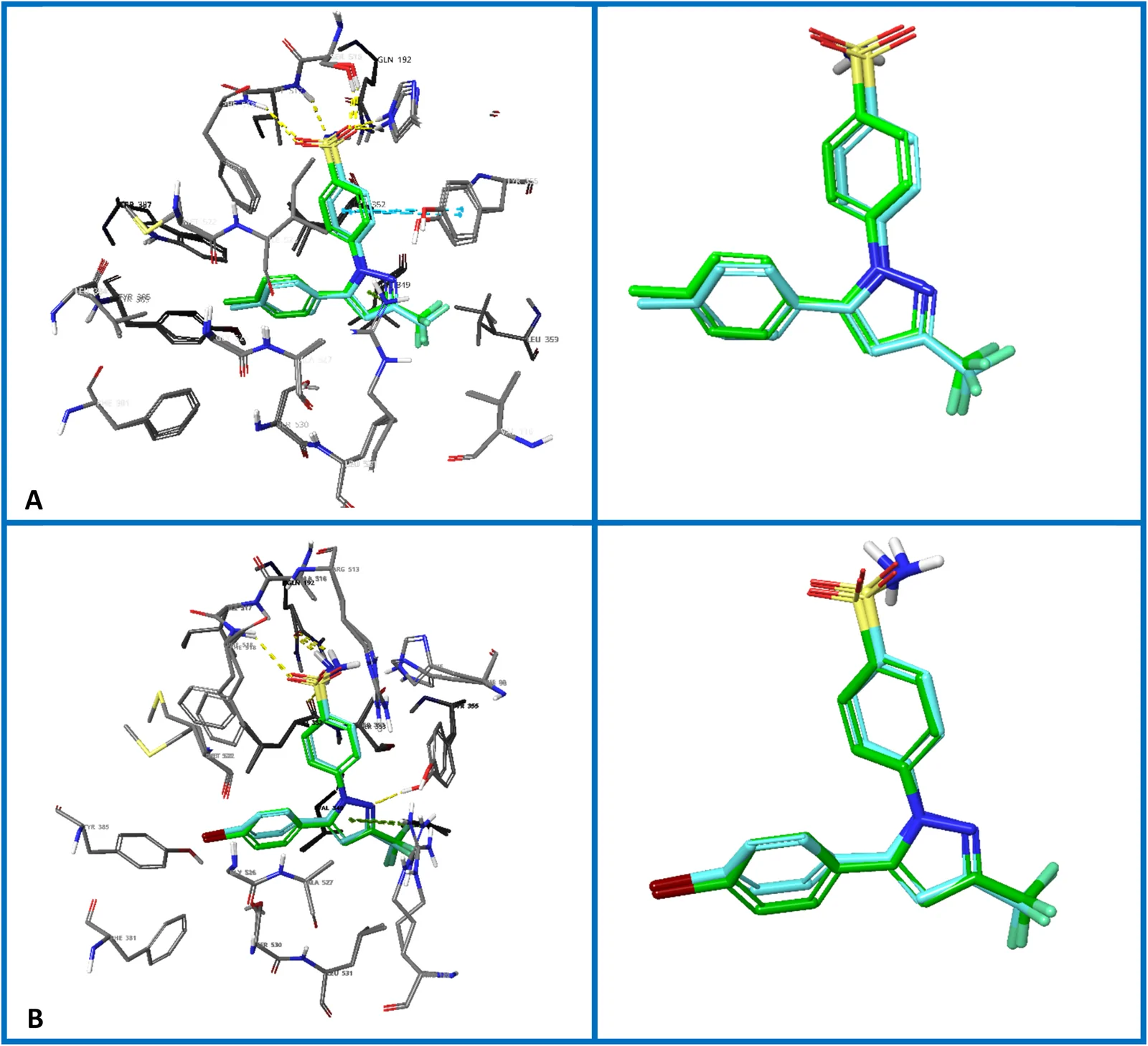
Superimposition of the co-crystallized (A) celecoxib (Turquoise) and the redocked pose of celecoxib (green) of COX-1 (PDB: 3KK6) (B) SC 558 (Turquoise) and the redocked pose of SC 558 (green) of COX-2 (PDB: 6COX).

Molecular docking studies were performed to evaluate the binding affinity and interaction profiles of the most active compounds (3a, 3e, and 3j) alongside the reference compound celecoxib for the COX-1 (PDB: 3KK6) and COX-2 (PDB: 6COX) enzymes. IFD was applied to address the flexibility limitation with normal docking. Subsequently, the IFD complexes were further re-scored using MM-GBSA binding free energy calculations. Eventually, the poses were subjected to a 100 ns MD simulation to assess their stability. Before docking the compounds, the applied IFD protocol was validated by assessing its pose-generation quality using the cocrystallized ligands (celecoxib and SC-558). The redocking results show a very similar orientation to the original cocrystallized poses, with RMSD values of 0.4401 and 0.5426 Å for COX-1 (PDB: 3KK6) and COX-2 (PDB: 6COX), respectively (Fig. 5). COX-1 and COX-2 are closely related because both have hydrophobic pockets, responsible for catalysis, and comprise Tyr385, Gly526, Trp387, Phe518, Leu384, and Ser530. Additionally, both have entrance regions responsible for substrate fixation, comprising Arg120 and Tyr355. However, certain structural differences in the active binding sites are exploited to develop selective COX-2 inhibitors.

**Fig. 6:**
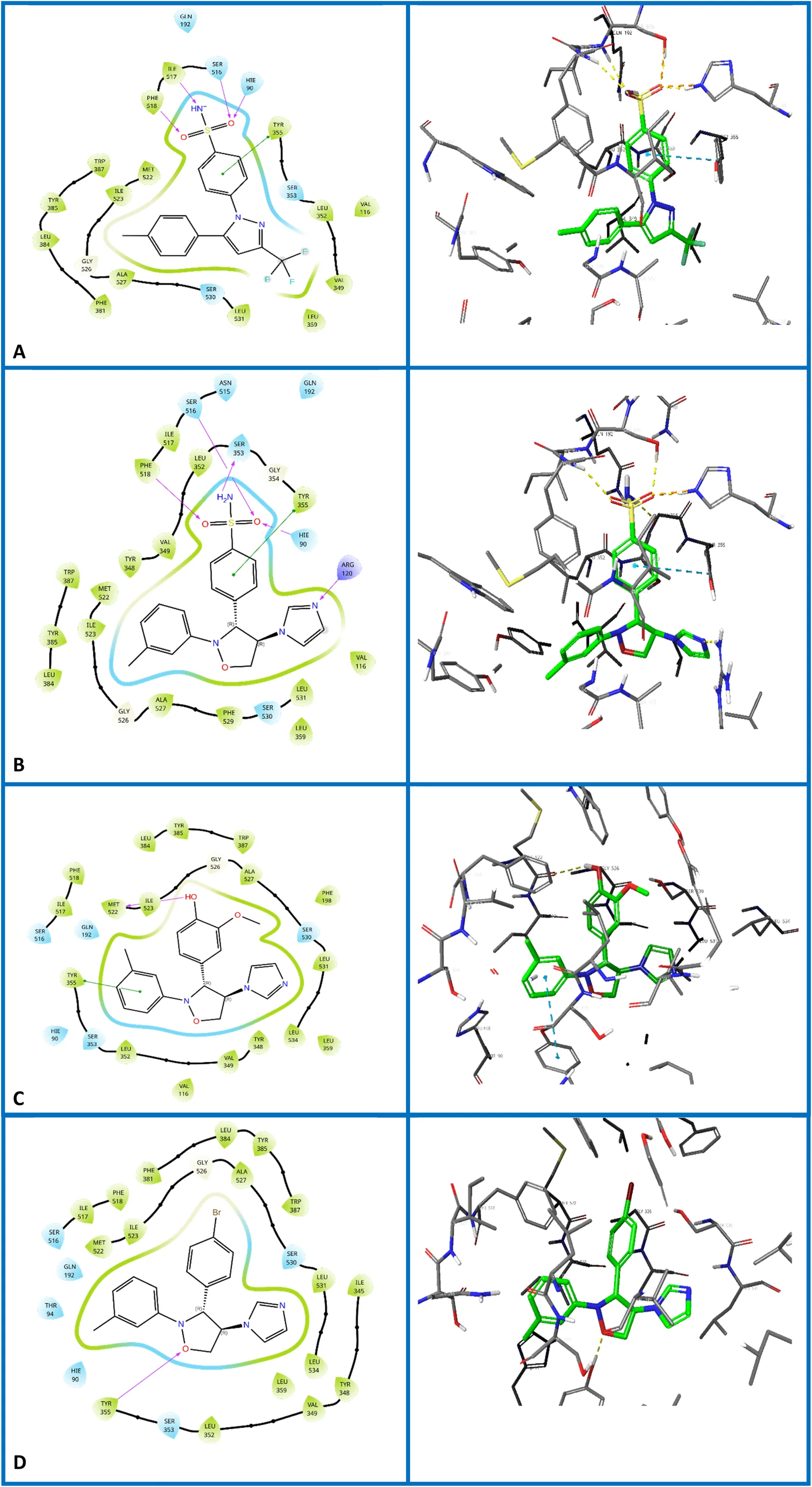
IFD docking poses of (A) celecoxib, (B) compound 3a, (C) compound 3e (D) compound 3j in COX-1 (PDB: 3KK6).

For example, Ile523 and His513 in COX-1 are replaced with Valine and Arginine in COX-2, respectively, resulting in a larger and more flexible cavity and an additional side pocket in COX-2, which comprises His90, Arg513, Gln192, and Ser353. Selective COX-2 inhibitors like the reference celecoxib extend their sulfonamide moiety to this additional pocket (Fig. 5 and 6A), which is restricted in COX-1 and also unoccupied by non-selective inhibitors.^46–49^

However, as reported in the X-ray crystal studies of celecoxib and COX-1 (PDB: 3KK6)^50^ Ile523 in COX-1 can adopt a different rotamer conformation that allows celecoxib to bind weakly, without well accommodating the sulfonamide group as in COX-2, disrupting interactions with key residues such as Gln192, forming a steric clash with Ser516 and His90, and contributing to an energetic penalty. Nevertheless, the docked pose provided in Fig. 6A shows that celecoxib forms hydrogen bonds with Phe518, Ile517, Ser516, and His90. In addition, the benzenesulfonamide forms a π–π interaction with Tyr355 and multiple hydrophobic contacts with the nearby hydrophobic residues. Similarly, the docked pose in Fig. 6B shows that compound 3a adopts a similar orientation to that of celecoxib and forms similar interactions, as well as a clash with His90. Additionally, the imidazole formed a hydrogen bond with Arg120. In Fig. 6C, the phenolic OH of compound 3e forms a hydrogen bond with Met522, whereas the 3-methylphenyl forms a π–π interaction with Tyr355. The imidazole ring forms hydrophobic contacts with the nearby hydrophobic residues. On the other hand, the isoxazole of compound 3j forms a hydrogen bond with Tyr355 and is mostly stabilized with hydrophobic contacts in the binding site (Fig. 6D).

**Fig. 7:**
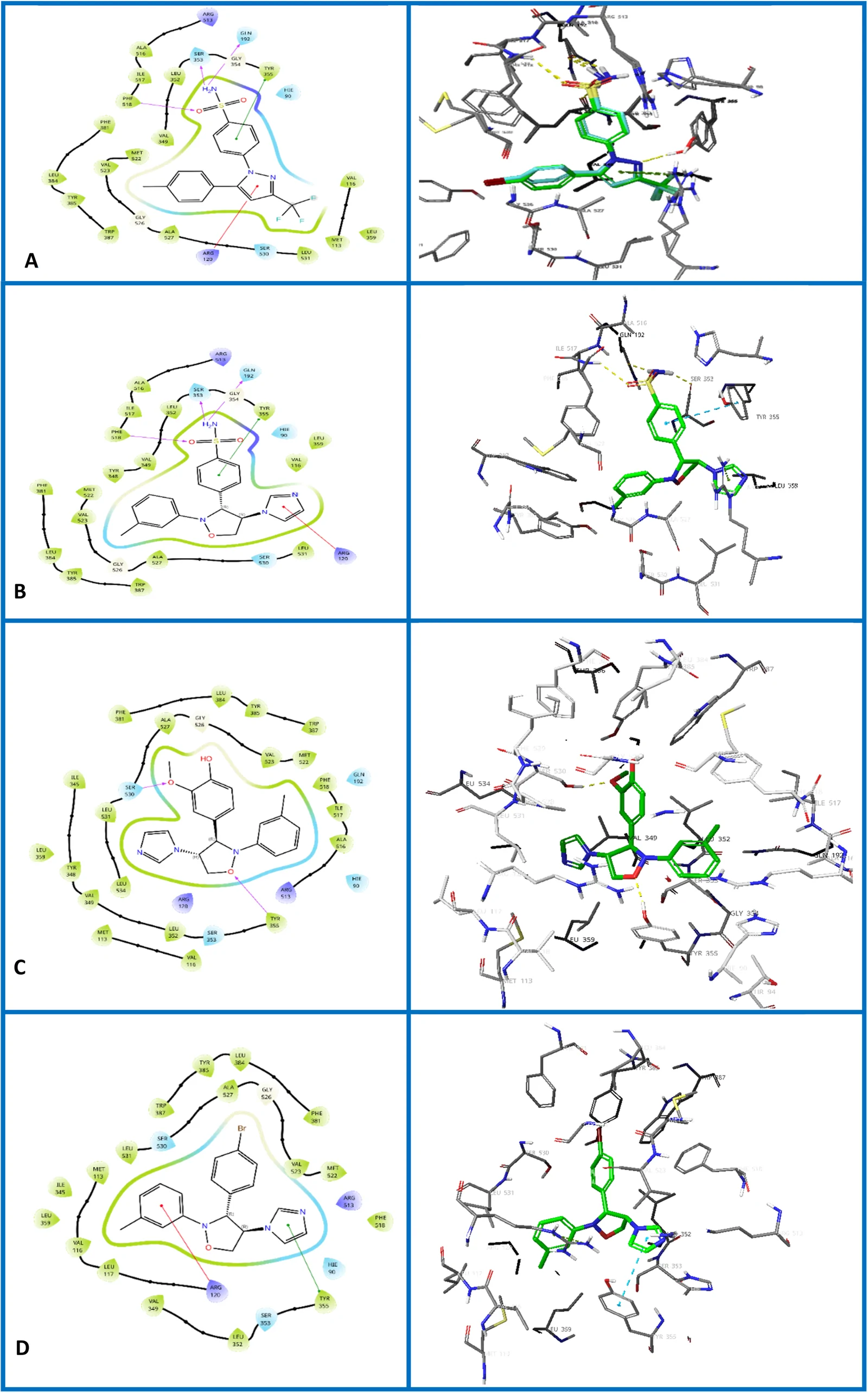
IFD docking poses of (A) celecoxib, (B) compound 3a, (C) compound 3e (D) compound 3j in COX-2 (PDB: 6COX).

The docked poses in Fig. 7 indicate that compound 3a and the reference celecoxib exhibit similar interactions and binding orientations with the cocrystallized ligand in COX-2 (PDB: 6COX). Both 3a and celecoxib formed three hydrogen bonds with the catalytic residue Phe518 and the Gln192 and Ser353 in the selectivity pocket. The favorable accommodation of the sulfonamide group in the selective pocket without forming steric clashes and the additional hydrogen bond formed with Gln192 could explain the selectivity for both celecoxib and compound 3a Fig. 7A and B. Additionally, both formed cation-π interactions with Arg120 and a π–π interaction with Tyr355, together with similar hydrophobic contacts. On the other hand, compound 3e adopted a different binding in COX-2 compared to COX-1. The presence of the smaller-sized Val523 allows the 3-methylphenyl to be accommodated in the hydrophobic region around Val523. Compound 3e interacted with the catalytic region by forming a hydrogen bond with Ser530, the entrance region by forming an additional hydrogen bond with Tyr355, and was stabilized with many hydrophobic contacts Fig. 7C. Compound 3j, the weakest inhibitor among the three compounds, interacted with the entrance region by forming a cation-π interaction with Arg120 and a π–π interaction with Tyr355 Fig. 7D.

**Fig. 8:**
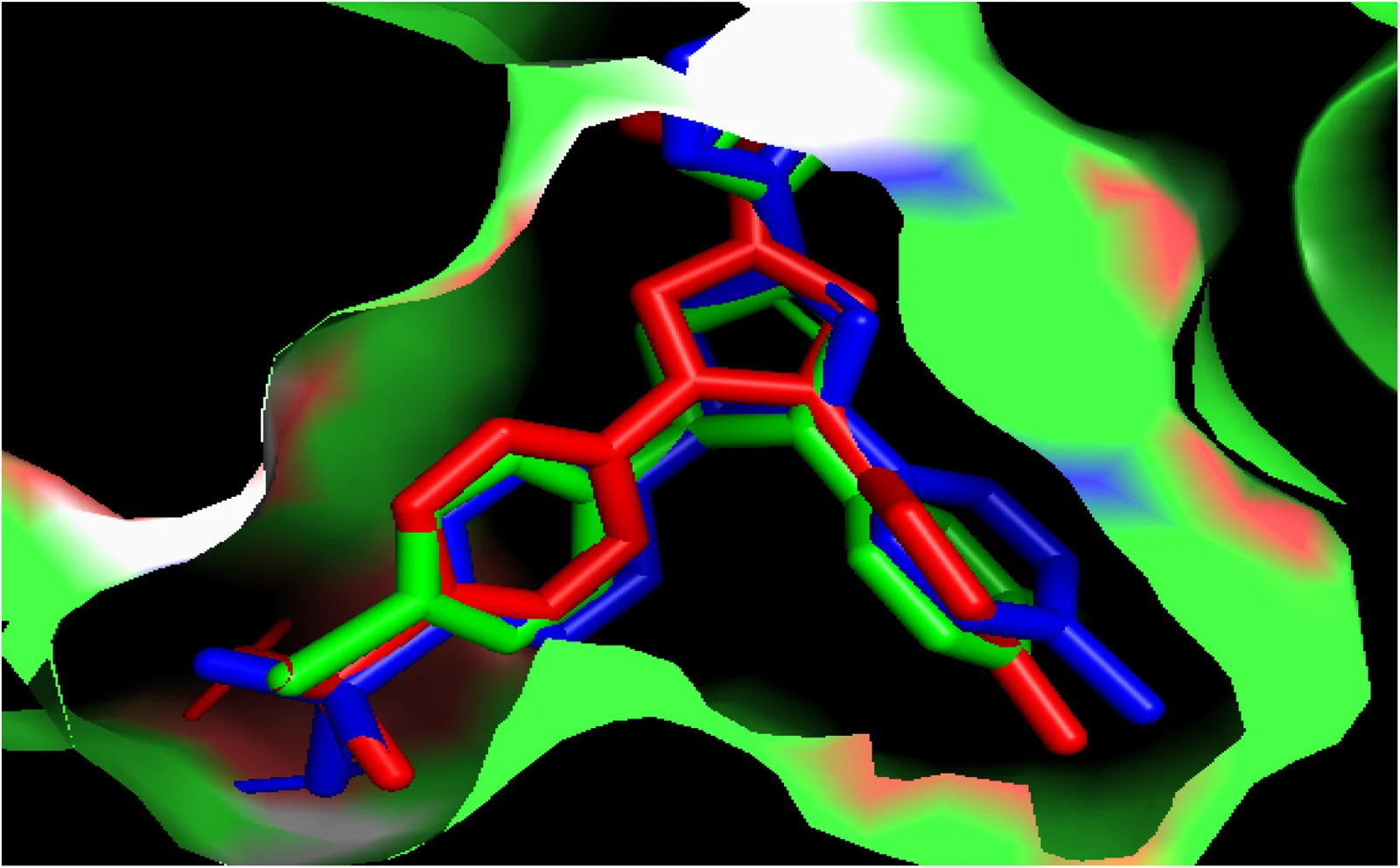
Fitting of celecoxib (red), compound 3j (green), and compound 3a (blue) to the active site of COX-II (PDB code: 6COX).

The molecular docking results, illustrated in Fig. 8, demonstrate a precise structural superimposition of the synthesized compounds 3j (green) and 3a (blue) with the reference drug celecoxib (red) within the COX-II active site (PDB code: 6COX), indicating a binding orientation highly comparable to that of celecoxib, particularly for compound 3a, which exhibited the closest overlap and most favorable accommodation within the active site. This spatial alignment reveals that compound 3a occupy the same binding pocket and exhibit a binding mode nearly identical to that of the prototype inhibitor, celecoxib. The core aromatic scaffolds of the compound 3a is accurately positioned within the catalytic cavity, which facilitates stable intermolecular interactions mimicking the reference drug's behavior. In particular, the high degree of geometric complementarity observed for compound 3a explains its potent inhibitory activity. These findings validate the molecular design strategy, confirming that compound 3a effectively act as structural mimics of celecoxib, capable of achieving strong and targeted binding to the biological receptor. The summary of the docking studies for COX-2 (PDB: 6COX) is provided in Table 3. The IFD scores were generally favourable for all ligands (−10.219 to −11.826 kcal mol^−1^), whereas the reference celecoxib showed an IFD score of −11.238 kcal mol^−1^. Similarly, the calculated MM-GBSA dG Bind of the compounds ranged between −72.71 and −57.20 kcal mol^−1^, whereas the MM-GBSA dG Bind of celecoxib was calculated as −72.60 kcal mol^−1^. Although analysis of both IFD scores and MM-GBSA dG Bind ranked compound 3a as the best compound, but predicted celecoxib to outperform compounds 3e and 3j, which is not fully correlated with the experimental data of COX-2 inhibition. However, analysis of the results within the series shows that both IFD scores and MM-GBSA dG Bind correlate the biological data for COX-2 inhibition. In addition, the MM-GBSA dG Bind energies of the compounds in COX-2 showed a more favourable binding than in COX-1 (Table 3).

**Table 3:** IFD scores, MM-GBSA dG bind values and molecular interactions of ligands-COX-1 (3KK6) and COX-2 (6COX) complexes of compounds 3a, 3e, 3j and celecoxib

Compound	COX-2 pIC_50_ ± SD		IFD score (kcal mol^−1^)		MM-GBSA dG bind (kcal mol^−1^)		Interactions	
	COX-1	COX-2	COX-1	COX-2	COX-1	COX-2	COX-1	COX-2
3a	4.19 ± 0.02	6.74 ± 0.05	−11.856	−11.826	−55.19	−72.71	Hydrogen bonds (His90, Ser516, Phe518, Ile517, Arg120)	Hydrogen bonds (Gln192, Ser353, Phe518)
							π–π interaction (Tyr355)	Cation-π interaction (Arg120)
							Hydrophobic contacts (Val116, Leu352, Val349, Tyr348, Ile523, Ala527)	π–π interaction (Tyr355)
								Hydrophobic contacts (His90, Leu352, Ile517, Val349, Val523, Ala527)
3e	4.24 ± 0.02	6.60 ± 0.05	−10.291	−10.614	−59.97	−59.29	Hydrogen bonds (Met522)	Hydrogen bonds (Ser530, Tyr355)
							π–π interaction (Tyr355)	Hydrophobic contacts (His90, Leu352, Leu531, Leu534, Phe518, Ala516, Ile517, Val349, Val523, Ala527)
							Hydrophobic contacts (His90, Leu352, Leu531, Leu534, Ala516, Val349, Ile523)	
3j	4.28 ± 0.02	6.52 ± 0.04	−9.890	−10.219	−63.40	−57.20	Hydrogen bonds (Tyr355)	Cation-π interaction (Arg120)
							Hydrophobic contacts (His90, Tyr94, Leu352, Leu534, Val349, Ile523, Ile345, Leu359)	π–π interaction (Tyr355)
								Hydrophobic contacts (His90, Val116, Leu352, Leu531, Phe518, Val349, Val523, Ala527)
Celecoxib	4.53 ± 0.03	6.04 ± 0.02	−12.174	−11.238	−57.46	−72.60	Hydrogen bonds (His90, Ser516, Phe518, Ile517)	Hydrogen bonds (Gln192, Ser353, Phe518)
							π–π interaction (Tyr355)	Cation-π interaction (Arg120)
							Hydrophobic contacts (Val349, Leu352, Leu359, Leu531, Ile517, Ile523, Ala527)	π–π interaction (Tyr355)
								Hydrophobic contacts (Val116, Trp387, Leu352, Ile517, Val349, Val523, Ala527)

**Fig. 9:**
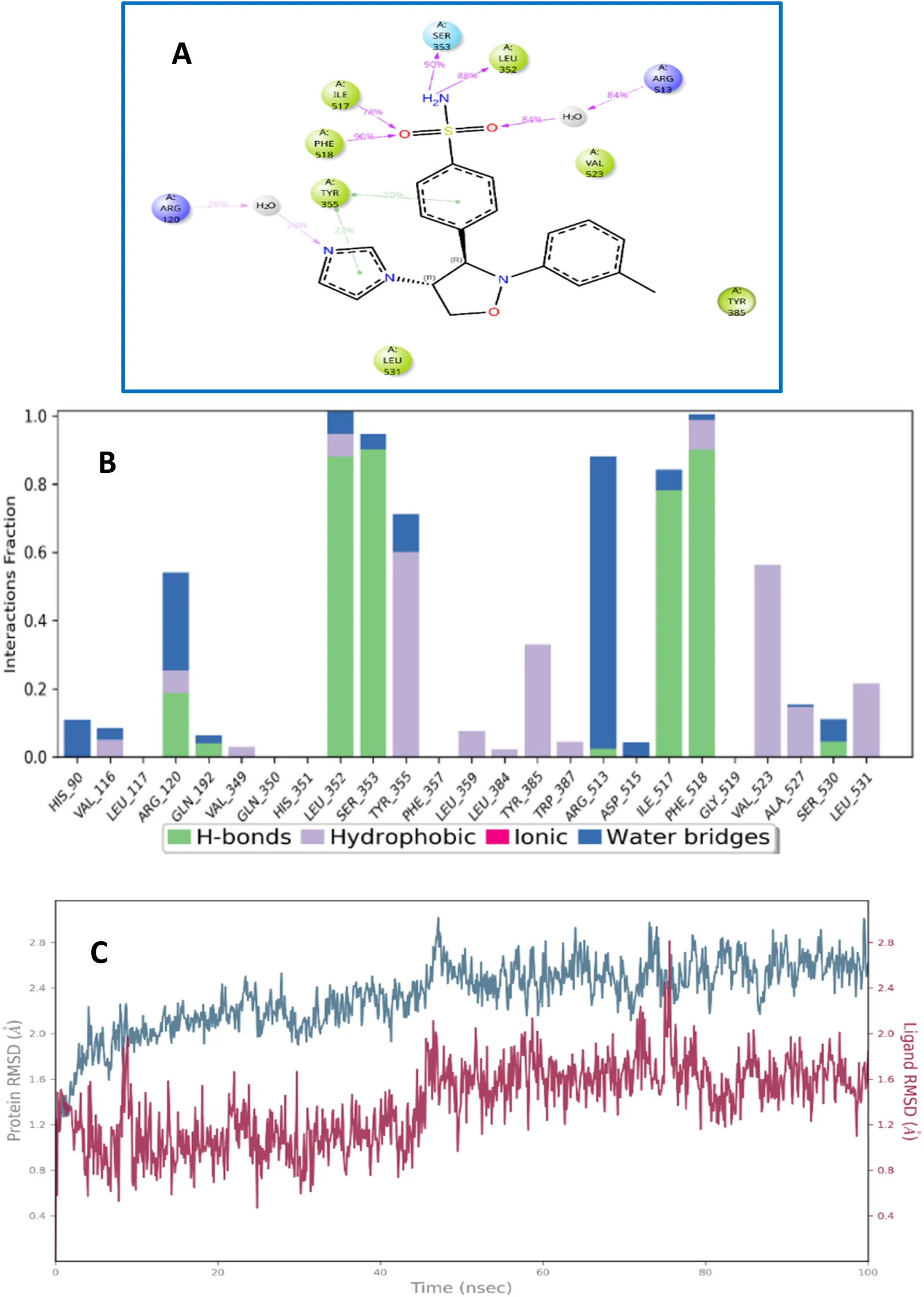
(A) A schematic representation of the ligand–protein interactions observed for at least 20% of the 100 ns MD simulation (3a-6COX), positively charged amino acid residues (purple), hydrophobic amino acid residues (green), polar amino acid residues (cyan). (B) Histogram representation of the interactions, H-bond (green), H_2_O-bridge (blue), hydrophobic contacts (grey). (C) RMSD plot of the protein Cα-atoms (blue) and ligand (red).

To analyze the stability of the generated IFD poses for compounds 3a, 3e and 3j in COX-2 (PDB: 6COX), a 100 ns MD simulation was conducted using the Desmond platform in Schrödinger. The analyses of the results for compound 3a show that the hydrogen bond formed with Ser353 and Phe518 lasted for almost the entire simulation period (90% occupancy). However, the hydrogen bond formed with Gln192 was transient and compensated with Leu352, which continuously interacted with the compound throughout the simulation. The hydrophobic contact with Tyr355 and Val523 continued for more than half the simulation period. The cation-π interaction with Arg120 was changed and continued as an H_2_O-bridged hydrogen bond (>50% occupancy), and a new H_2_O-bridged hydrogen bond formed and continued for 84% of the simulation (Fig. 9A and B). Fig. 9C reveals the protein RMSD increases quickly at the beginning (early equilibration) and then fluctuates around a stable 2.2–2.7 Å for the second half of the simulation, suggesting that the protein initially underwent normal relaxation but retained its overall structure. Similarly, the ligand RMSD gradually increases in the first half of the simulation, albeit visual inspection of the trajectory reveals that the compound remains in its original pose, and the increase in the RMSD was the constant movement of the imidazole ring at the entrance region, facing the solvent-exposed region. Overall, the ligand stabilized around 1.6 Å (Fig. 9C).

**Fig. 10:**
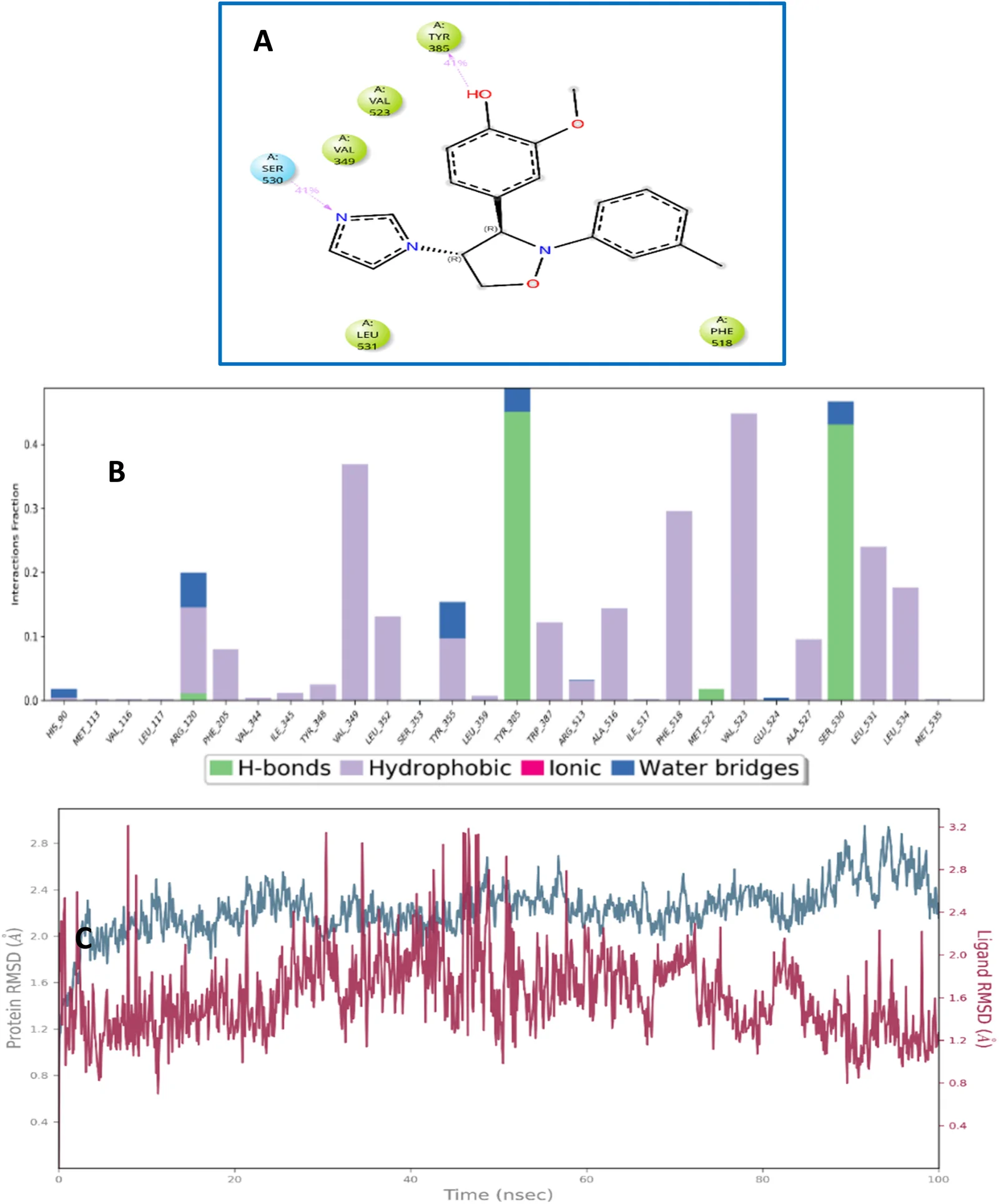
(A) A schematic representation of the ligand–protein interactions observed for at least 20% of the 100 ns MD simulation (3e-6COX), hydrophobic amino acid residues (green), polar amino acid residues (cyan). (B) Histogram representation of the interactions, H-bond (green), H_2_O-bridge (blue), hydrophobic contacts (grey). (C) RMSD plot of the protein Cα-atoms (blue) and ligand (red).

Fig. 10 shows that the formed hydrogen bonds in the docked pose between Ser530 and the methoxy and between Tyr355 and isooxazoline ring compound 3e were transient and did not last, however, both amino acid residues compensated these interactions with new hydrogen bonds formed between the imidazole ring (46% occupancy) and phenolic OH (41% occupancy), respectively (Fig. 10A). The hydrophobic contacts with Leu531, Phe518, Val349 and Val523 lasted for 25%, 30%, 35% and 45%, respectively (Fig. 10B). This shows that the ligand was mainly stabilized with hydrophobic contacts. The RMSD plot reveals the ligand fluctuates between 1.2–3.2 Å due to high motion of the imidazole and the 3-methylphenyl ring. The protein RMSD also remained stable below 2.8 Å (Fig. 10C).

**Fig. 11:**
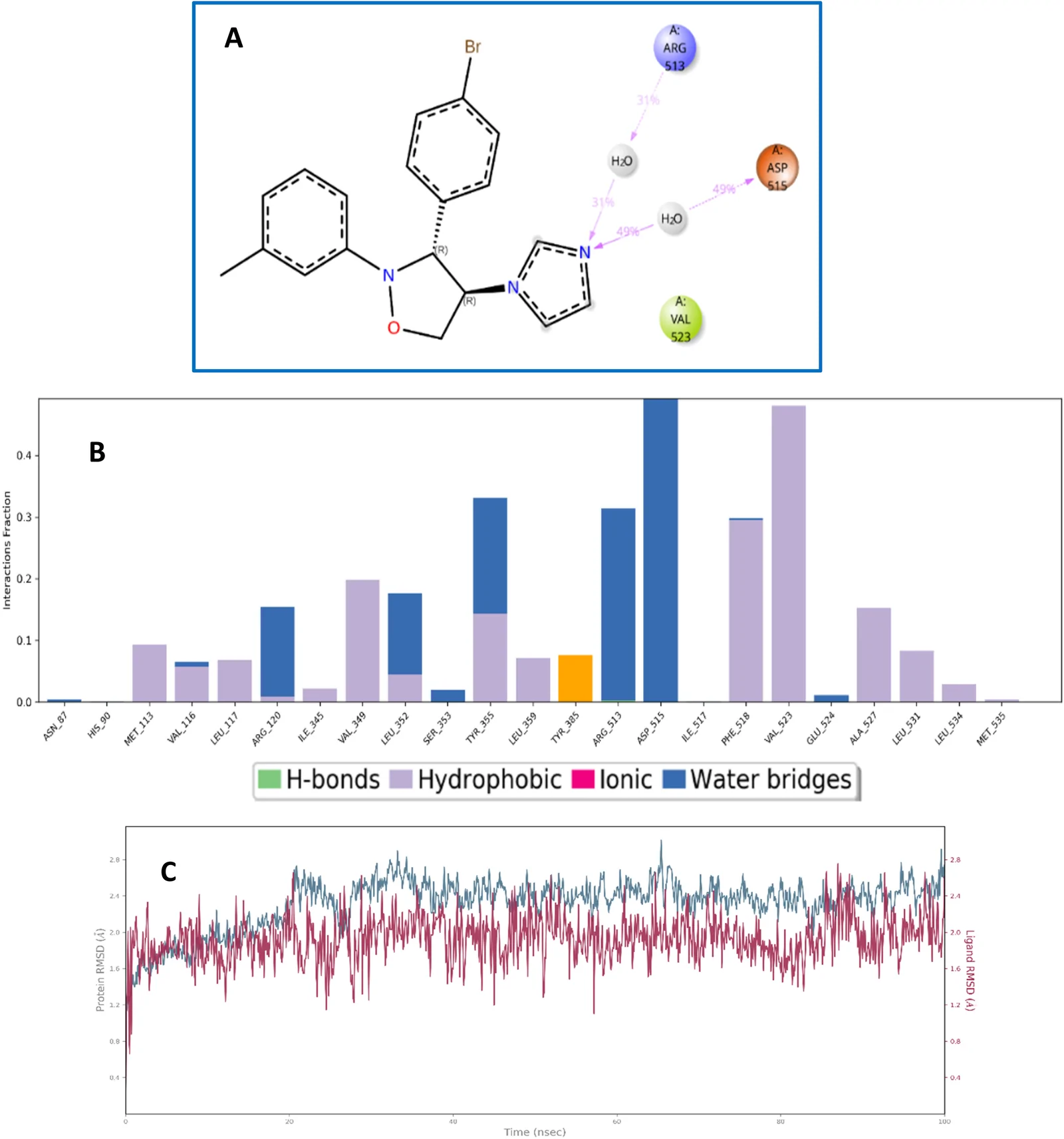
(A) A schematic representation of the ligand–protein interactions observed for at least 20% of the 100 ns MD simulation (3j-6COX), positively charged amino acid residues (purple), negatively charged amino acid residues (orange), hydrophobic amino acid residues (green), polar amino acid residues (cyan). (B) Histogram representation of the interactions, H-bond (green), H_2_O-bridge (blue), hydrophobic contacts (grey), halogen bond (orange). (C) RMSD plot of the protein Cα-atoms (blue) and ligand (red).

The 100 ns MD simulation analysis of 3j-6COX complex reveals that the initial interactions formed with Arg120 and Tyr355 were transient in the early stages of the simulation. However, new H_2_O-bridged hydrogen bond with Arg513 and Asp515 were formed and continued for 31% and 49%, respectively (Fig. 11A). The histogram (Fig. 11B) shows that binding is largely driven by hydrophobic contacts. The hydrophobic contacts formed with Val349, Phe528 and Val523 were present for 20%, 30% and 48%, respectively (Fig. 5B). The RMSD plot reveals that both protein and the ligand were stable below 2.8 Å (Fig. 11C). Generally, compound 3a, which formed multiple interactions with the selective, the hydrophobic and the entrance regions showed a better persistent interaction with the protein during the 100 ns MD simulation. Secondly, compound 3e and 3j are mostly stabilized with hydrophobic contacts with fewer persistent interactions during the 100 ns MD simulations. These results align with the inhibition data presented in Table 1.

### 3.6. DFT calculation

#### 3.6.1. The theoretical geometry analysis

**Fig. 12:**
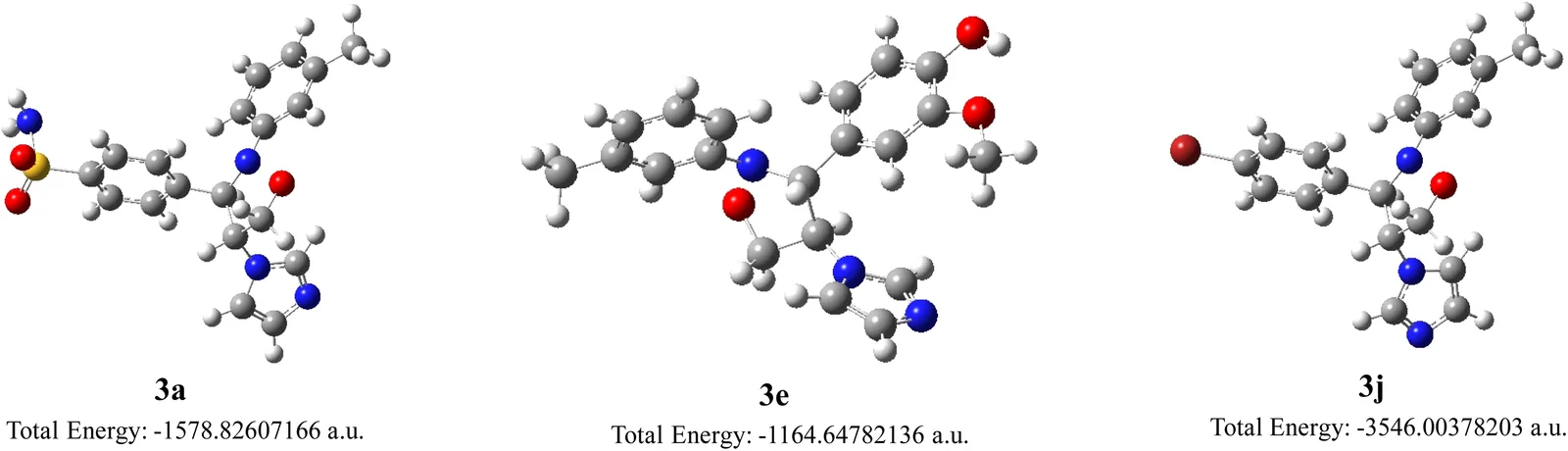
Optimized molecular structures and total energy values of active compounds.

The equilibrium geometries of the selected derivatives (3a, 3e, and 3j) were fully optimized using density functional theory at the B3LYP/6-31G(d,p) level. Geometry optimizations were performed without any symmetry or structural constraints, allowing the molecular frameworks to adopt their most energetically favorable conformations. The optimized structures obtained for all compounds are depicted in Fig. 12. Convergence was successfully achieved for each molecule, and the absence of imaginary frequencies confirmed that the optimized geometries correspond to true minimum on the potential energy surface. The calculated total electronic energies indicate noticeable differences in the intrinsic stability of the investigated derivatives. Compound 3j exhibits the lowest total energy (−3546.0038 a.u), which can be attributed to its larger molecular framework and increased contribution from heteroatoms and substituent interactions. In comparison, compound 3a shows a total energy of −1578.8261 a.u., reflecting a well-stabilized structure with an extended conjugated system. The comparatively higher energy observed for compound 3e (−1164.6478 a.u.) is consistent with its reduced molecular size and lower degree of electronic delocalization.

Overall, the optimized geometries reveal that both molecular size and substituent distribution play a crucial role in determining the energetic stability of the studied compounds, providing a reliable structural basis for subsequent electronic and reactivity analyses.

#### 3.6.2. Dipole moments

The molecular dipole moment represents an important electronic parameter that reflects the degree of charge separation within a compound and provides insight into its polarity and intermolecular interaction potential. The individual dipole moment components along the Cartesian axes, together with the resulting total dipole moments for the investigated derivatives, are reported in Table 4. Among the studied compounds, derivative 3a exhibits the highest overall dipole moment (*µ*_tot_ = 2.91 D). This value mainly arises from a pronounced contribution along the *y*-axis (*µ*_*y*_ = −2.85 D), indicating a directional polarization across the molecular backbone. Such as an asymmetric charge distribution may favor electrostatic interactions and contribute to enhanced affinity toward polar environments or biological targets. Compound 3e displays a slightly lower total dipole moment (*µ*_tot_ = 2.20 D), with comparable contributions from all three spatial components. The presence of significant dipole vectors along the *x*, *y*, and *z* axes suggests a more evenly distributed polarization, which may influence its conformational behavior and interaction profile differently from compound 3a. In contrast, compound 3j presents the lowest dipole moment within the series (*µ*_tot_ = 1.66 D). Although noticeable dipole components are observed along the *y* and *z* directions, their partial compensation results in a reduced overall polarity. This more balanced electronic distribution may impact molecular recognition processes and intermolecular packing properties. Overall, the observed differences in dipole moment values among compounds 3a, 3e, and 3j highlight the influence of structural features and substituent orientation on electronic polarization. These variations are expected to play a role in modulating physicochemical properties, intermolecular interactions, and potential biological behavior of the investigated derivatives.

**Table 4:** The values of the electric dipole moment of the active compounds

Compounds	*µ*_*x*_ (Debye)	*µ*_*y*_ (Debye)	*µ*_*z*_ (Debye)	*µ*_tot_ (Debye)
3a	0.4285	−2.8510	0.4037	2.9111
3e	0.8935	1.2556	1.5641	2.1958
3j	−0.3685	−1.9608	1.6564	1.6564

#### 3.6.3. Frontier molecular orbitals

**Fig. 13:**
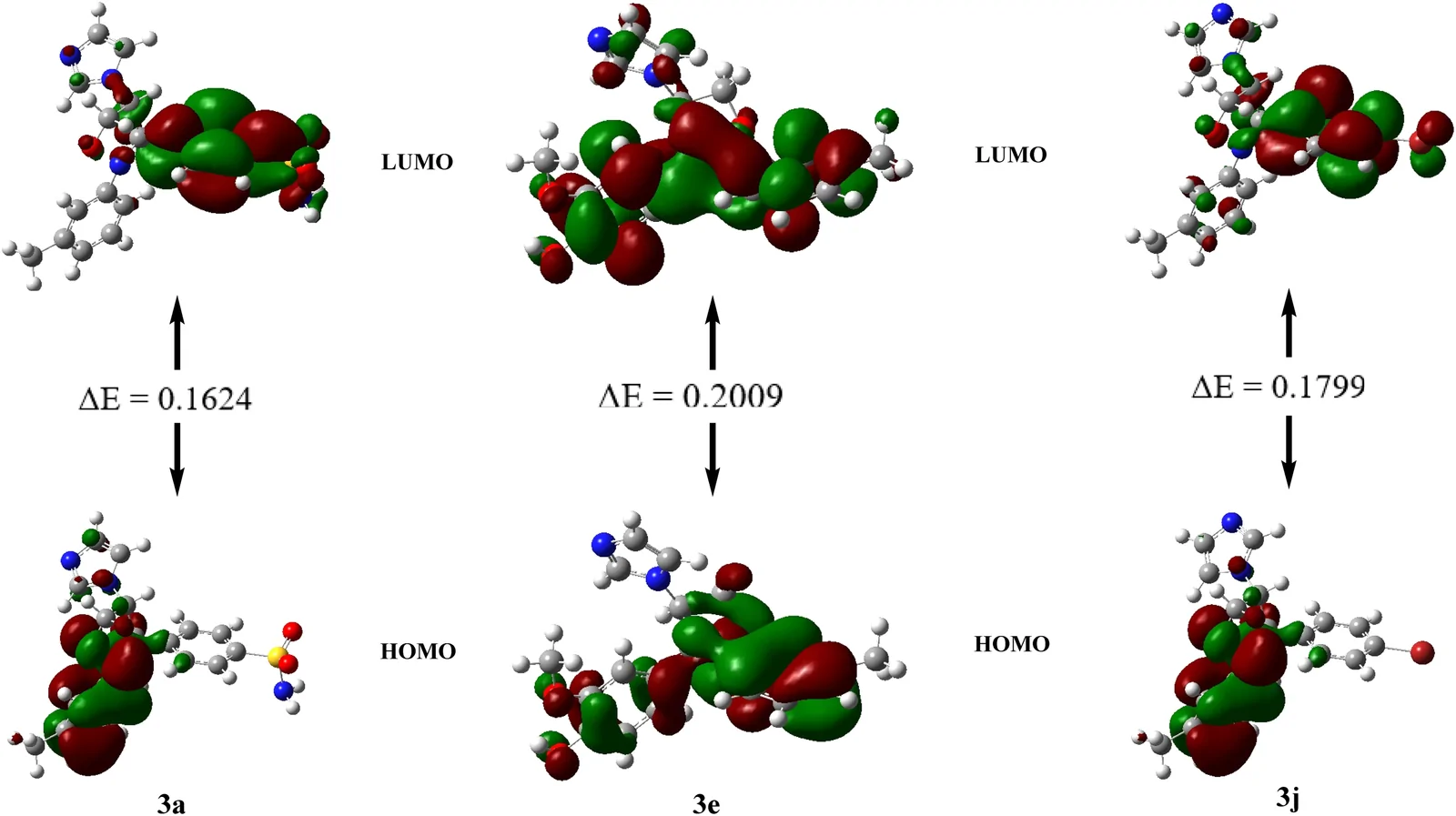
HOMO–LUMO diagrams of the active compounds.

The electronic structure and reactivity features of compounds 3a, 3e, and 3j were further investigated through frontier molecular orbital (FMO) analysis combined with global reactivity descriptors derived from conceptual density functional theory (DFT). The energies of the highest occupied molecular orbital (HOMO) and the lowest unoccupied molecular orbital (LUMO), together with related parameters such as ionization potential, electron affinity, electronegativity, chemical hardness and softness, chemical potential, electrophilicity index, and the HOMO–LUMO energy gap, provide a comprehensive framework for interpreting the intrinsic electronic behavior of the studied molecules (Table 5) and (Fig. 13). The HOMO–LUMO energy gap (Δ*E*) is widely regarded as a key descriptor of molecular reactivity, as it reflects the energetic cost associated with intramolecular charge transfer. In the present series, compound 3a exhibits the smallest Δ*E* value, indicating that electronic excitation from the HOMO to the LUMO requires comparatively lower energy. This feature suggests enhanced electronic flexibility and a greater propensity for charge redistribution under external perturbations. Such characteristics are often associated with increased polarizability and a higher likelihood of participating in electron-transfer processes, which may be relevant in both chemical and biological interaction contexts. In contrast, compound 3e displays the largest HOMO–LUMO separation, pointing to a more electronically stable system. A larger Δ*E* typically implies reduced reactivity, as excitation of an electron into the antibonding LUMO orbital is energetically less favorable. This behavior suggests that 3e possesses a more rigid electronic structure and a diminished tendency to undergo charge-transfer events. Compound 3j occupies an intermediate position, exhibiting a HOMO–LUMO gap that lies between those of 3a and 3e, reflecting a balance between electronic stability and reactivity. These trends are further corroborated by the values of chemical hardness (*η*) and softness (*S*), which provide complementary insight into the resistance of a molecule toward changes in its electron density. Compound 3a shows the lowest hardness and the highest softness among the three derivatives, reinforcing its classification as the most electronically adaptable molecule in the series. Soft molecules are generally more responsive to external electric fields and can more readily adjust their electron density during intermolecular interactions. On the other hand, compound 3e exhibits the highest hardness and the lowest softness, consistent with a more conservative electronic character and reduced adaptability. Again, compound 3j demonstrates intermediate behavior, suggesting a moderate ability to accommodate electronic perturbations. Ionization potential (*I*) and electron affinity (*A*) provide additional detail regarding the electron-donating and electron-accepting capabilities of the compounds. The ionization potentials of 3a, 3e, and 3j are relatively close in magnitude, indicating that removal of an electron from the HOMO requires a similar energetic investment across the series. This observation suggests that the electron-donating ability of the compounds does not differ substantially. In contrast, more pronounced differences are observed in electron affinity. Compound 3a exhibits the highest electron affinity, indicating a stronger ability to accept and stabilize additional electron density. Compound 3j shows a moderate electron affinity, whereas compound 3e presents a notably low value, suggesting a weak tendency to accommodate an incoming electron. This low electron affinity further supports the interpretation of 3e as the most electronically stable and least electrophilically responsive derivative. Electronegativity (*χ*) and chemical potential (*µ*) capture the overall tendency of a molecule to attract electrons and its energetic inclination toward electron exchange with the environment. In this respect, compound 3a displays the highest electronegativity and the most negative chemical potential, reflecting a strong driving force for electron acceptance. Such features indicate that 3a is more likely to engage in interactions governed by electrostatic or charge-transfer contributions. Compound 3j again shows intermediate values, while compound 3e exhibits the lowest electronegativity and the least negative chemical potential, consistent with its reduced electrophilic character. The electrophilicity index (*ω*), which integrates chemical potential and hardness into a single descriptor, provides a consolidated measure of a molecule's capacity to stabilize electronic charge upon interaction. In the present study, compound 3a exhibits the highest electrophilicity index, confirming its pronounced electrophilic nature and its enhanced ability to accommodate additional electron density. Compound 3j displays moderate electrophilicity, suggesting a balanced electronic profile, while compound 3e shows the lowest *ω* value, in agreement with its larger HOMO–LUMO gap, higher hardness, and minimal electron affinity.

**Table 5:** Some reactivity parameters of the active compounds

Compounds	*E*_HOMO_ (Ha)	*E*_LUMO_ (Ha)	Δ*E* (Ha)	*I* (Ha)	*A* (Ha)	*χ* (Ha)	*η* (Ha)	*S* (Ha^−1^)	*µ* (Ha)	*ω* (Ha)
3a	−0.2117	−0.0493	0.1624	0.2117	0.0493	0.1305	0.0812	6.1576	−0.1305	0.1049
3e	−0.2054	−0.0045	0.2009	0.2054	0.0045	0.1050	0.1004	4.9776	−0.1050	0.0548
3j	−0.2096	−0.0297	0.1799	0.2096	0.0297	0.1196	0.0900	5.5586	−0.1196	0.0796

Taken together, the FMO-based descriptors consistently differentiate the electronic profiles of the three compounds. Compound 3a emerges as the most electronically reactive and adaptable derivative, characterized by a small energy gap, high softness, strong electron-accepting tendency, and elevated electrophilicity. Compound 3e represents the opposite extreme, with greater electronic stability, reduced polarizability, and limited electrophilic responsiveness. Compound 3j occupies an intermediate position, combining moderate stability with appreciable electronic flexibility. These theoretical insights provide a coherent electronic rationale for the observed differences in physicochemical behavior across the series and offer a solid basis for interpreting potential variations in chemical reactivity and biological performance.

#### 3.6.4. Molecular electrostatic potentials values (MEP)

**Fig. 14:**
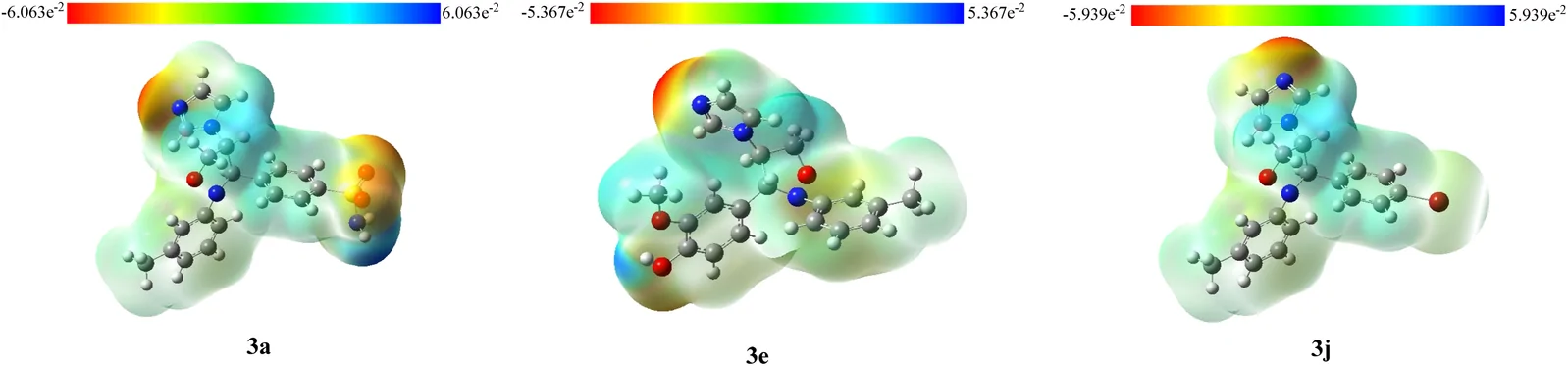
Molecular electrostatic potential (MEP) surfaces of the active compounds.

The molecular electrostatic potential (MEP) surfaces of compounds 3a, 3e, and 3j were generated to visualize the charge distribution and to identify regions susceptible to electrophilic and nucleophilic interactions (Fig. 14). MEP mapping is a valuable tool for correlating molecular structure with reactivity, as it provides direct insight into the spatial variation of electrostatic potential over the molecular surface. In all three compounds, the color-coded MEP surfaces reveal a heterogeneous distribution of electron density. Regions depicted in red correspond to areas of negative electrostatic potential, indicating electron-rich zones that are more prone to electrophilic attack, while blue regions represent electron-deficient areas favorable for nucleophilic interactions. Intermediate colors (green to yellow) denote regions of near-neutral potential.

For compound 3a, pronounced negative potential regions are mainly localized around heteroatom-containing functionalities, particularly near electronegative oxygen and nitrogen atoms. These electron-rich sites suggest a strong capacity for intermolecular interactions such as hydrogen bonding or electrostatic attraction with positively charged or electron-deficient species. The presence of well-defined positive potential regions elsewhere on the molecular surface indicates clear polarization, consistent with its relatively high softness and electrophilicity derived from the FMO analysis. Compound 3e exhibits a comparatively more uniform electrostatic potential distribution. Although negative regions are still associated with heteroatoms, their intensity is noticeably reduced, and the overall surface appears more evenly balanced. This behavior reflects a lower degree of electronic polarization, which aligns well with the higher HOMO–LUMO gap and increased electronic hardness observed for this compound. Such characteristics suggest reduced reactivity toward charge-driven interactions. In contrast, compound 3j displays an intermediate MEP profile. Distinct electron-rich regions are present around key heteroatoms, while positive potential zones are distributed across other parts of the molecular framework. This balanced electrostatic pattern indicates moderate polarity and a capacity for both electrophilic and nucleophilic interactions, supporting its intermediate reactivity profile relative to 3a and 3e. Overall, the MEP analysis highlights clear differences in charge distribution among the studied compounds. The more pronounced polarization observed for 3a suggests enhanced interaction potential, whereas the smoother electrostatic surface of 3e reflects greater electronic stability. Compound 3j combines features of both extremes, exhibiting a balanced electrostatic character. These findings provide complementary theoretical support for the trends observed in the global reactivity descriptors and contribute to a deeper understanding of structure–property relationships within this series.

## 4. Conclusion

In this study, a novel series of tricyclic isoxazolidine–imidazole derivatives bearing diverse substituted phenyl groups were successfully designed, synthesized, and fully characterized. The regio- and diastereoselective 1,3-dipolar cycloaddition approach provided structurally well-defined isoxazolidine analogues in good to excellent yields. Spectroscopic analyses FTIR, ^1^H NMR, ^13^C NMR, and LC-MS confirmed the formation and stereochemistry of the target compounds. Biological evaluation revealed that several derivatives exhibited potent COX-2 inhibitory activity with minimal COX-1 inhibition, resulting in high selectivity indices. Notably, compounds 3a, 3e, and 3j exhibited higher COX-2 selectivity than the reference drug celecoxib. Molecular docking studies provided insights into the key binding interactions within the COX-2 active site, highlighting the role of steric and electronic complementarity in achieving isoform selectivity. The *in silico* ADMET analysis indicated favorable drug-likeness, oral bioavailability, pharmacokinetic properties, and potential safety profiles, supporting their suitability as oral therapeutic candidates. This integrated synthetic, biological, and computational investigation identified promising lead compounds with potent COX-2 inhibitory activity, high selectivity, and favorable pharmacological characteristics, providing a strong foundation for further structural optimization and the development of safer and more effective anti-inflammatory agents. Furthermore, density functional theory (DFT) calculations demonstrated that compound 3a was the most reactive and electrophilic derivative, whereas compound 3e exhibited greater electronic stability, as supported by frontier molecular orbital analysis and molecular electrostatic potential mapping. These findings provide valuable structure–property relationships that may facilitate the rational design of future isoxazolidine-based COX-2 inhibitor.

## Conflicts of interest

There are no conflicts to declare.

## Supplementary Material

RA-016-D6RA03616C-s001

## Data Availability

The data supporting the findings of this study are available within the article and its supplementary information (SI). Additional experimental data (including synthesis and biological assay results) and computational data (including molecular docking and modeling files) are available from the corresponding author upon reasonable request. Supplementary information is available. See DOI: https://doi.org/10.1039/d6ra03616c.
