# Supplementary material for: Design and synthesis of isoxazolidine core-based selective COX-2 inhibitors: in vitro inhibitor activity and in silico modeling
Source: RSC Adv. 2026 Aug 10;16(40):46389–415. doi: 10.1039/d6ra03616c (PMC13455798; doi:10.1039/d6ra03616c)
Supplement: Supplementary file 1 [file RA-016-D6RA03616C-s001.pdf]

This journal is © The Royal Society of Chemistry 2026

Supplementary file

Design and Synthesis of Isoxazolidine Core-Based Selective COX-2 Inhibitors: In Vitro Inhibitor Activity and In Silico Modeling

Arwa ALAdhreai [a,f], Mohammed ALSaeedy* [a,f], Demokrat Nuha [b], Muhammed Trawally [c], Ramzan ullah [d], Syed Mansoor Ali [e], ZabnAllah M. Alaizeri h [e], Selahaddin Güner [f], Mansour K. Gatasheh ** [g]

[a] *Department of Chemistry "Giacomo Ciamician" University of Bologna, 40126 Bologna BO, Italy*
[b] *University for Business and Technology, Faculty of Medical Biochemistry, Prishtina, Kosovo.*
[c] *Department of Pharmaceutical Chemistry, Faculty of Pharmacy, Istanbul University, Istanbul, Türkiye*
[d] *Shanghai Ultra-Precision Optical Manufacturing Engineering Center, Department of Optical Science and Engineering, Fudan University, Shanghai 200433, China.*
[e] *Department of Physics and Astronomy, College of Science, King Saud University, Riyadh 11451, Saudi Arabia.*
[f] *Kocaeli University, Faculty of Arts & Sciences, Department of Chemistry, 41001 Umuttepe-Kocaeli, Turkey.*
[g] *Department of Biochemistry, College of Science, King Saud University, P.O. Box 2455, Riyadh, 11451, Saudi Arabia*

IR, ^{1}H–NMR, and ^{13}C–NMR spectroscopies for nitrones

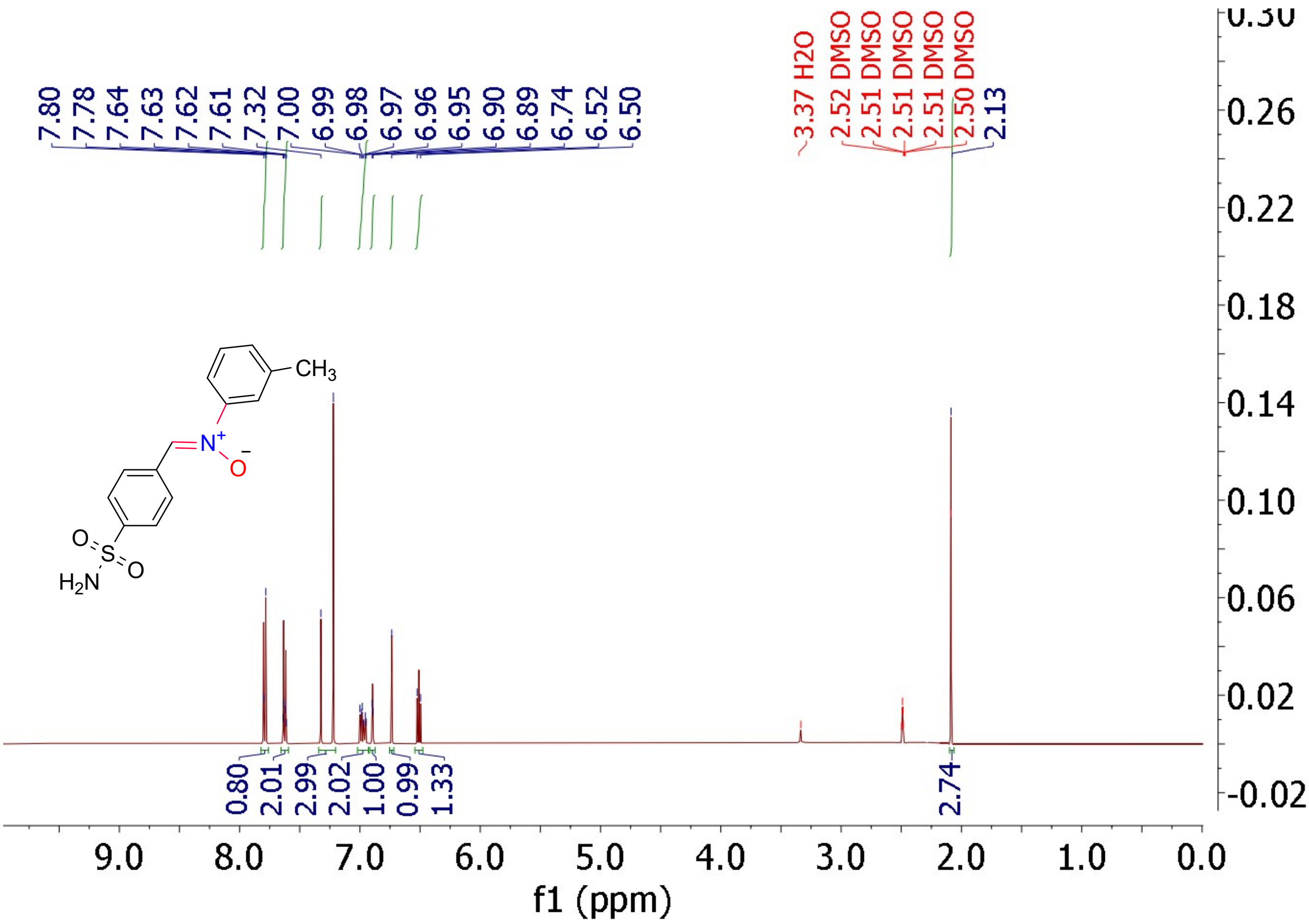

Figure 1: ^{1}H-NMR of nitrone (1a)

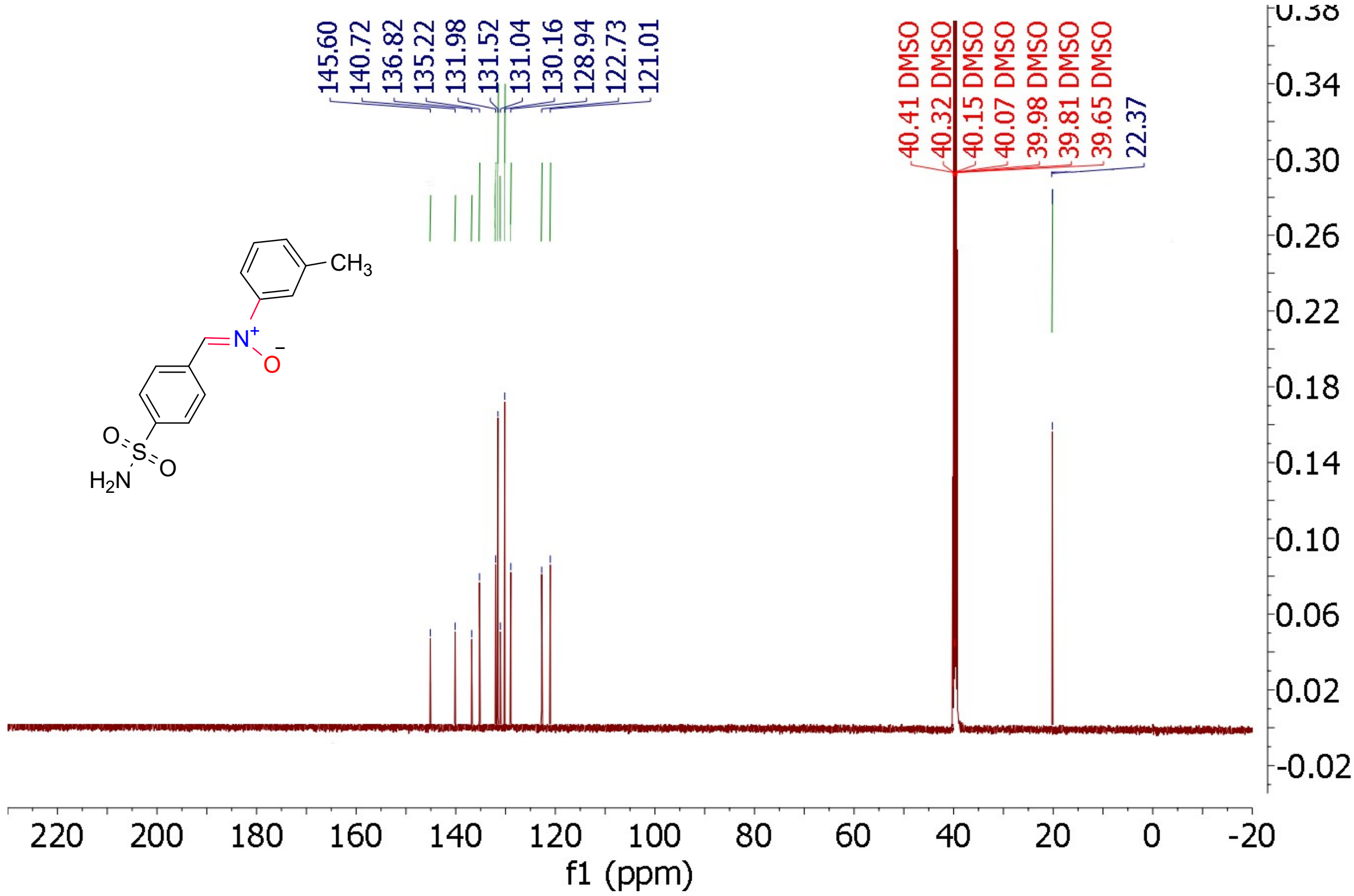

Figure 2: ^{13}C-NMR for nitrone (1a)

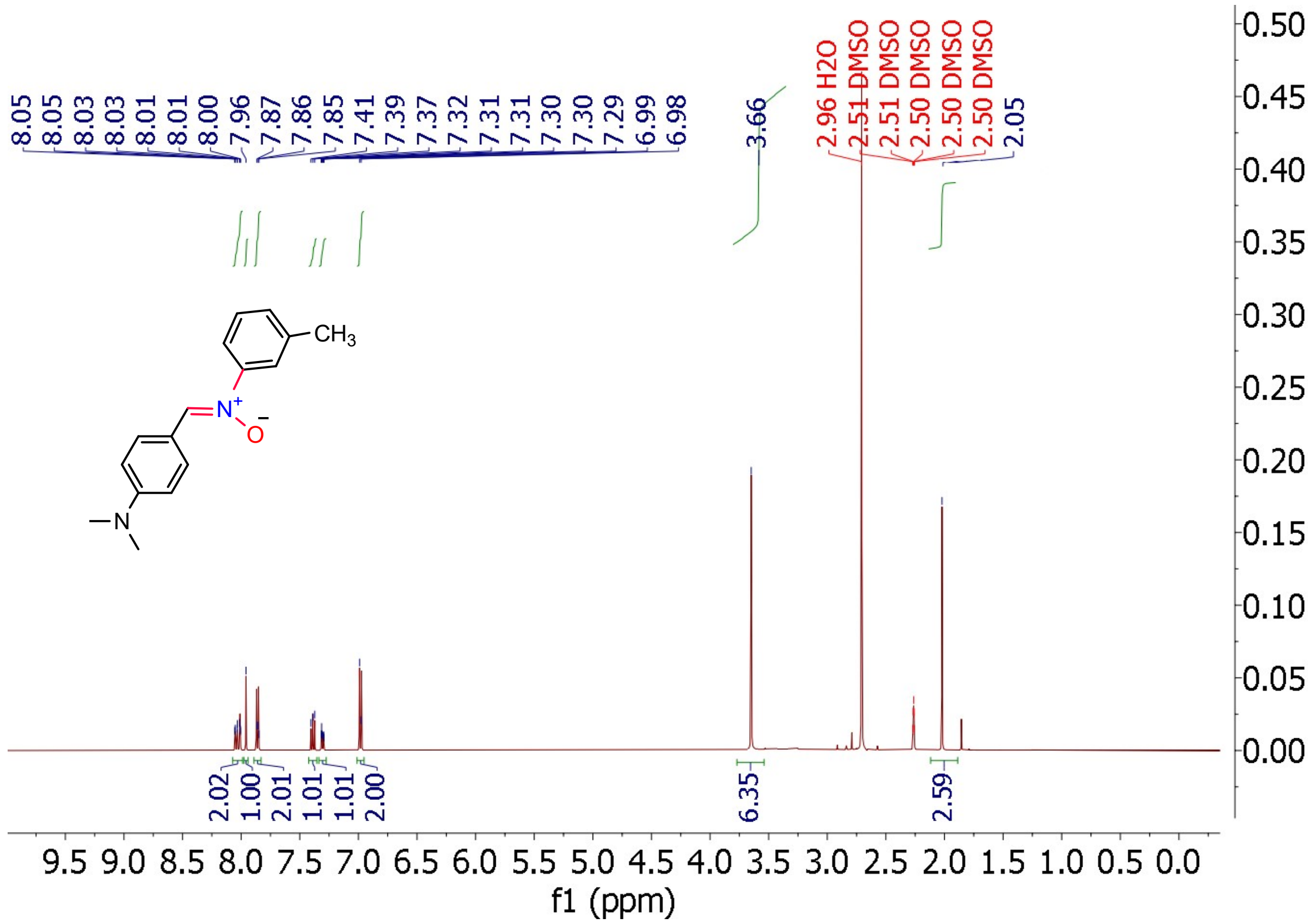

Figure 3: ^{1}H-NMR of nitrone (1b)

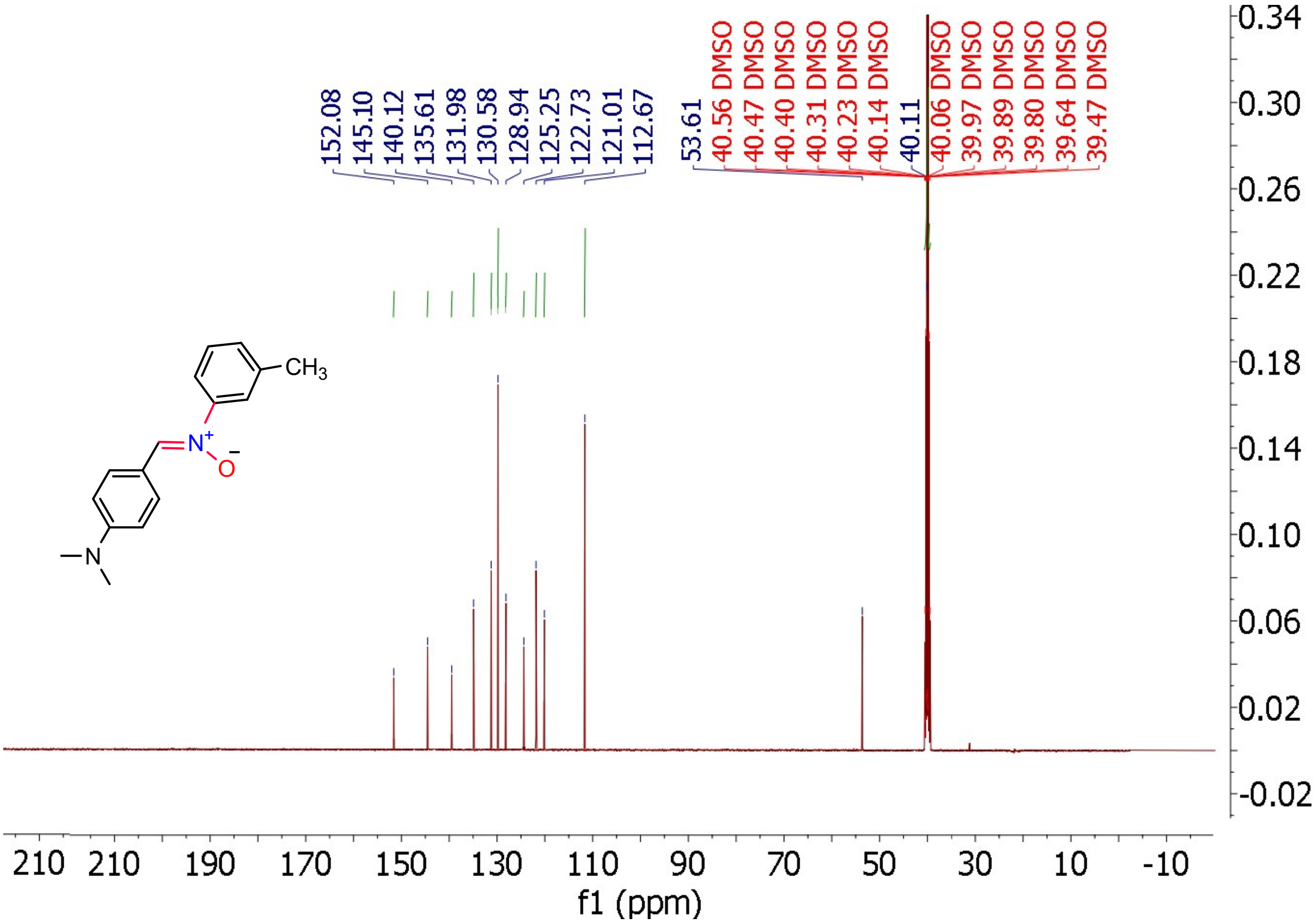

Figure 4: ^{13}C-NMR for nitrone (1b)

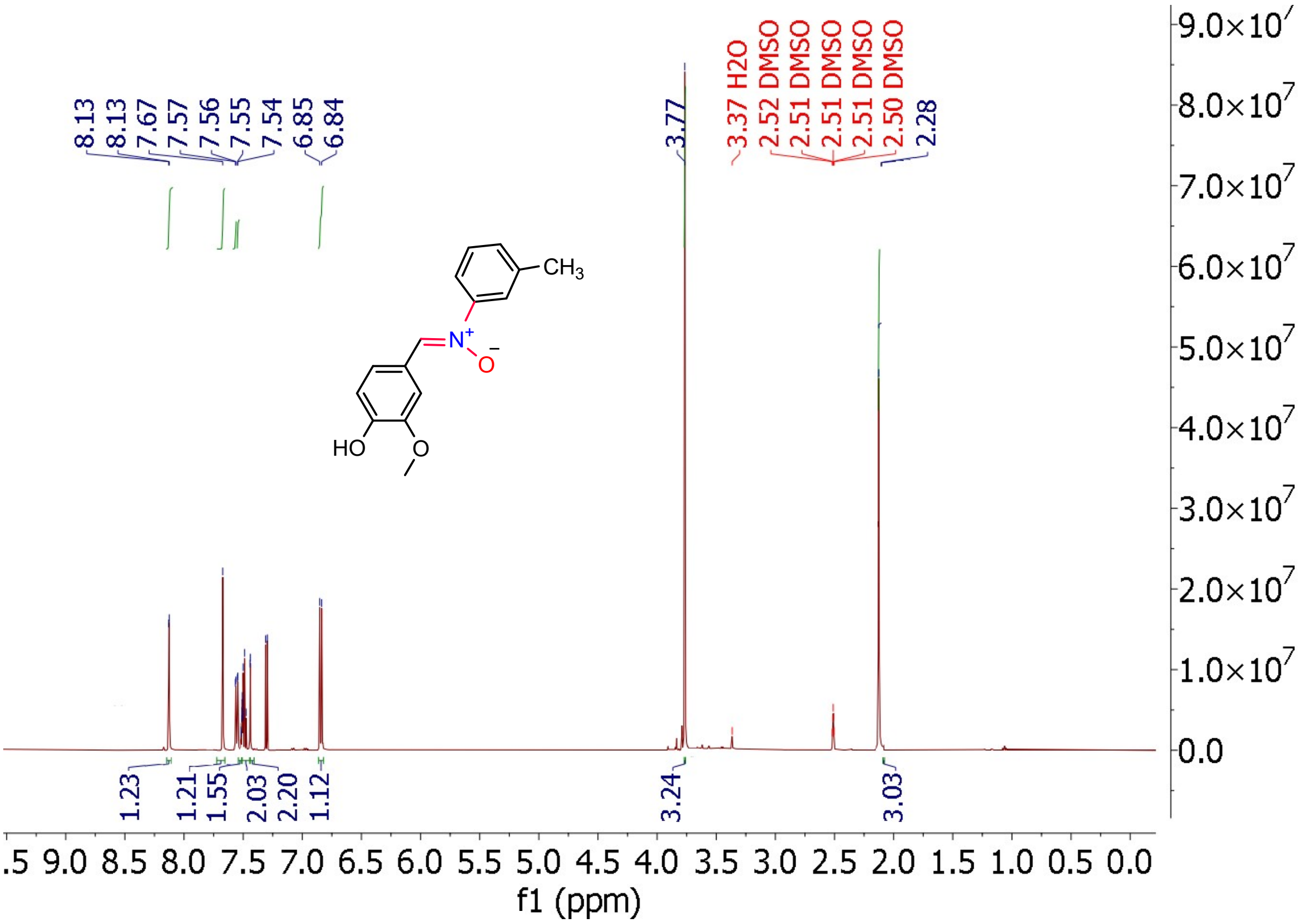

Figure 5: ^{1}H-NMR of nitrone (1e)

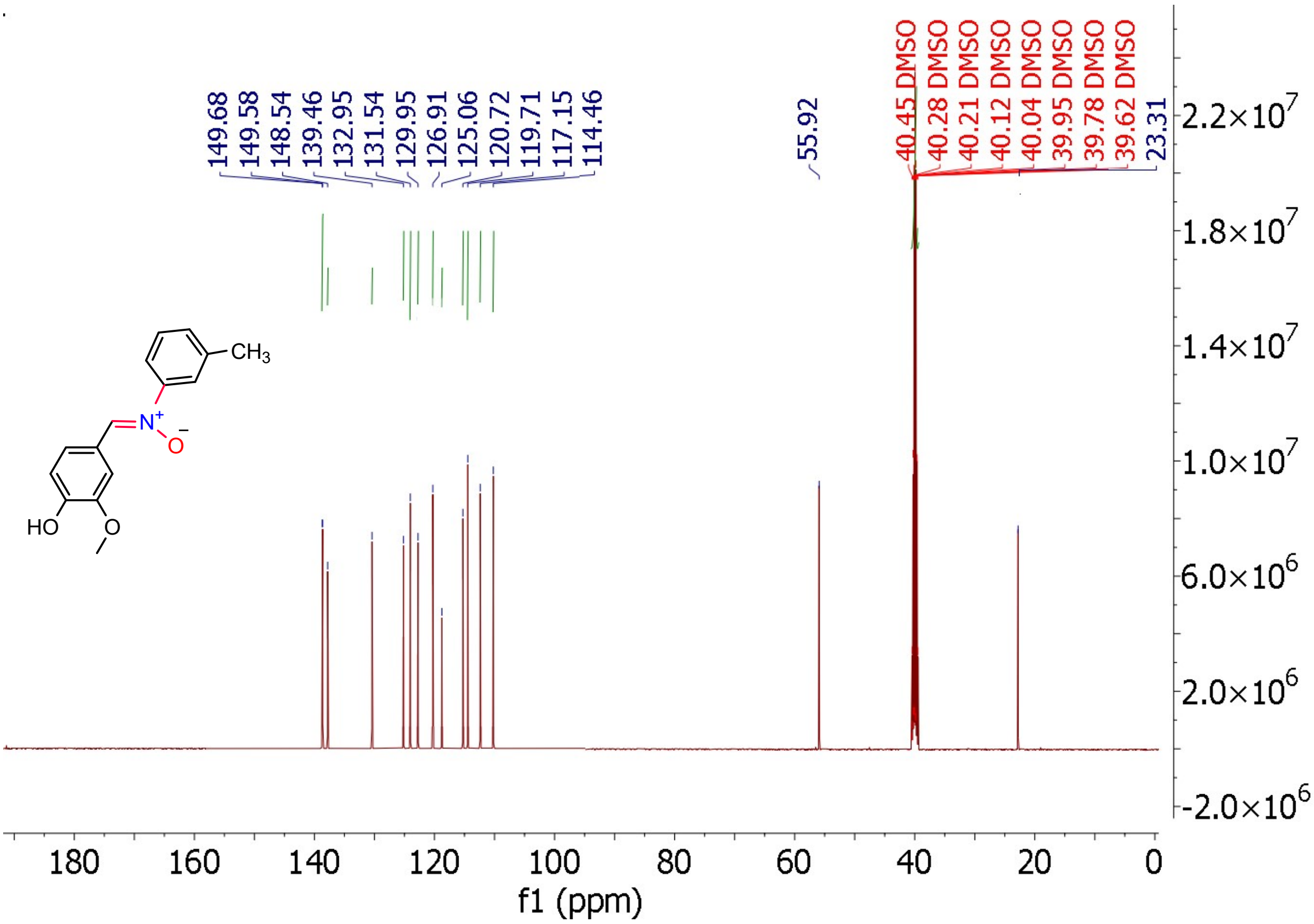

Figure 6: ^{13}C-NMR for nitrone (1e)

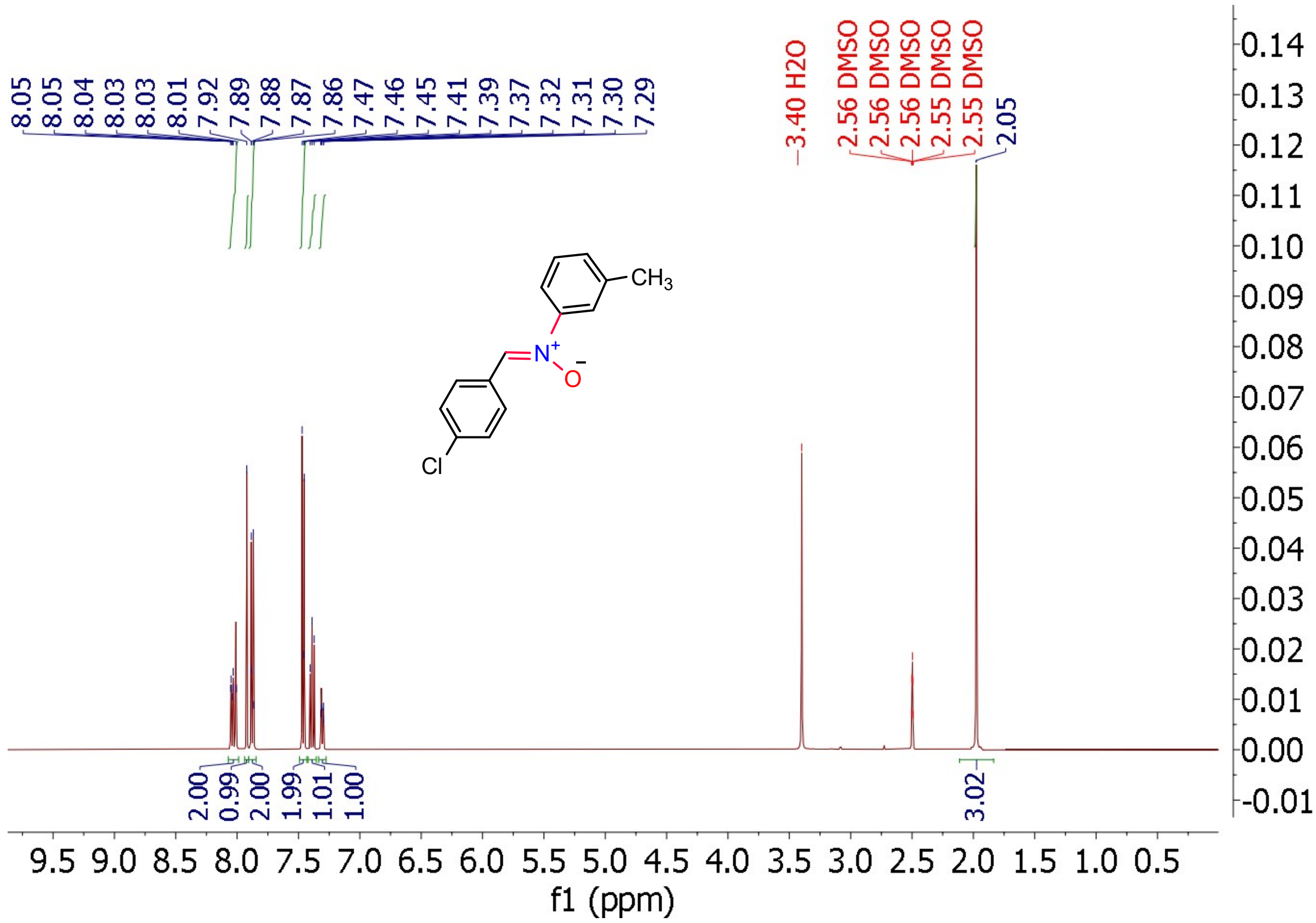

Figure 7: ^{1}H-NMR of nitrone (1f)

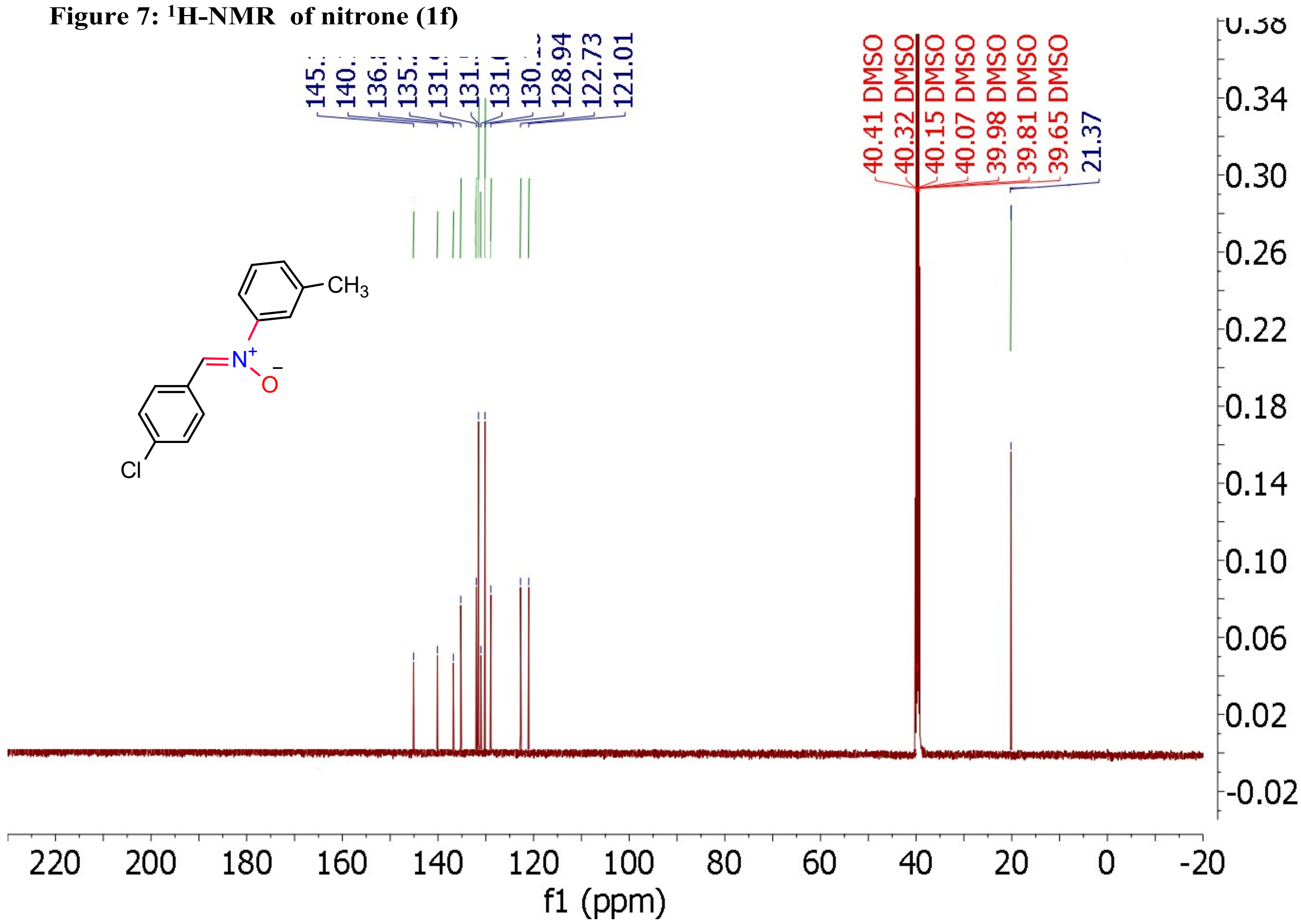

Figure 8: ^{13}C-NMR for nitrone (1f)

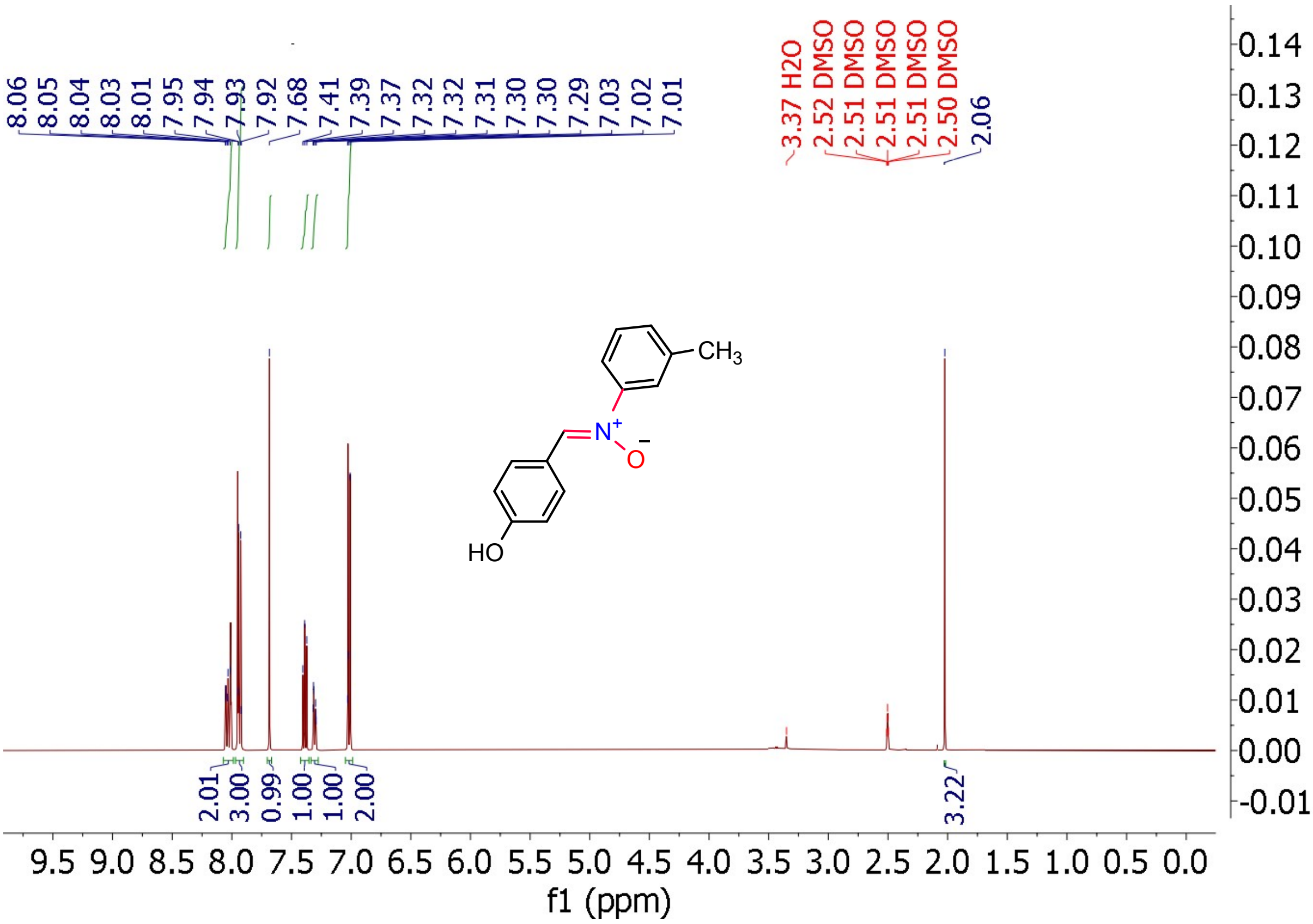

Figure 9: ^{1}H-NMR of nitrone (1h)

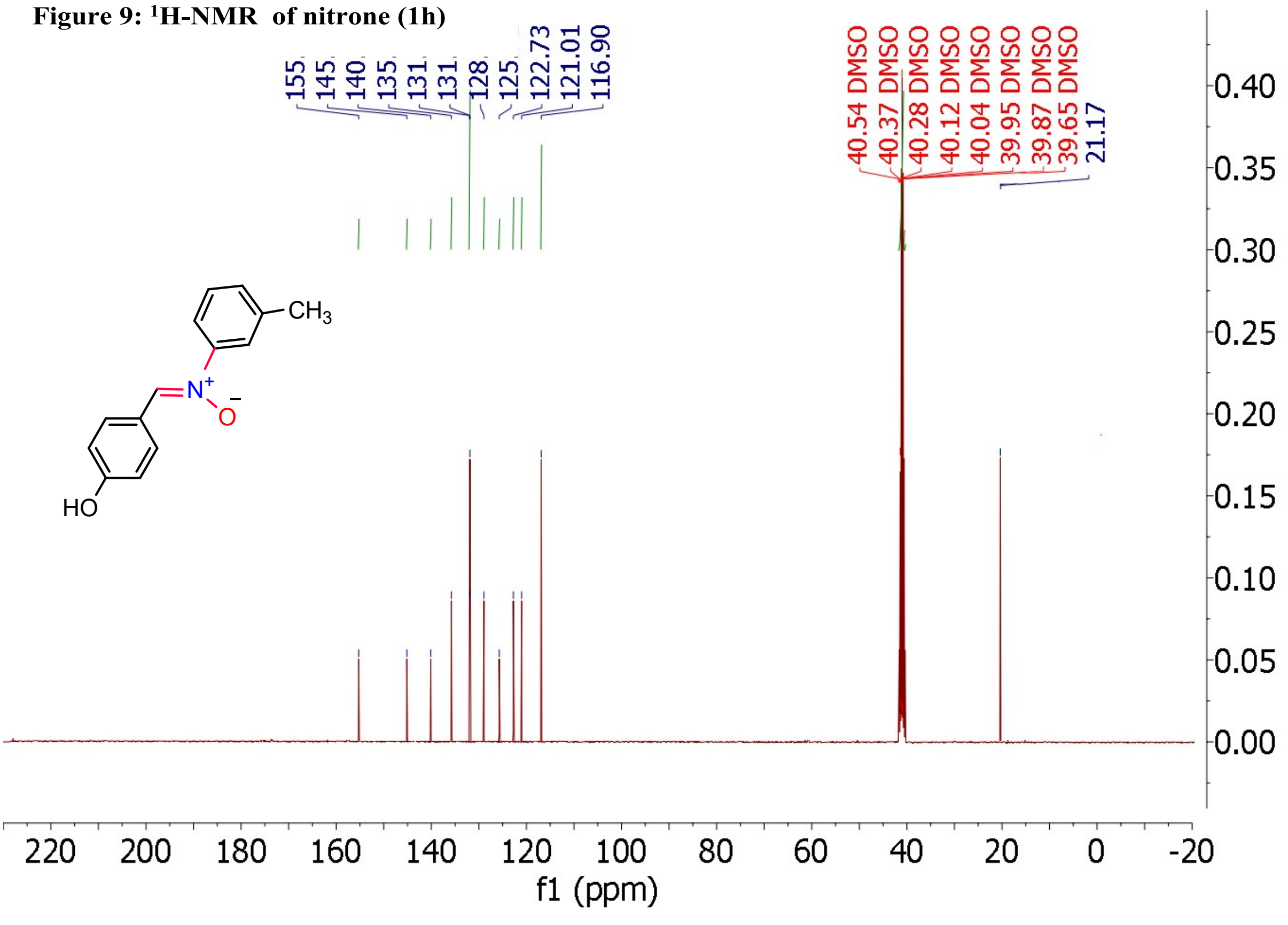

Figure 10: ^{13}C-NMR for nirtone (1h)

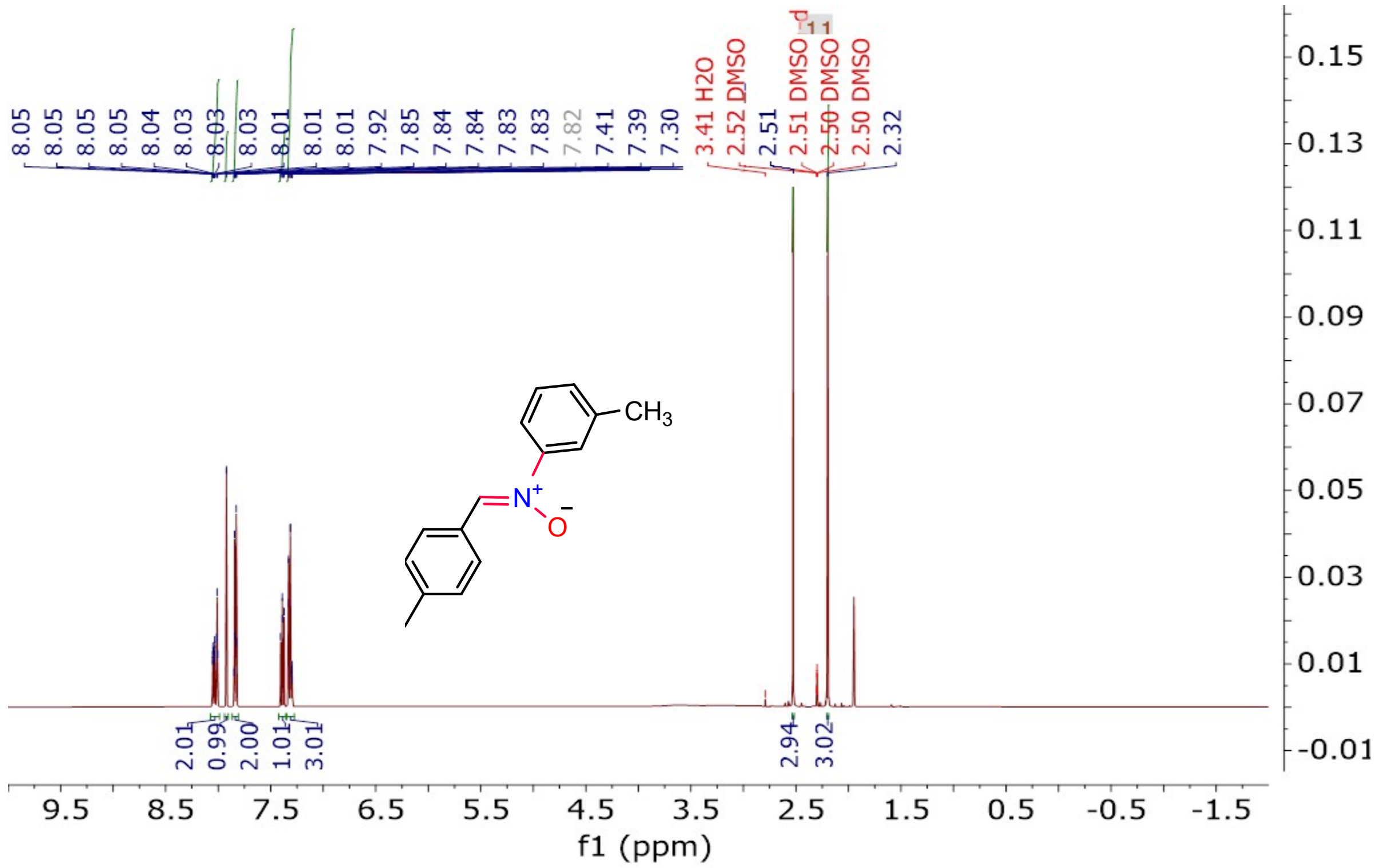

Figure 11: ^{1}H-NMR of nitrone (1l)

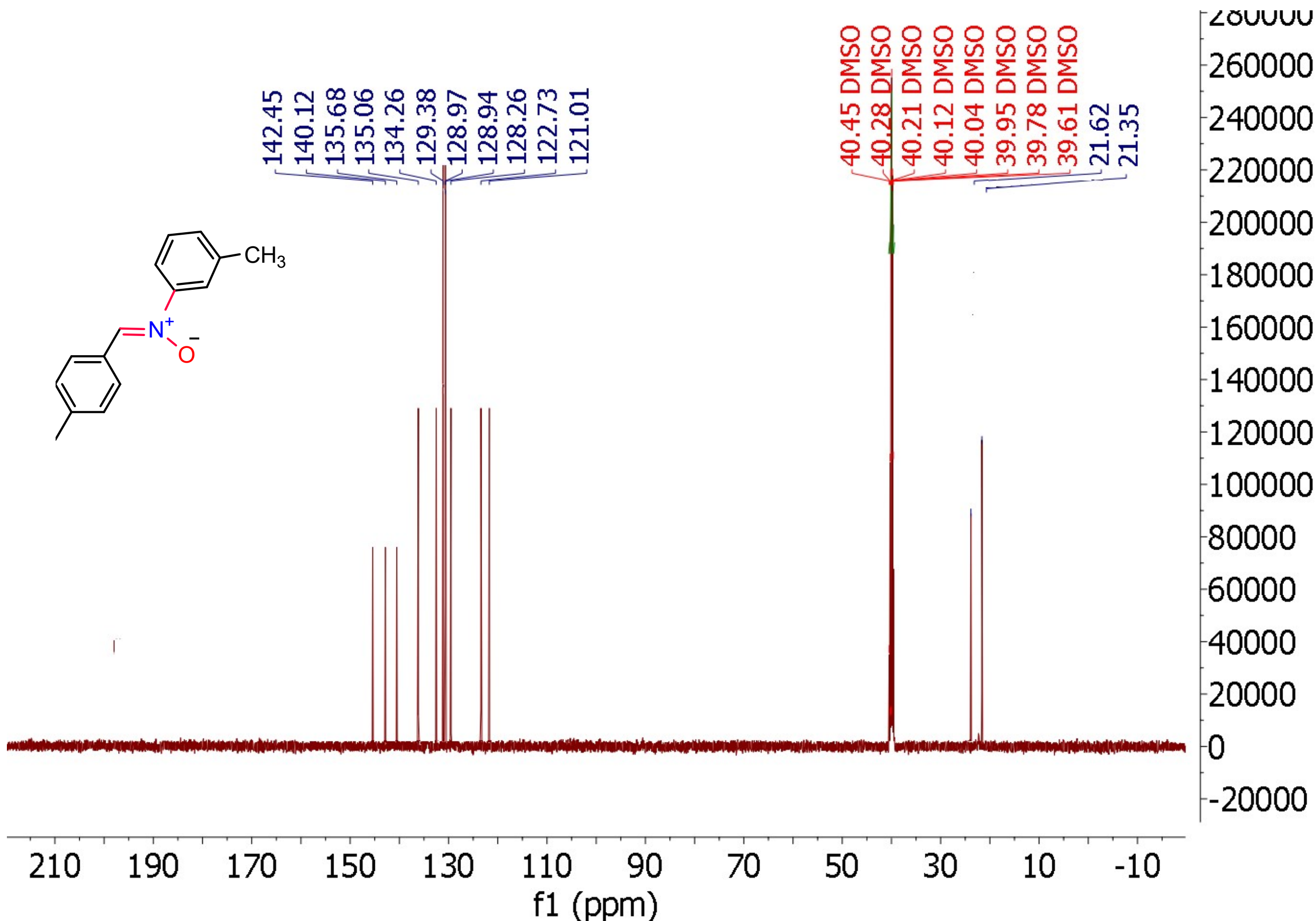

Figure 12: ^{13}C-NMR for nitrone (1l)

¹H–NMR, ¹³C–NMR, LC–MS spectroscopies, and HPLC purity analysis for isoxazolidines

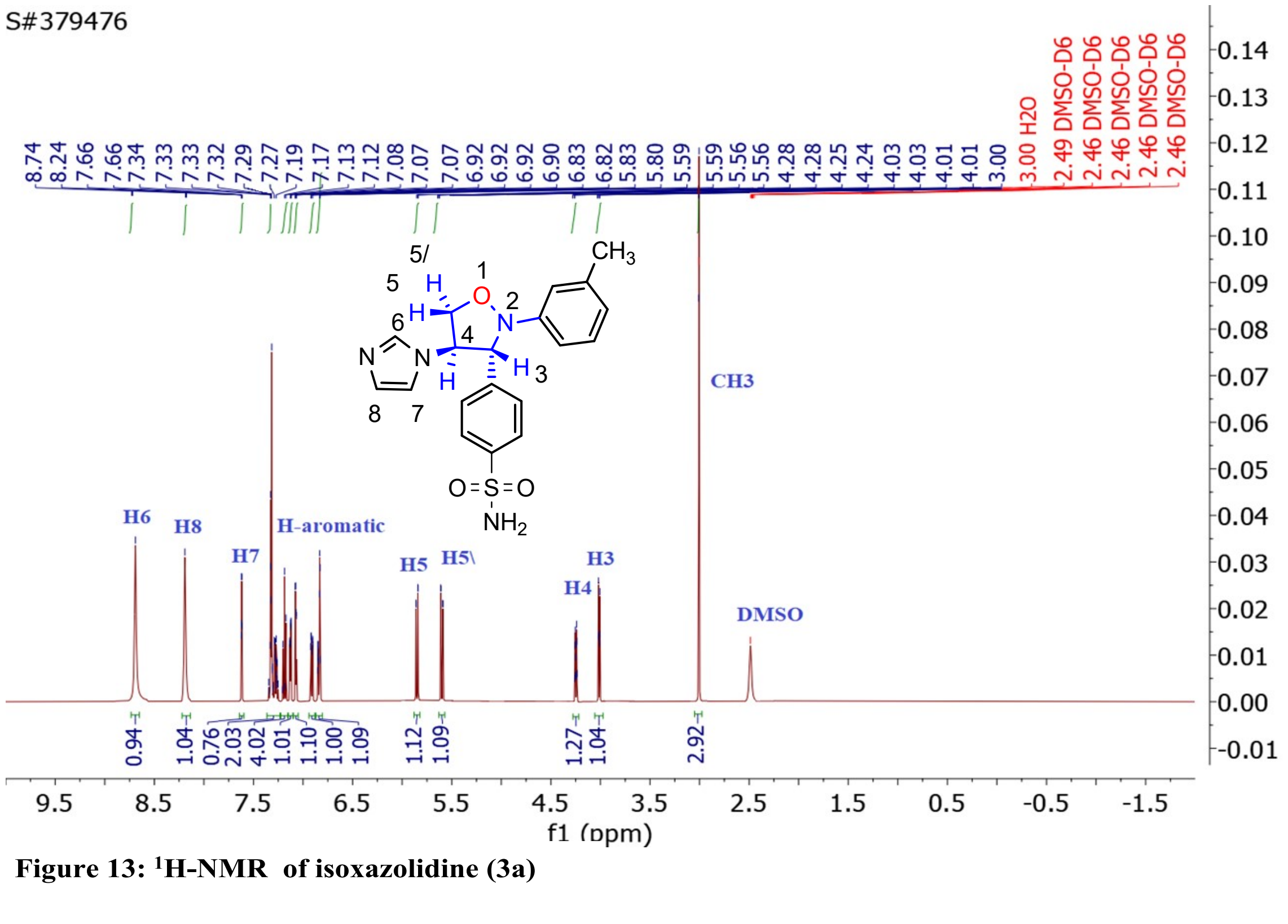

Figure 13: ¹H-NMR of isoxazolidine (3a)

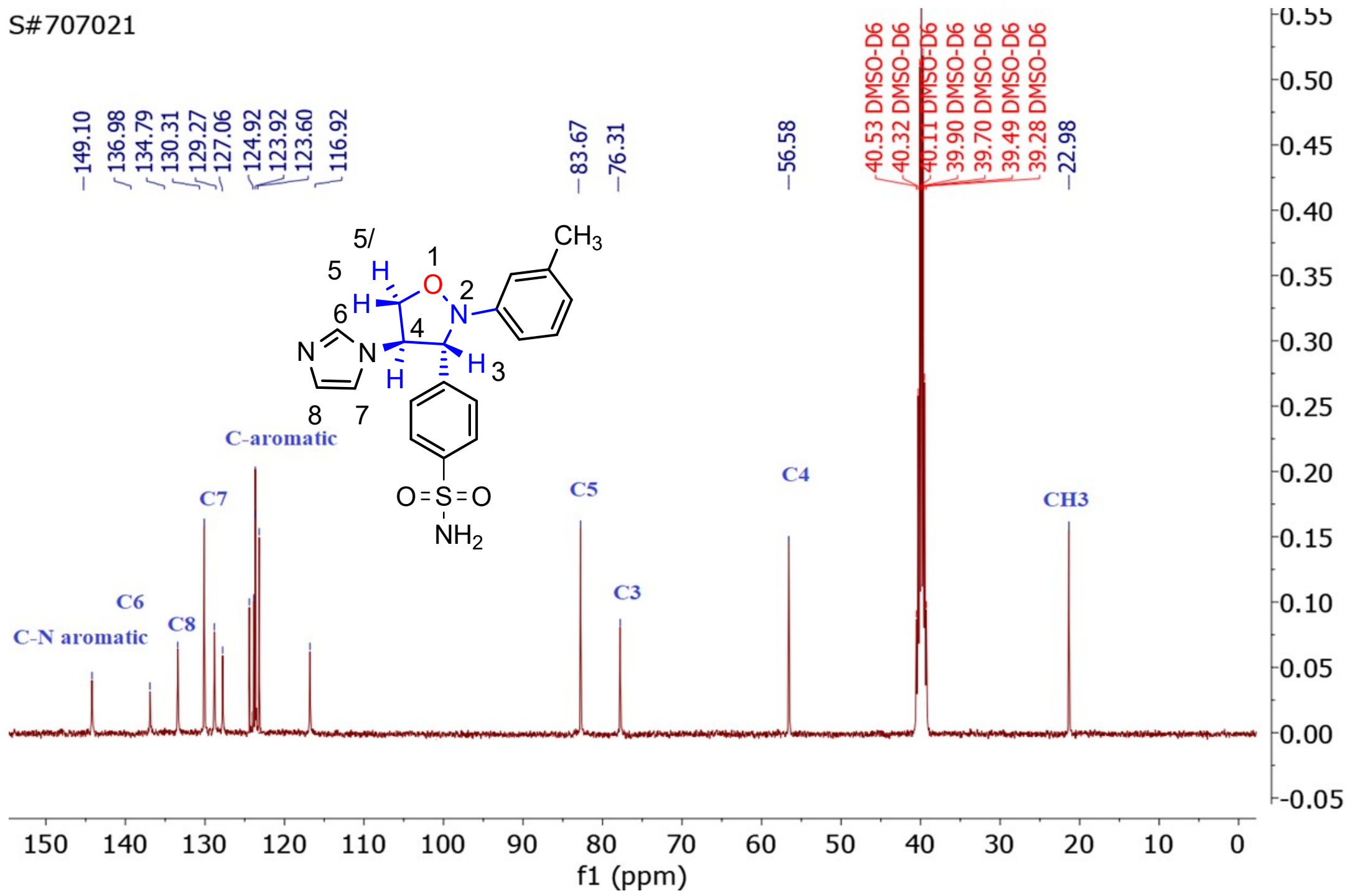

Figure 14: ^{13}C-NMR for isoxazolidine (3a)

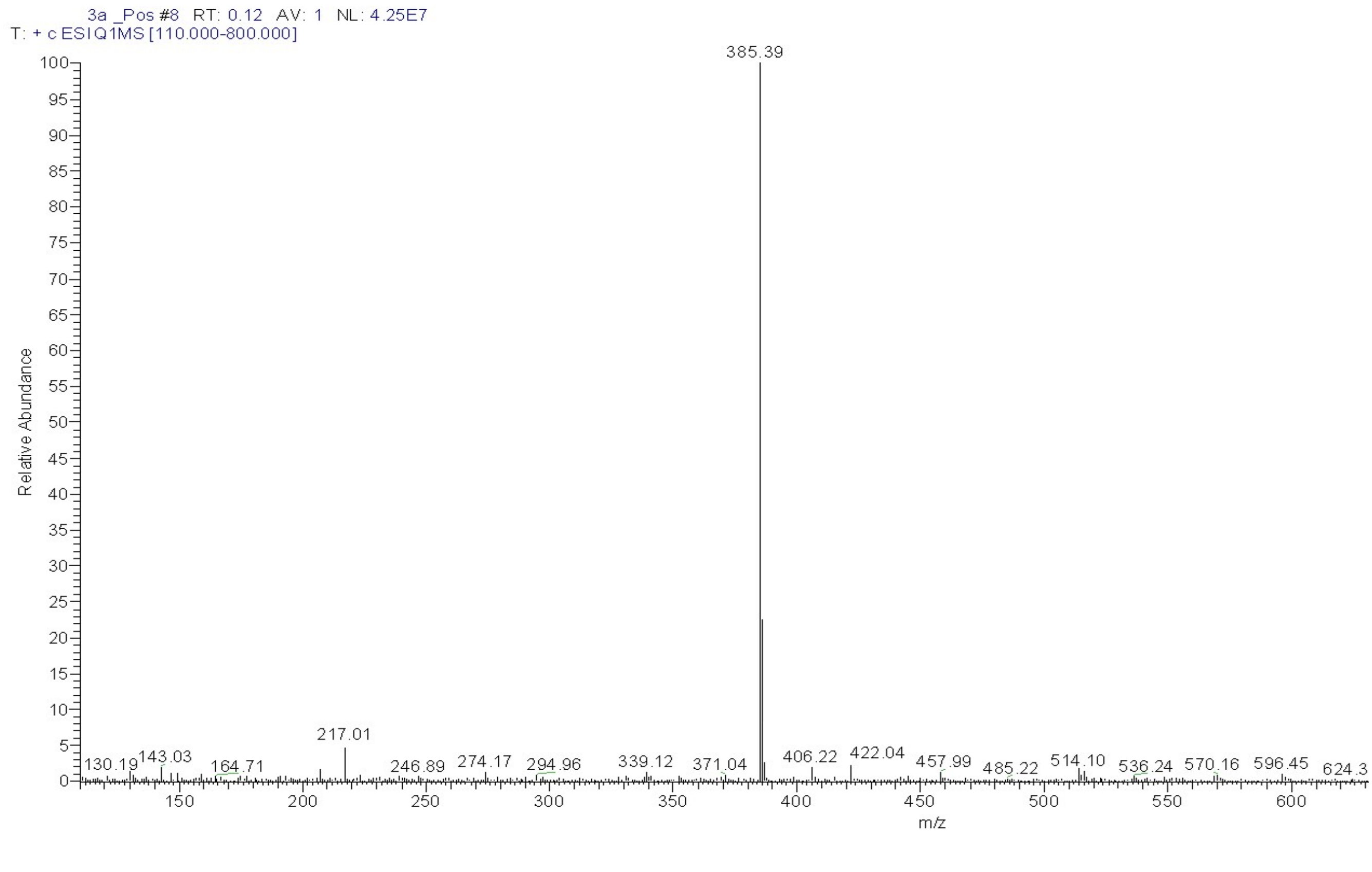

Figure 15: LC-MS for isoxazolidine (3a)

#	Time	Area	Height	Width	Area%	Symmetry
1	15.257	4170.8	774.4	0.0898	98.070	0.631

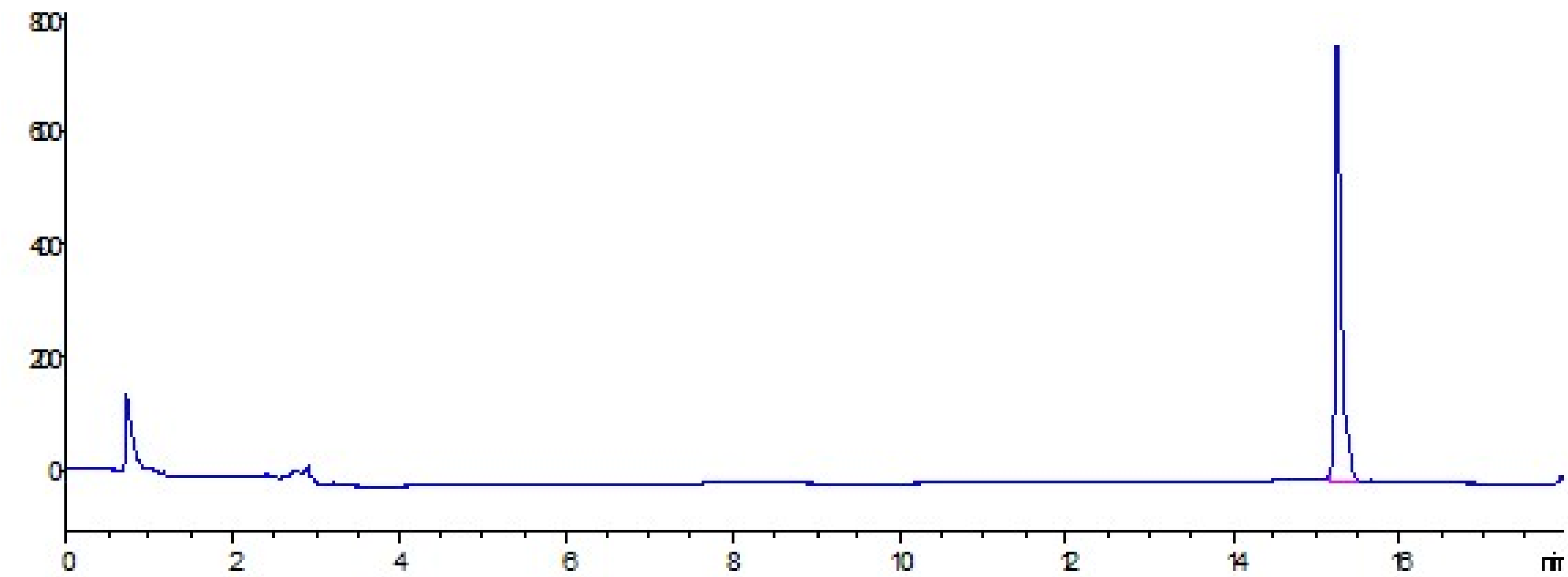

Figure 16: HPLC purity analysis of isoxazolidine (3a)

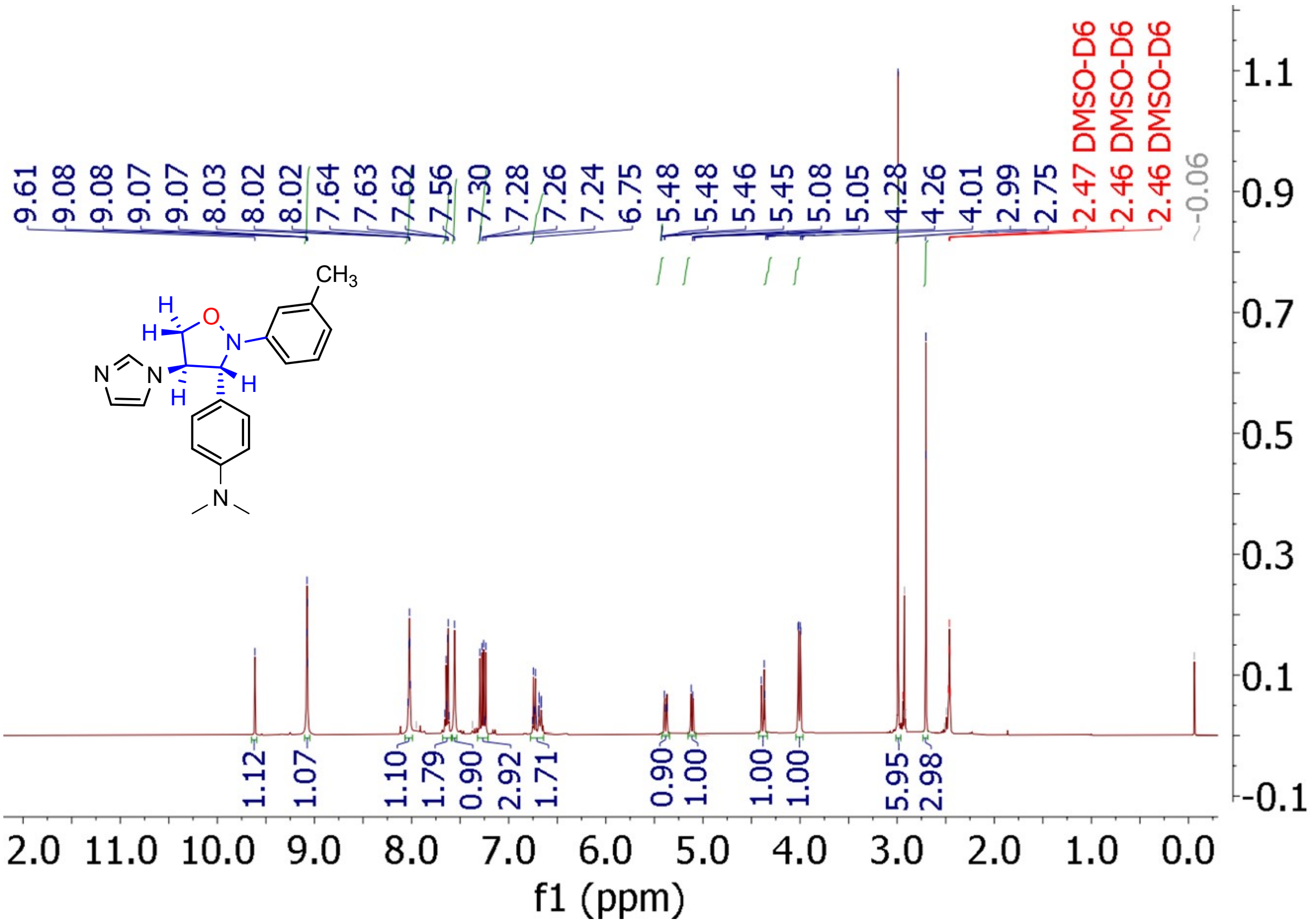

Figure 17: ^{1}H-NMR of isoxazolidine (3b)

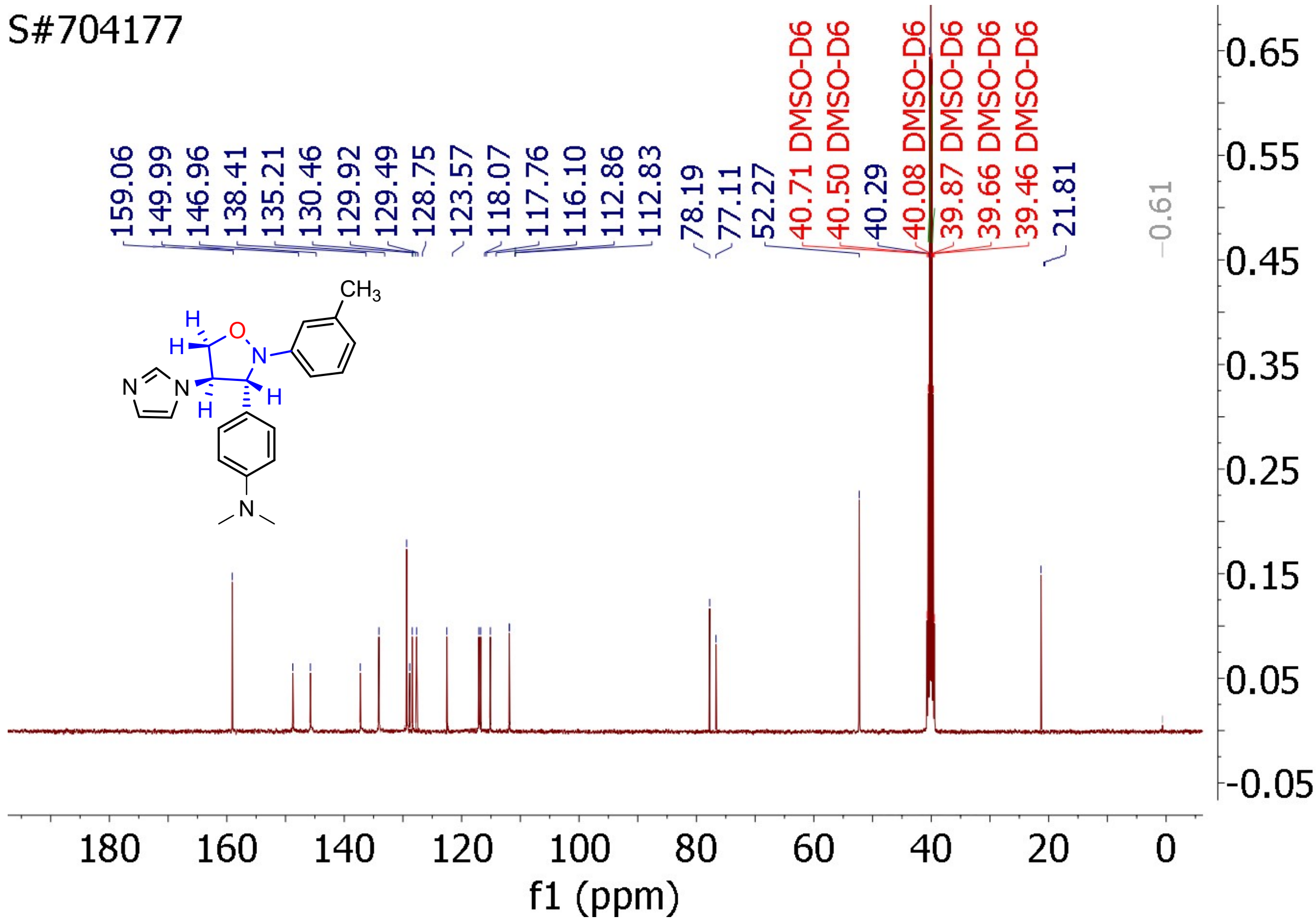

Figure 18: ^{13}C-NMR for isoxazolidine (3b)

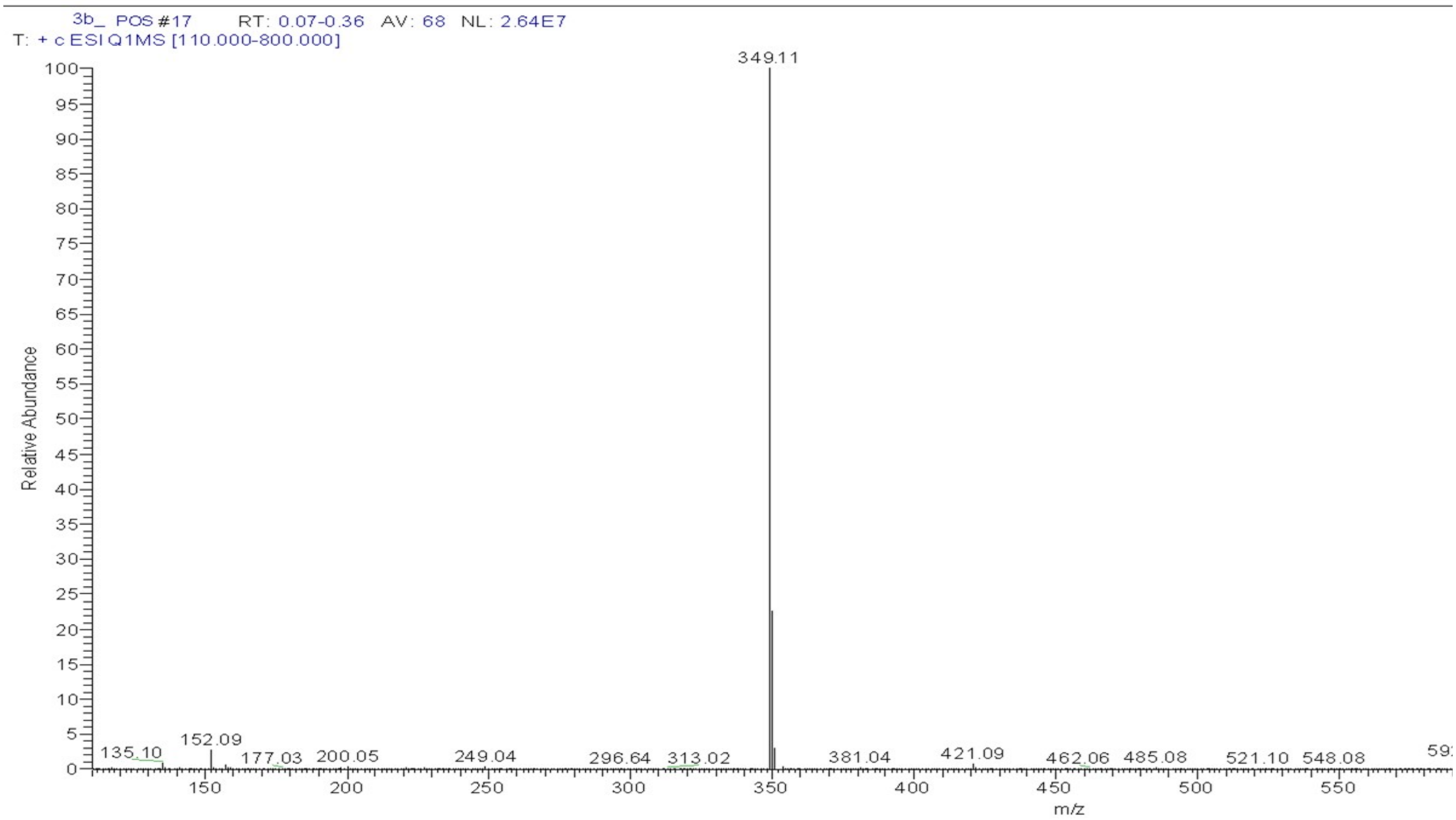

Figure 19: LC-MS for isoxazolidine (3b)

#	Time	Area	Height	Width	Area%	Symmetry
1	11.294	11774.9	2426.5	0.0809	98.871	0.647
2	15.254	134.5	30	0.0747	1.129	0.807

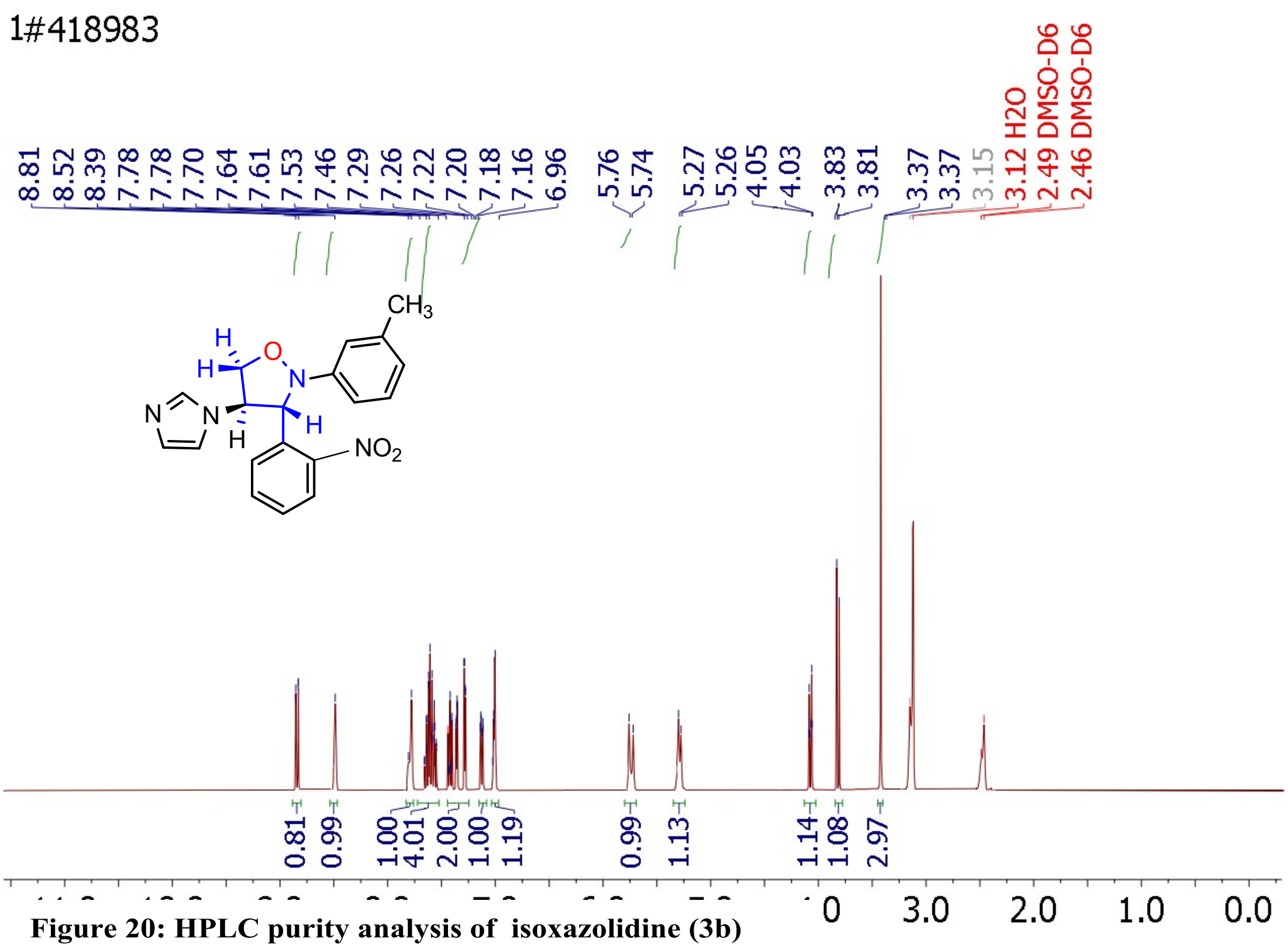

Figure 20: HPLC purity analysis of isoxazolidine (3b)

Figure 21: ^{1}H-NMR of isoxazolidine (3c)

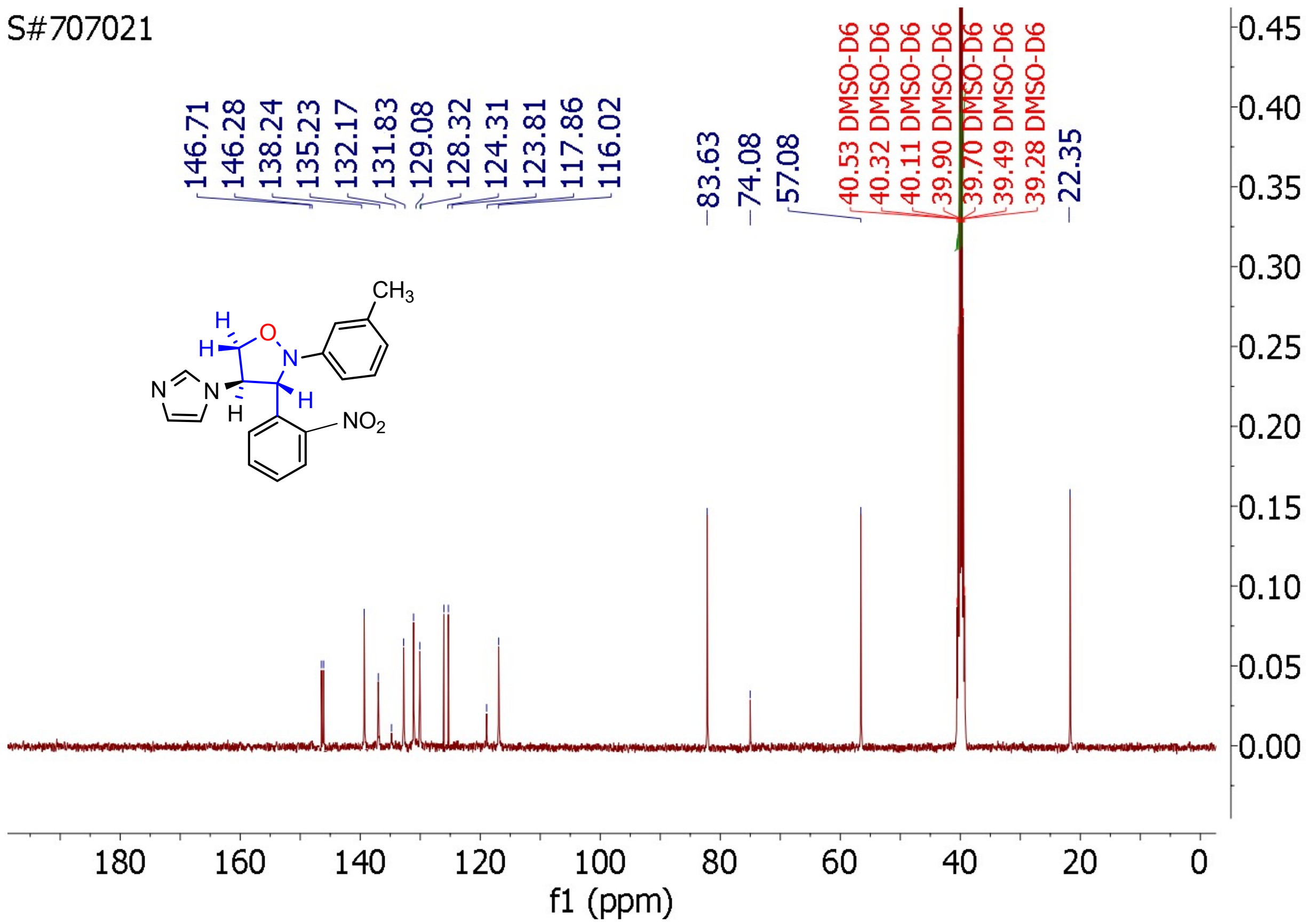

Figure 22: ^{13}C-NMR for isoxazolidine (3c)

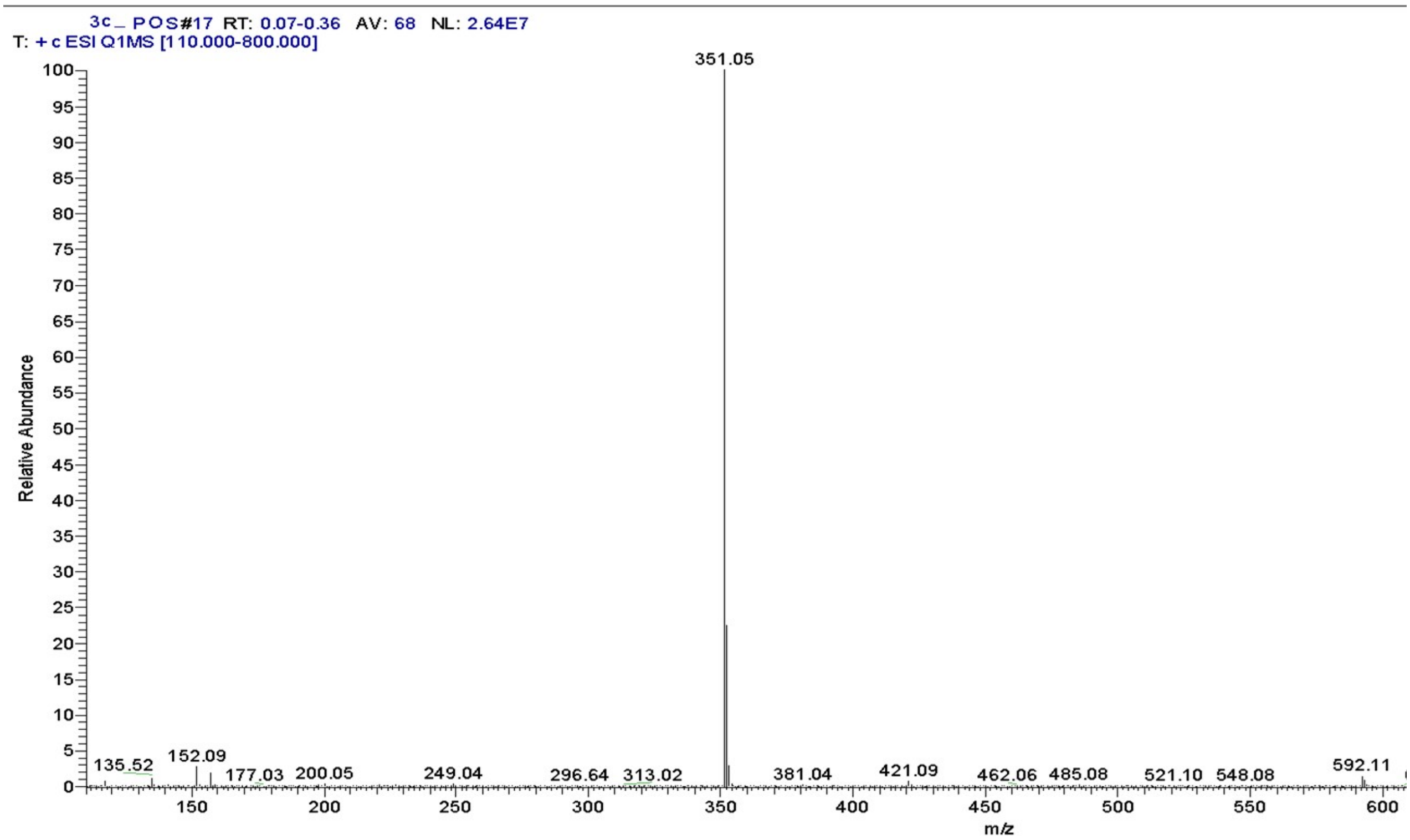

Figure 23: LC-MS for isoxazolidine (3c)

#	Time	Area	Height	Width	Area%	Symmetry
1	15.324	1496.5	263.3	0.0947	96.880	0.61

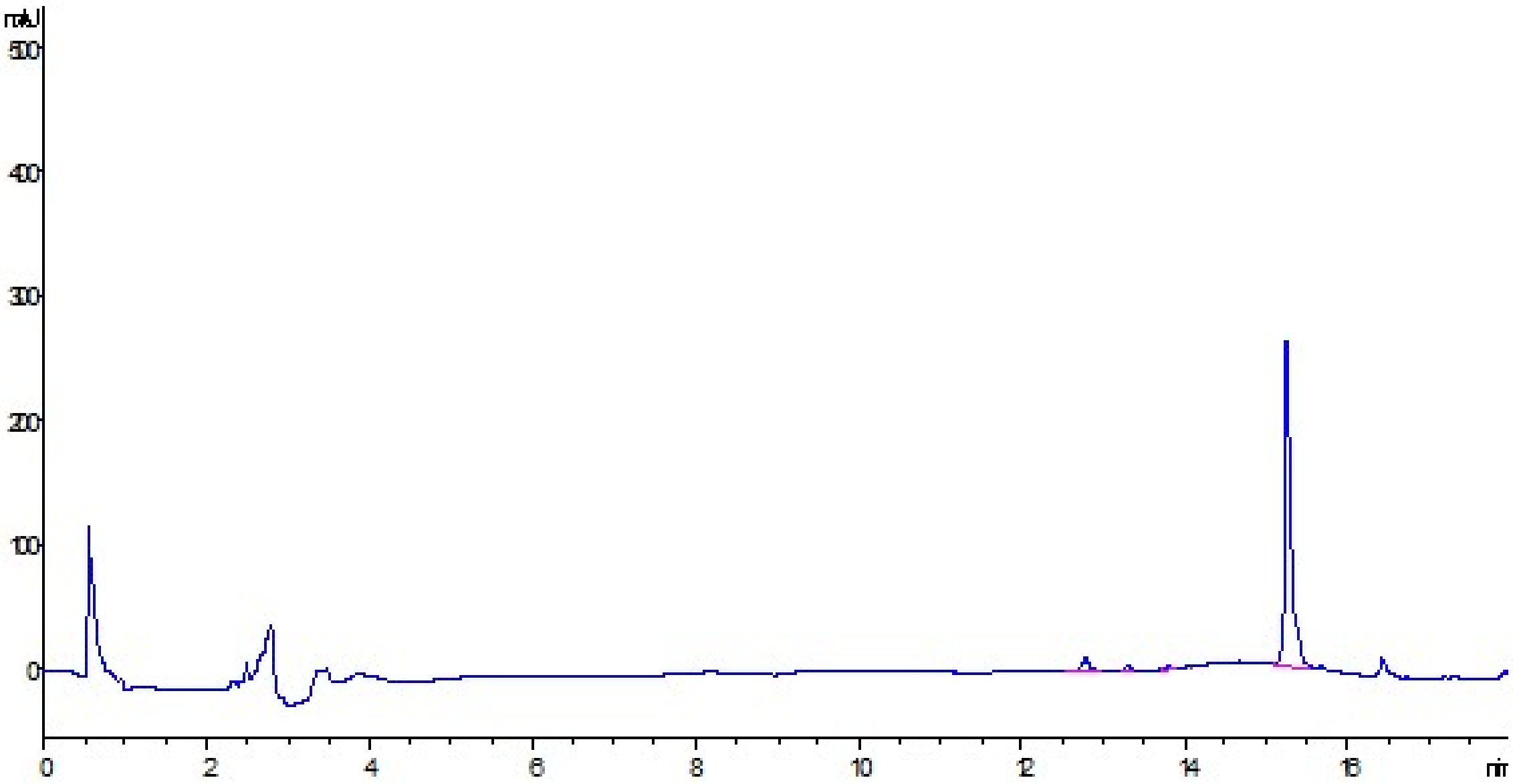

Figure 24: HPLC purity analysis of isoxazolidine (3c)

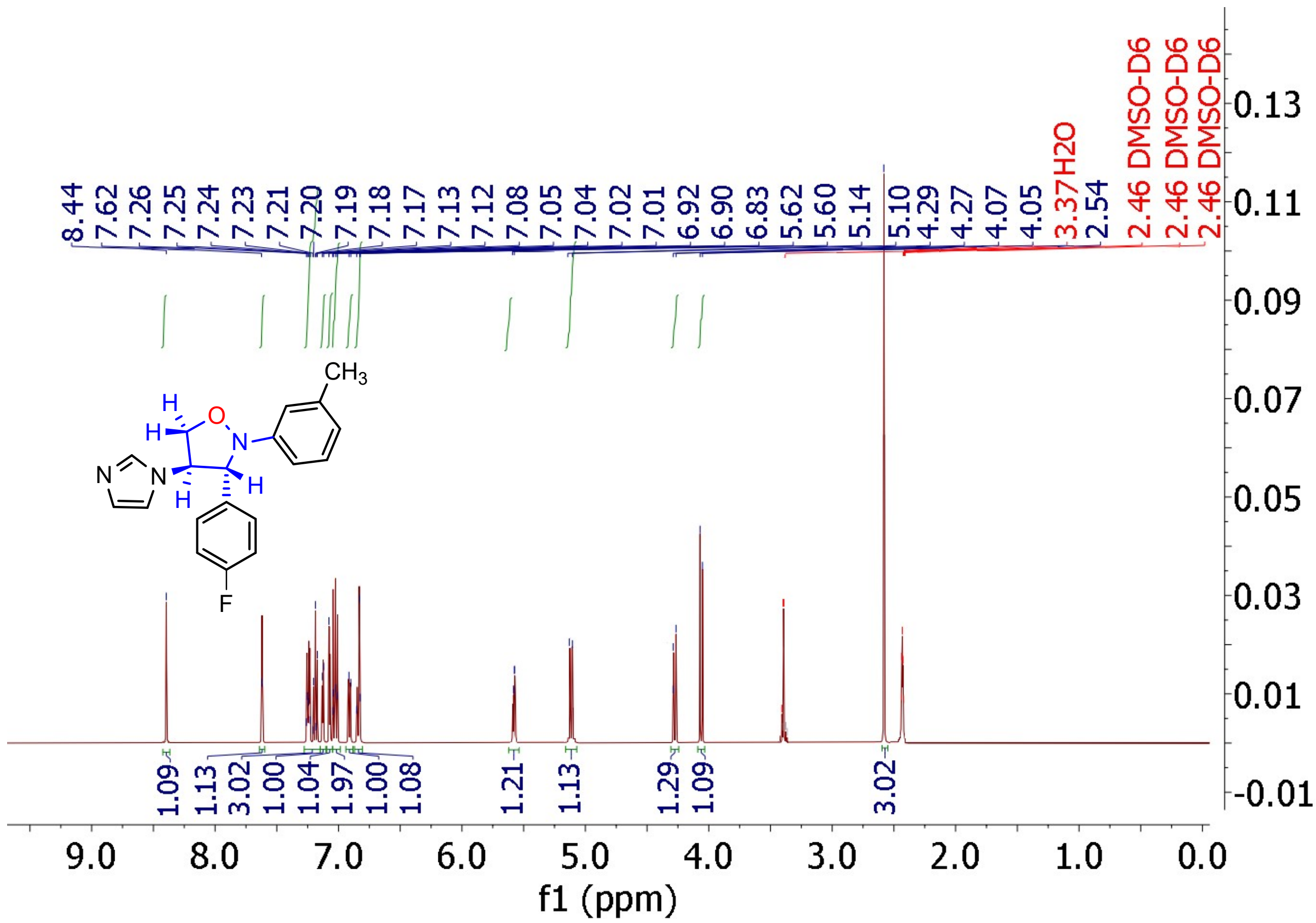

Figure 25: ^{1}H-NMR of isoxazolidine (3d)

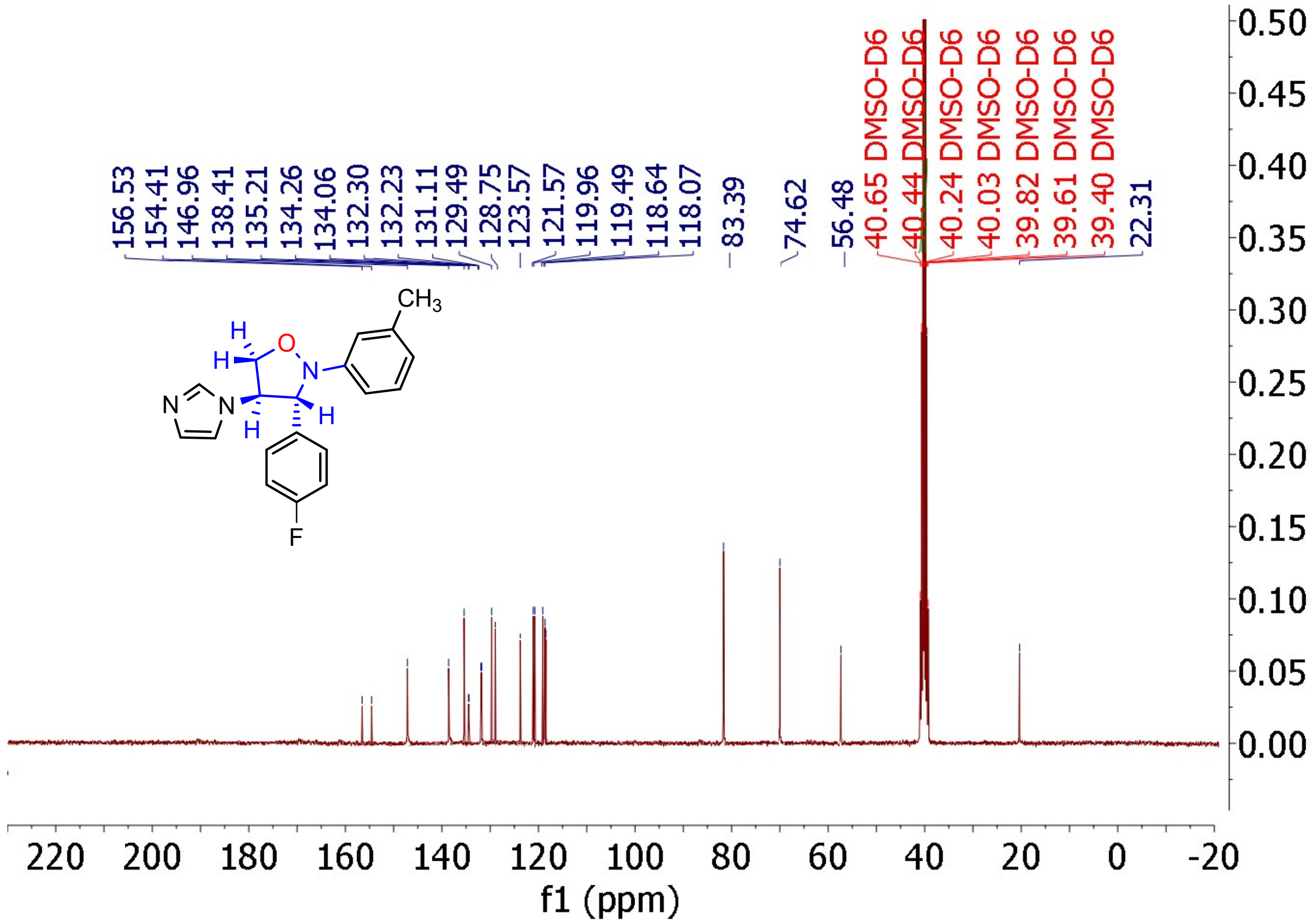

Figure 26: ^{13}C-NMR for isoxazolidine (3d)

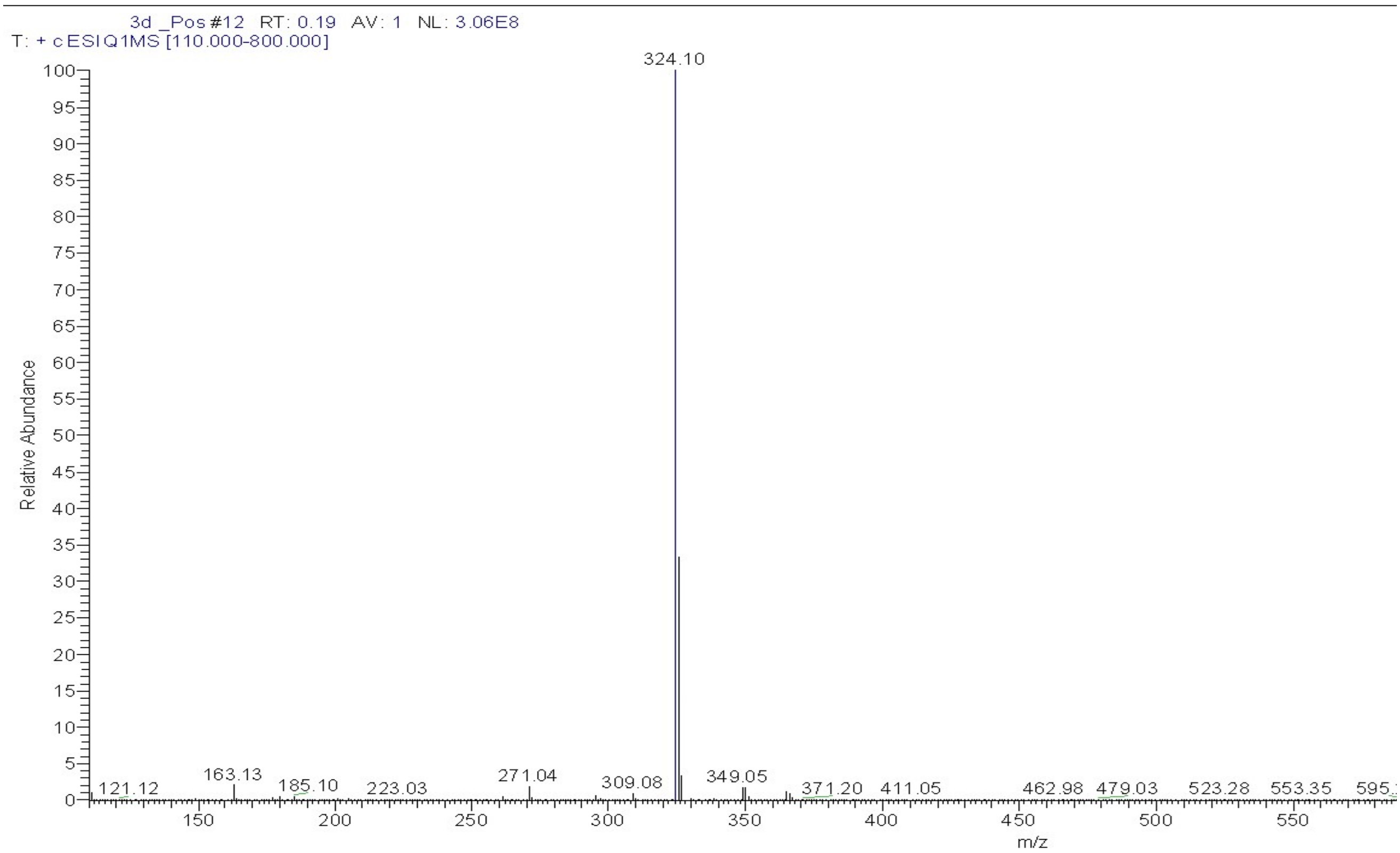

Figure 27: LC-MS for isoxazolidine (3d)

#	Time	Area	Height	Width	Area%	Symmetry
1	12.017	4466.4	921.7	0.0808	95.881	0.644

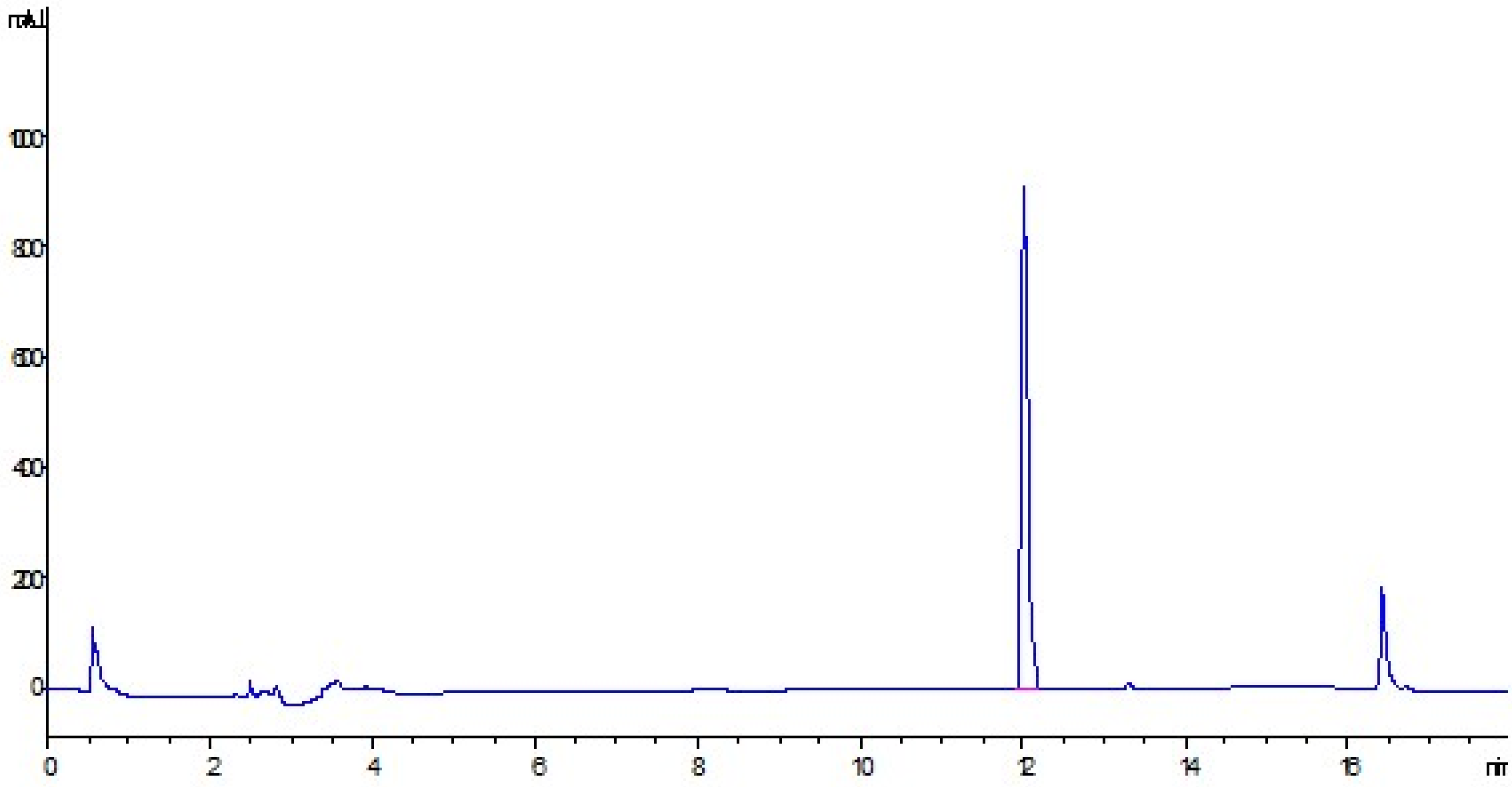

Figure 28: HPLC purity analysis of isoxazolidine (3d)

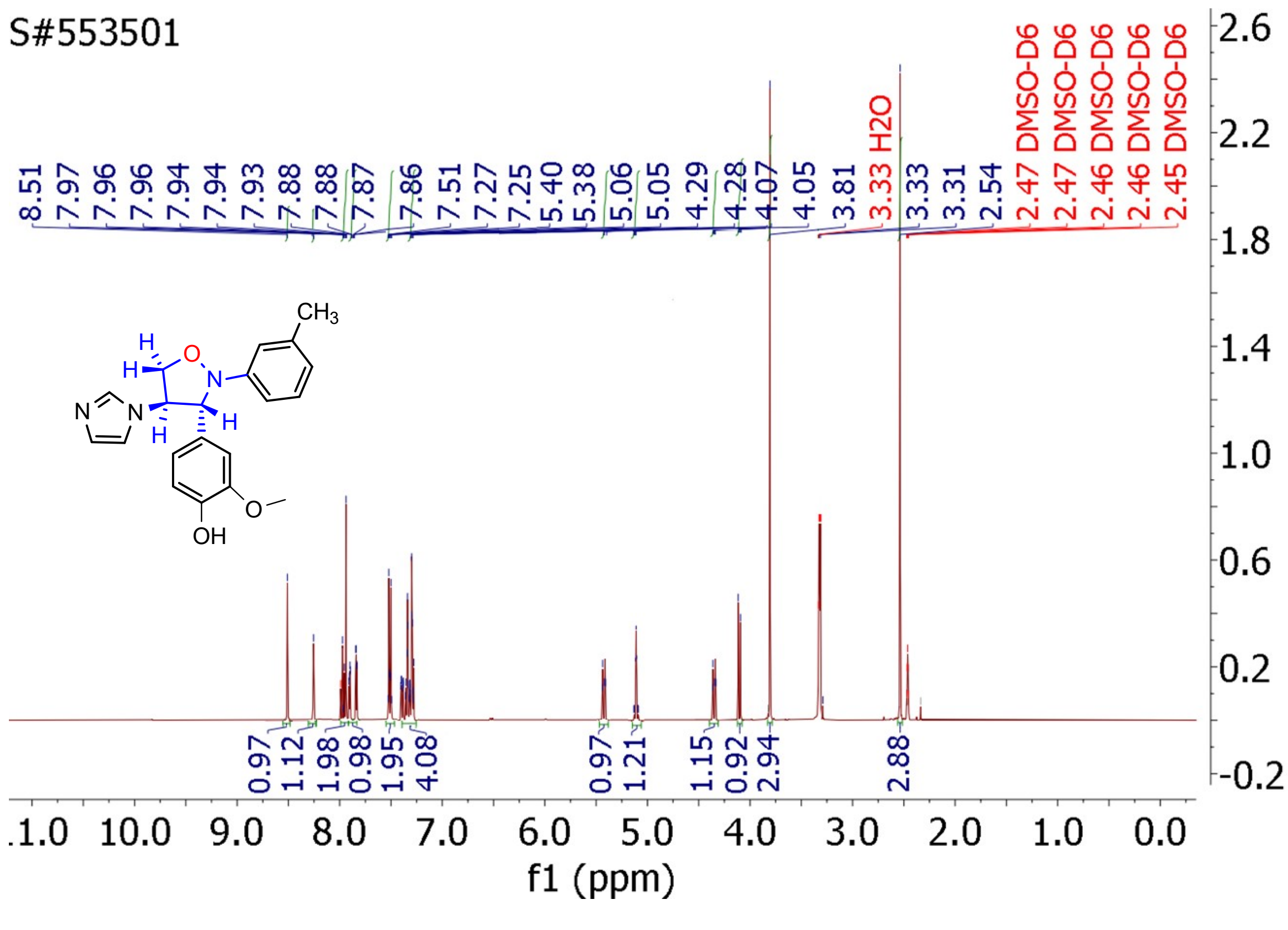

Figure 29: ^{1}H-NMR of isoxazolidine (3e)

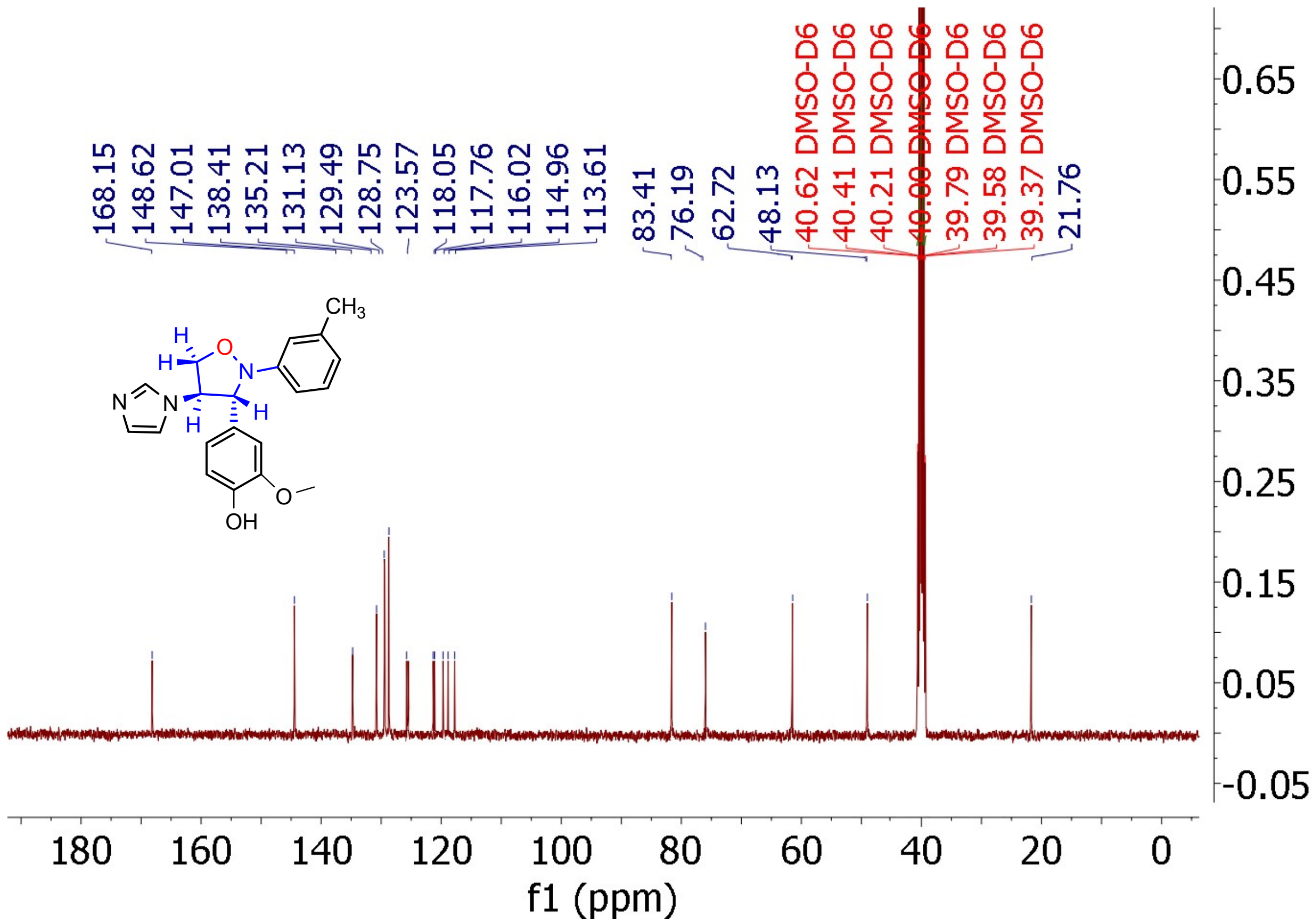

Figure 30: ^{13}C-NMR for isoxazolidine (3e)

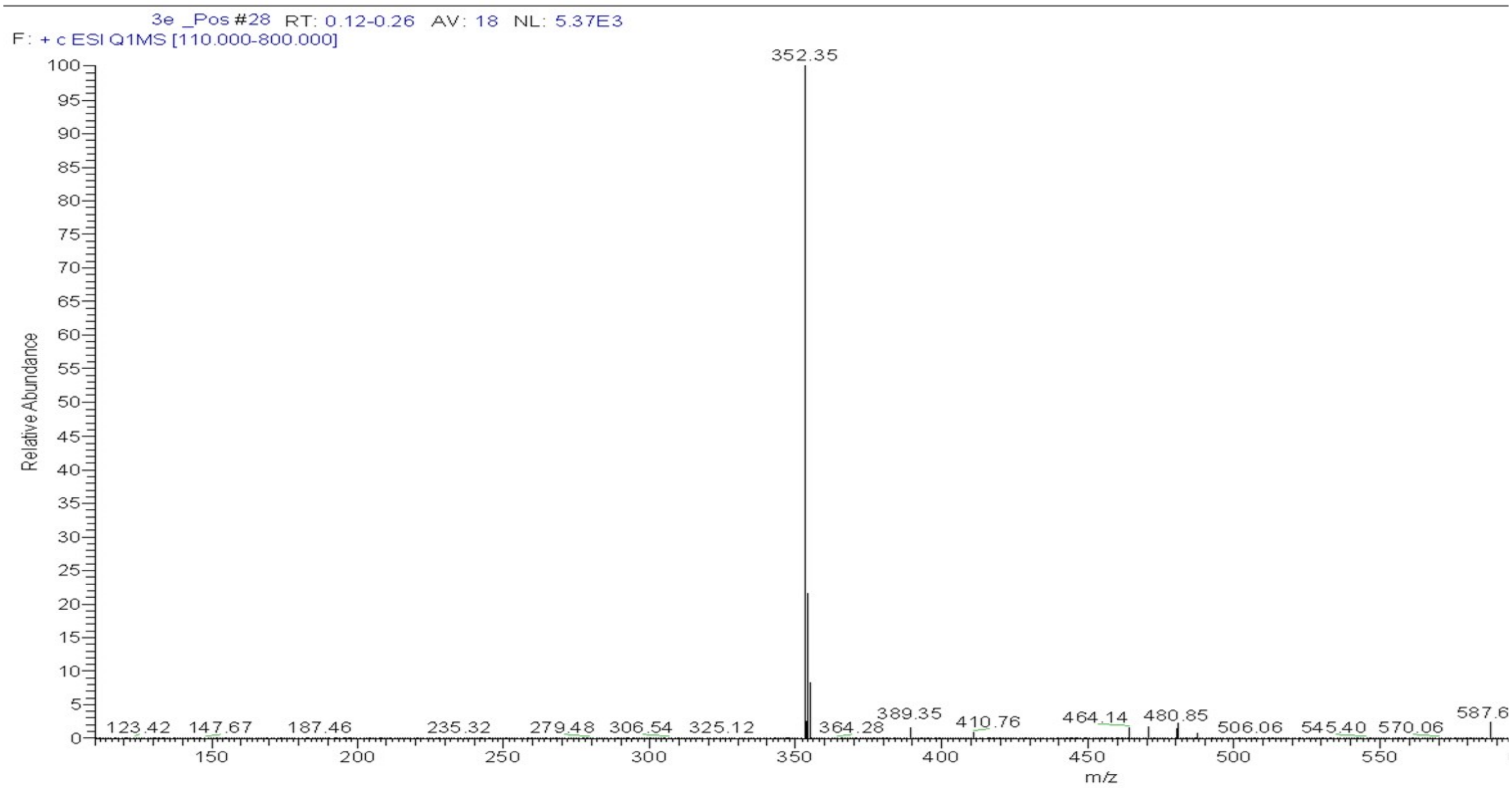

Figure 31: LC-MS for isoxazolidine (3e)

#	Time	Area	Height	Width	Area%	Symmetry
1	15.258	1807.5	337.4	0.0893	98.854	0.636

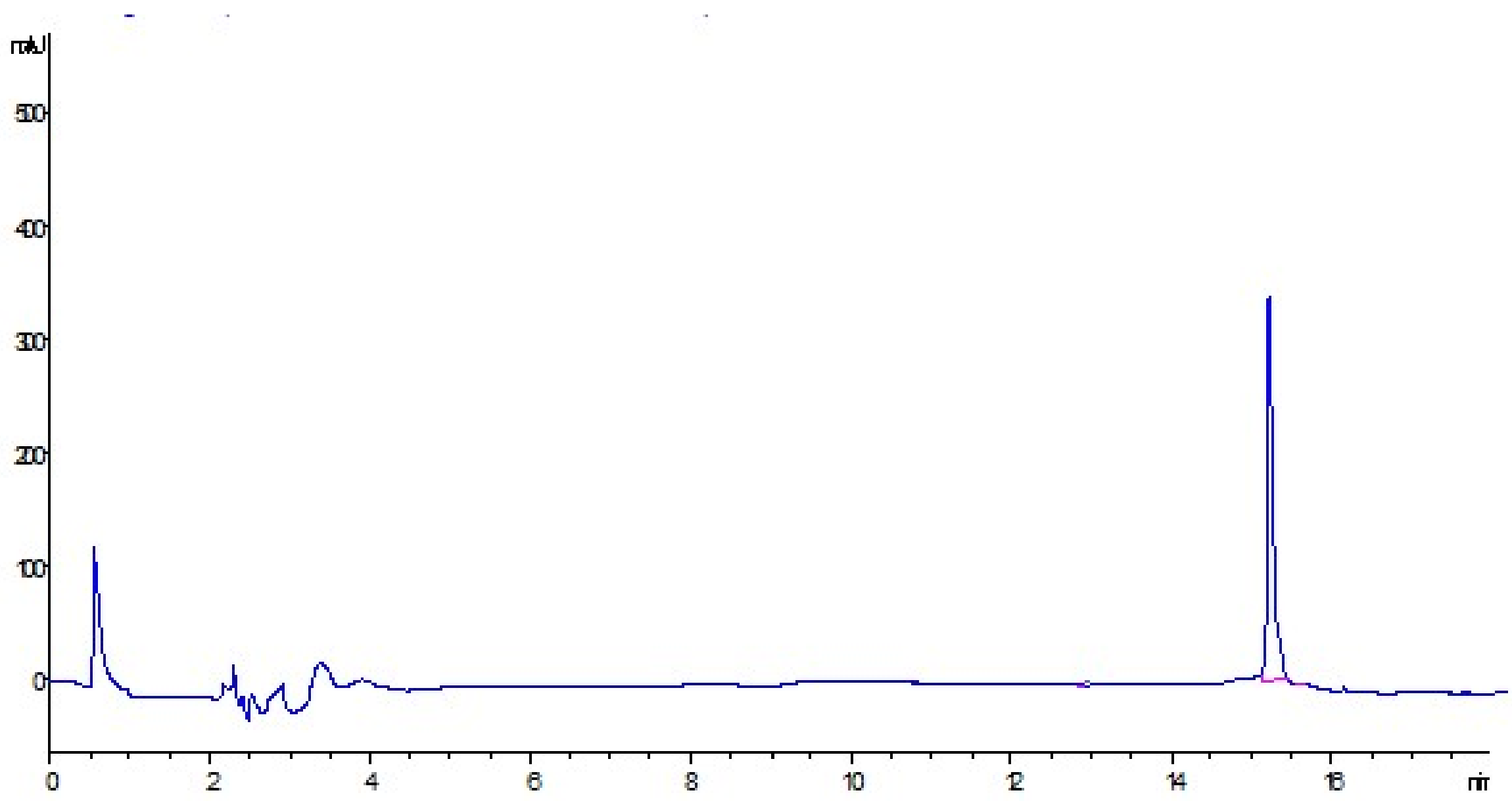

Figure 32: HPLC purity analysis of isoxazolidine (3e)

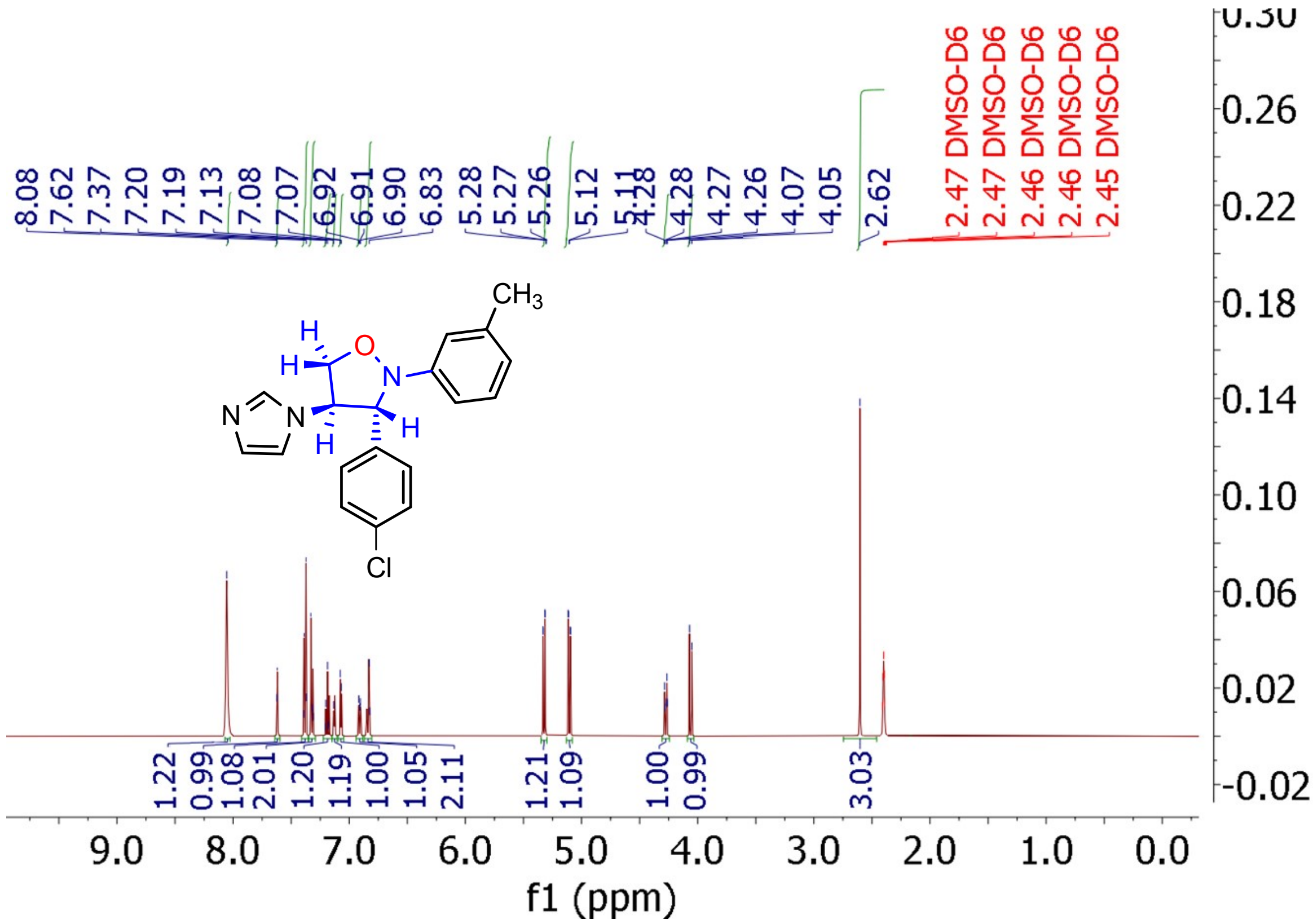

Figure 33: ^{1}H-NMR of isoxazolidine (3f)

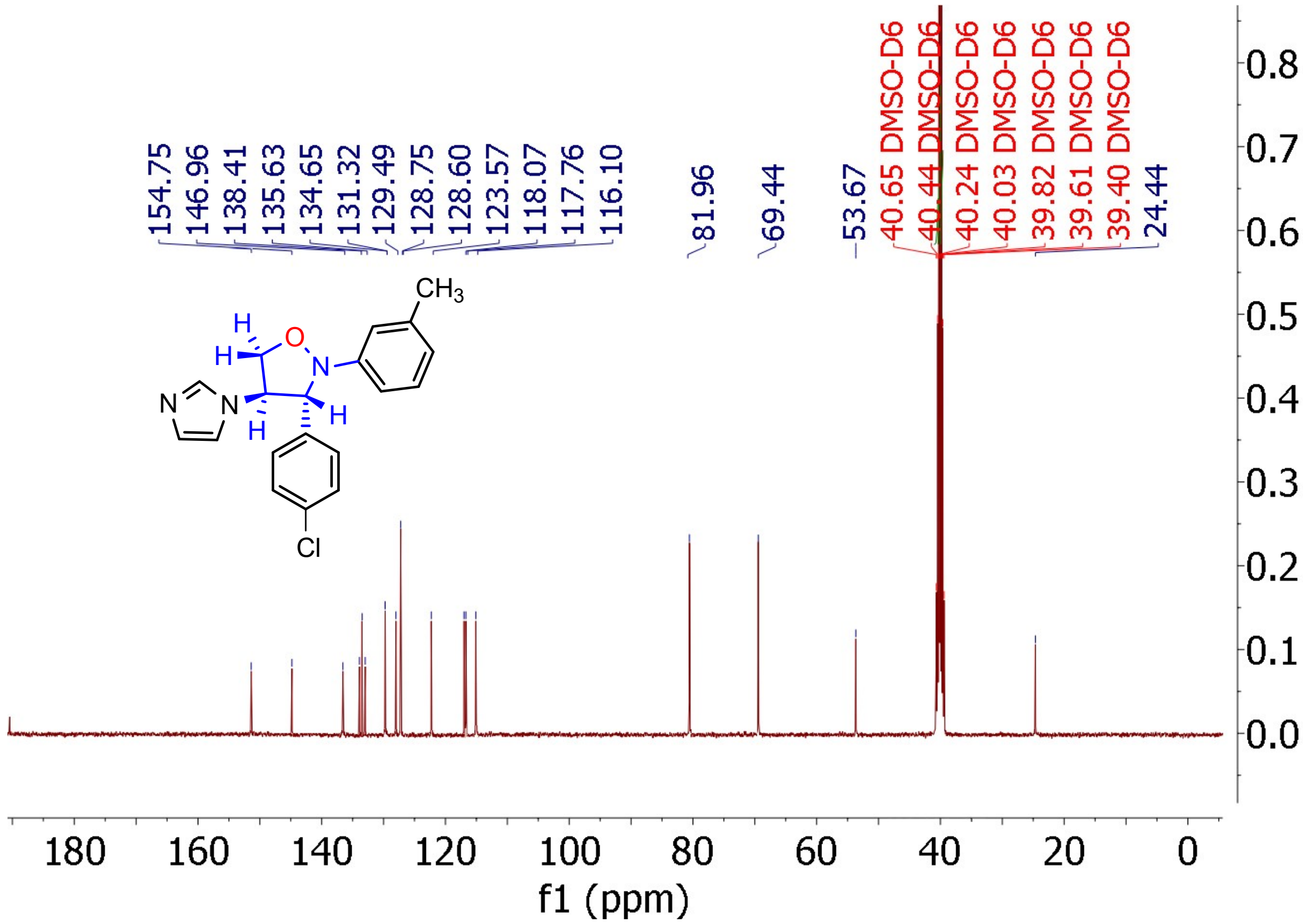

Figure 34: ^{13}C-NMR for isoxazolidine (3f)

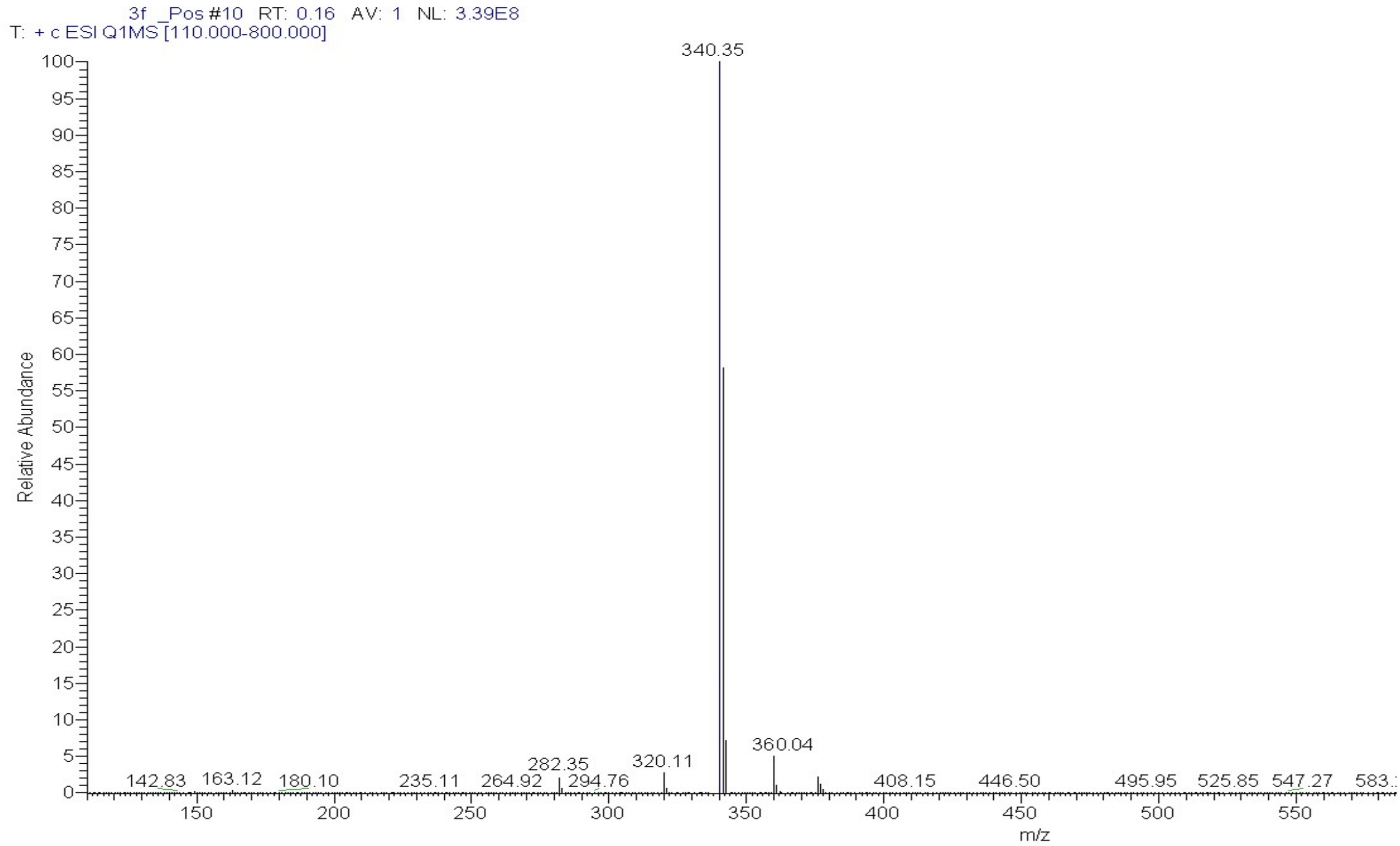

Figure 35: LC-MS for isoxazolidine (3f)

#	Time	Area	Height	Width	Area%	Symmetry
1	11.114	11871.5	2426.5	0.0809	98.871	0.647
2	15.119	144.5	29	0.8717	1.129	0.807

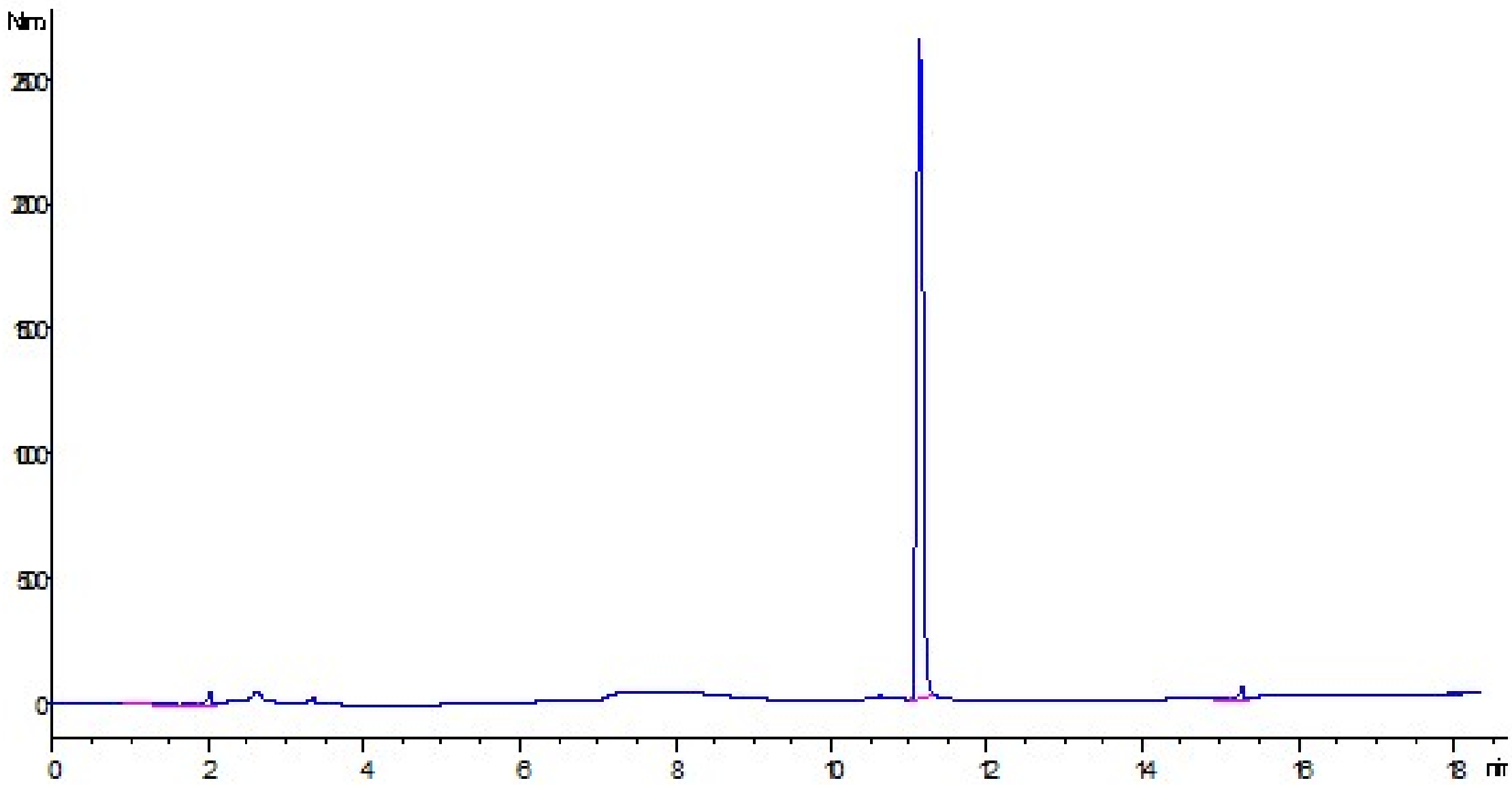

Figure 36: HPLC purity analysis of isoxazolidine (3f)

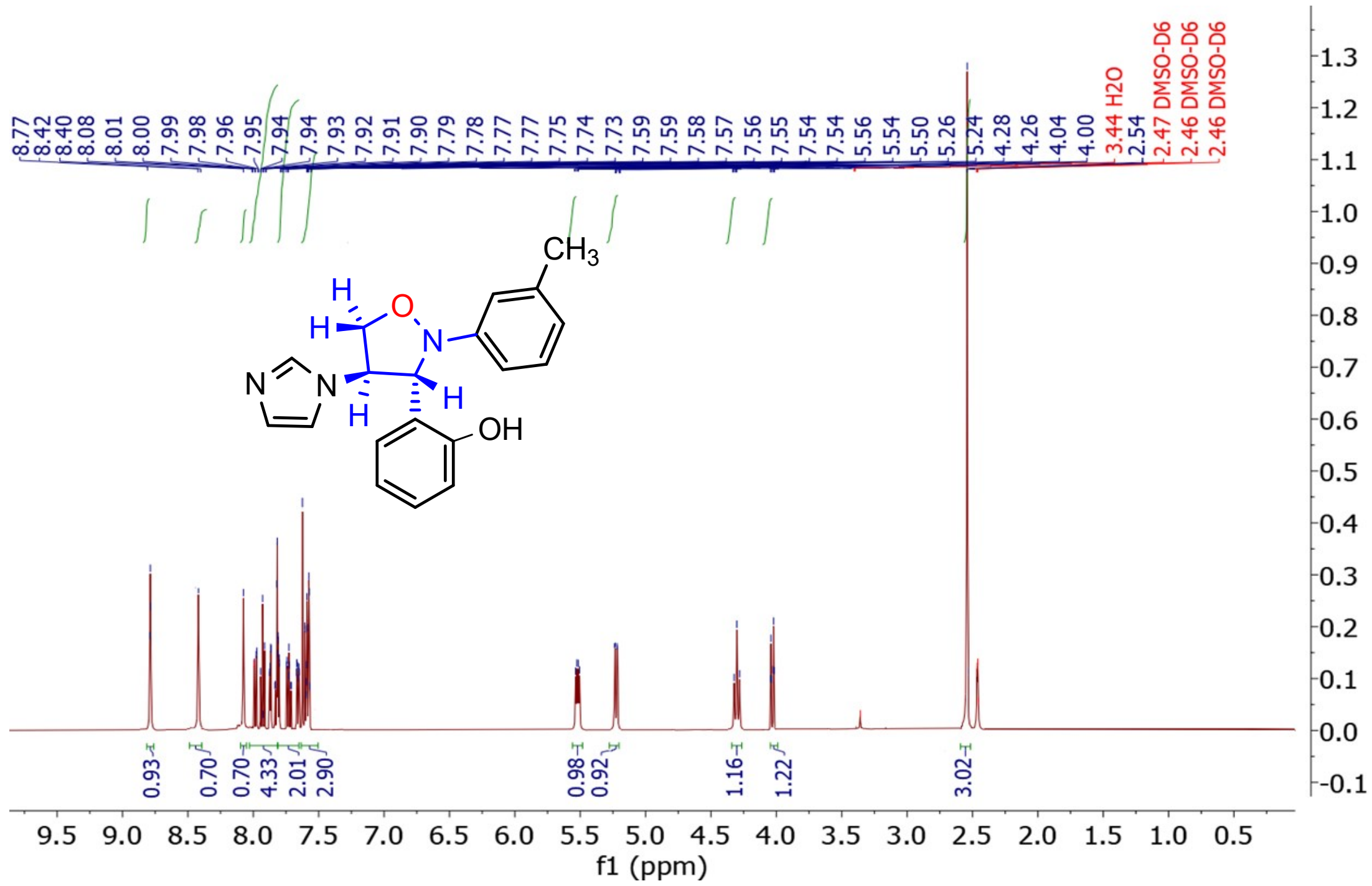

Figure 37: ^{1}H-NMR of isoxazolidine (3g)

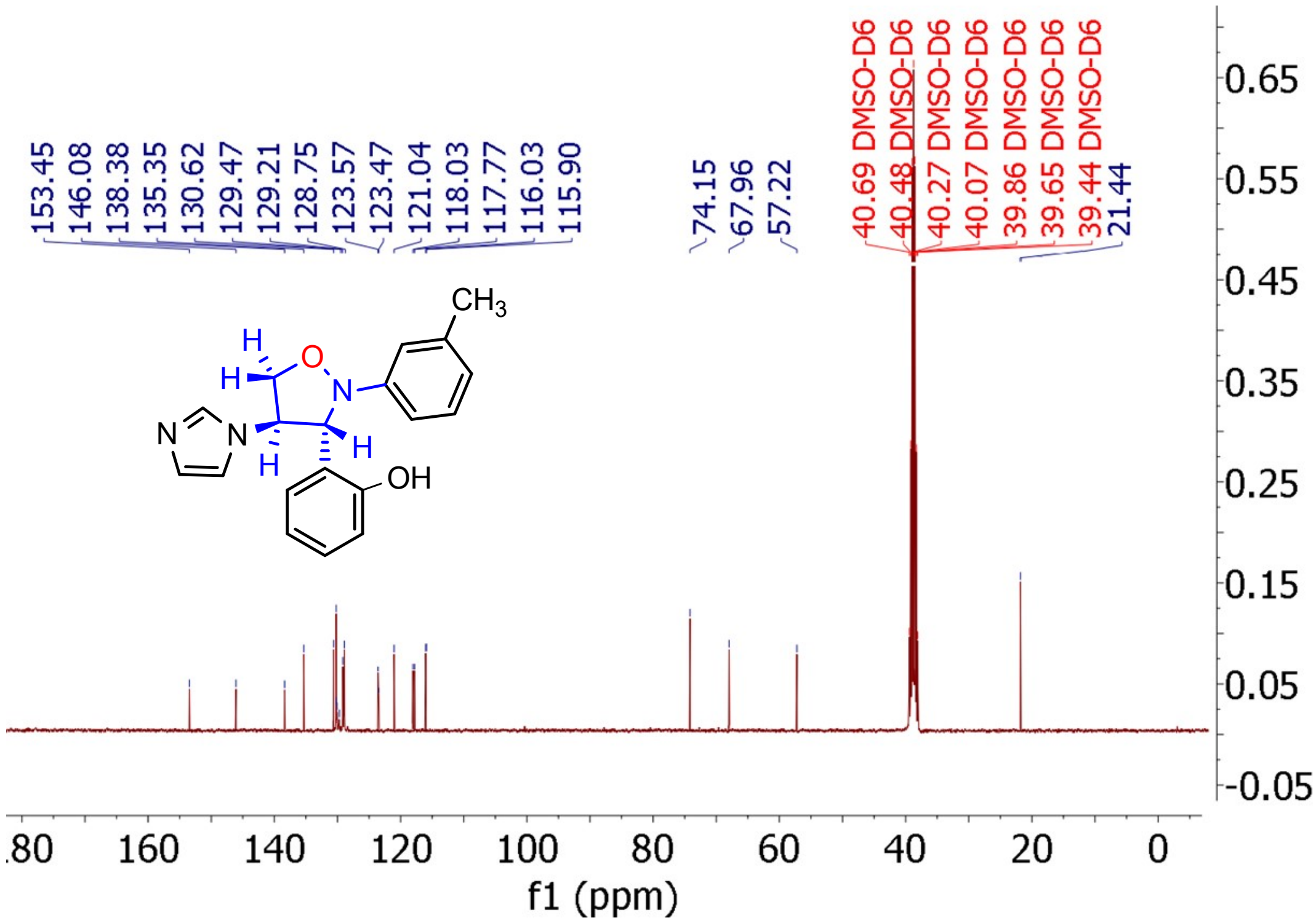

Figure 38: ^{13}C-NMR for isoxazolidine (3g)

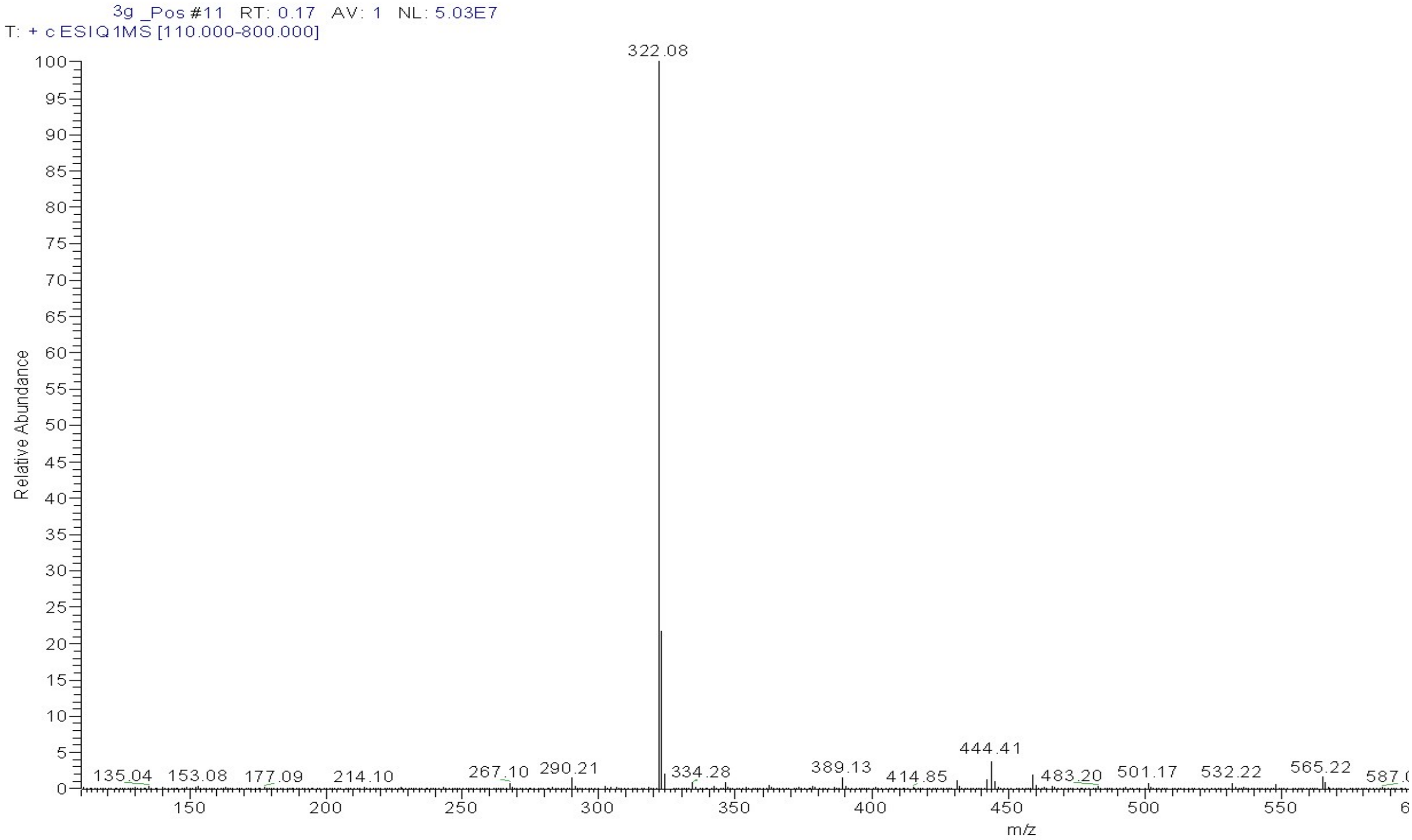

Figure 39: LC-MS for isoxazolidine (3g)

#	Time	Area	Height	Width	Area%	Symmetry
1	13.016	4418.8	902.4	0.0816	98.798	0.642

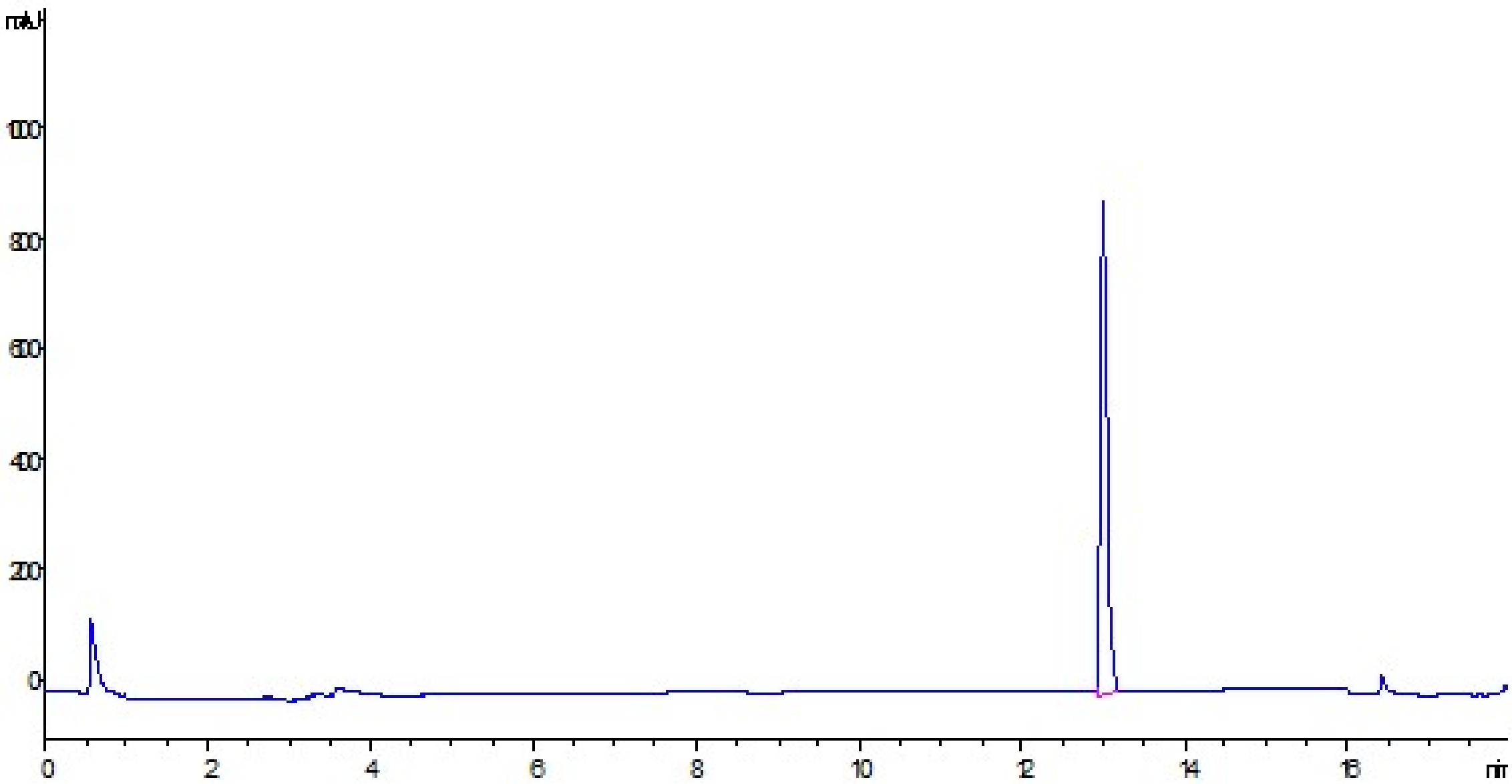

Figure 40: HPLC purity analysis of isoxazolidine (3g)

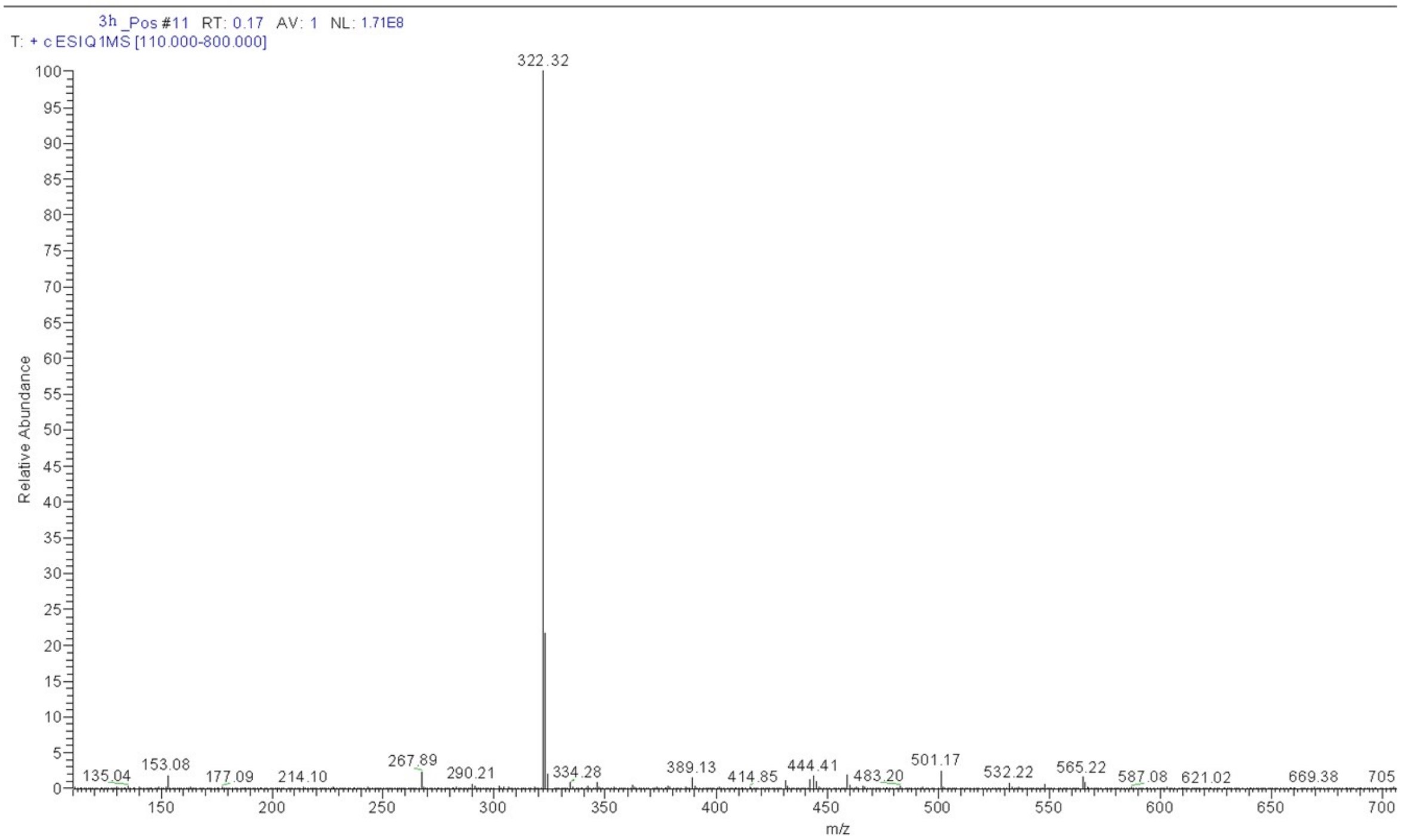

Figure 41: LC-MS for isoxazolidine (3h)

#	Time	Area	Height	Width	Area%	Symmetry
1	11.328	26.9	227.2	0.078	5.000	0.659
2	15.263	1764.5	1387.2	0.0926	95.000	0.631

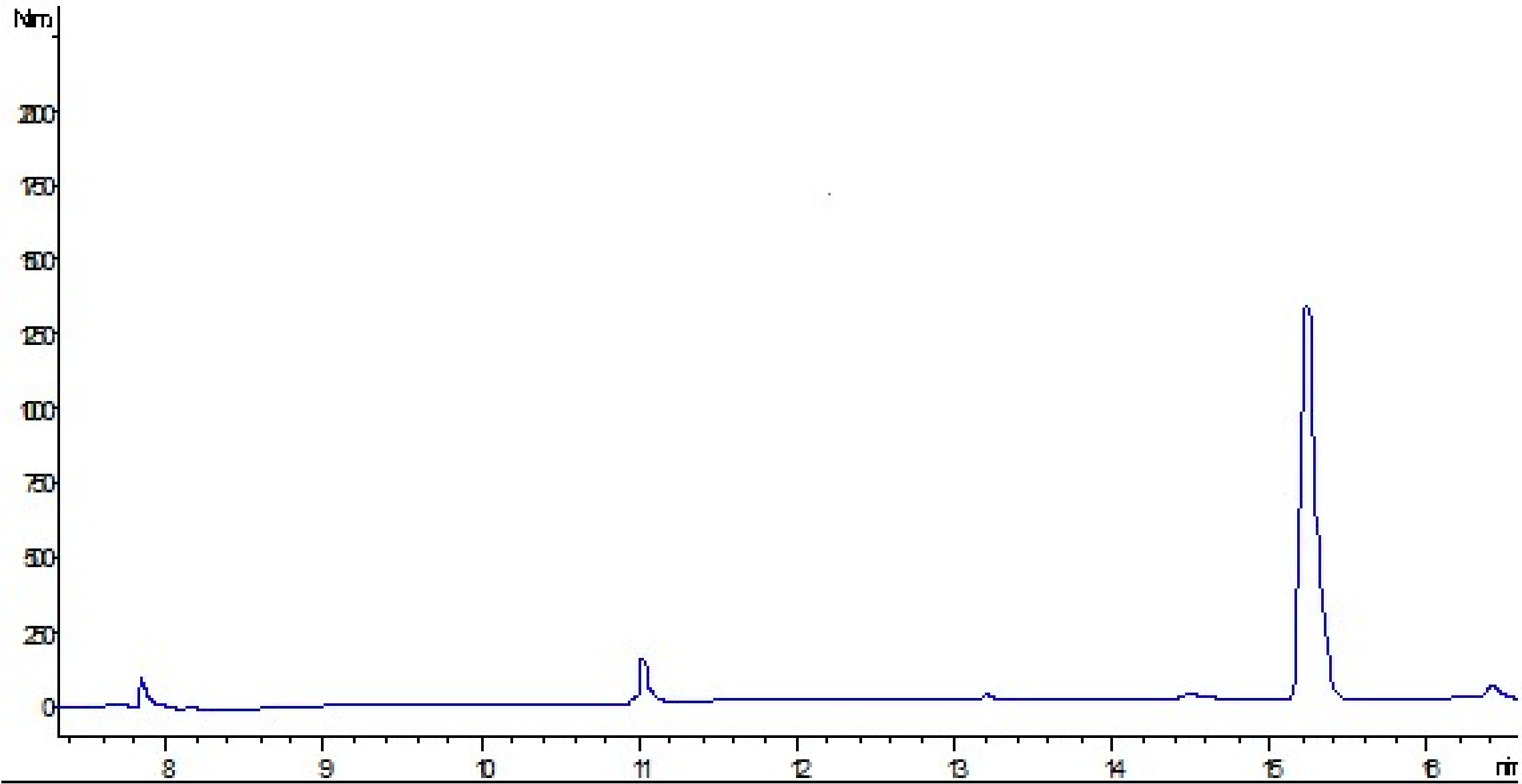

Figure 42: HPLC purity analysis of isoxazolidine (3h)

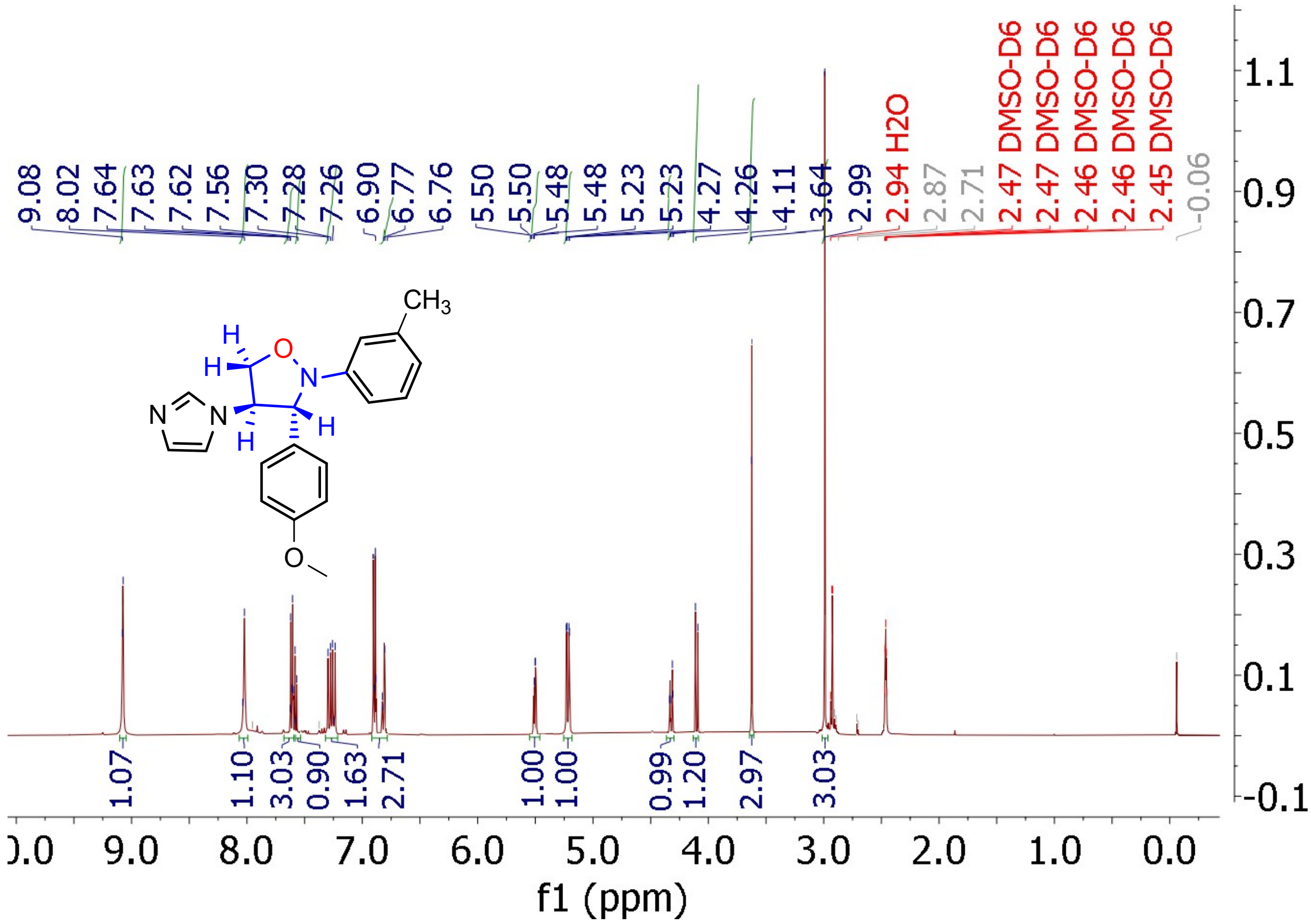

Figure 43: ^{1}H-NMR of isoxazolidine (3i)

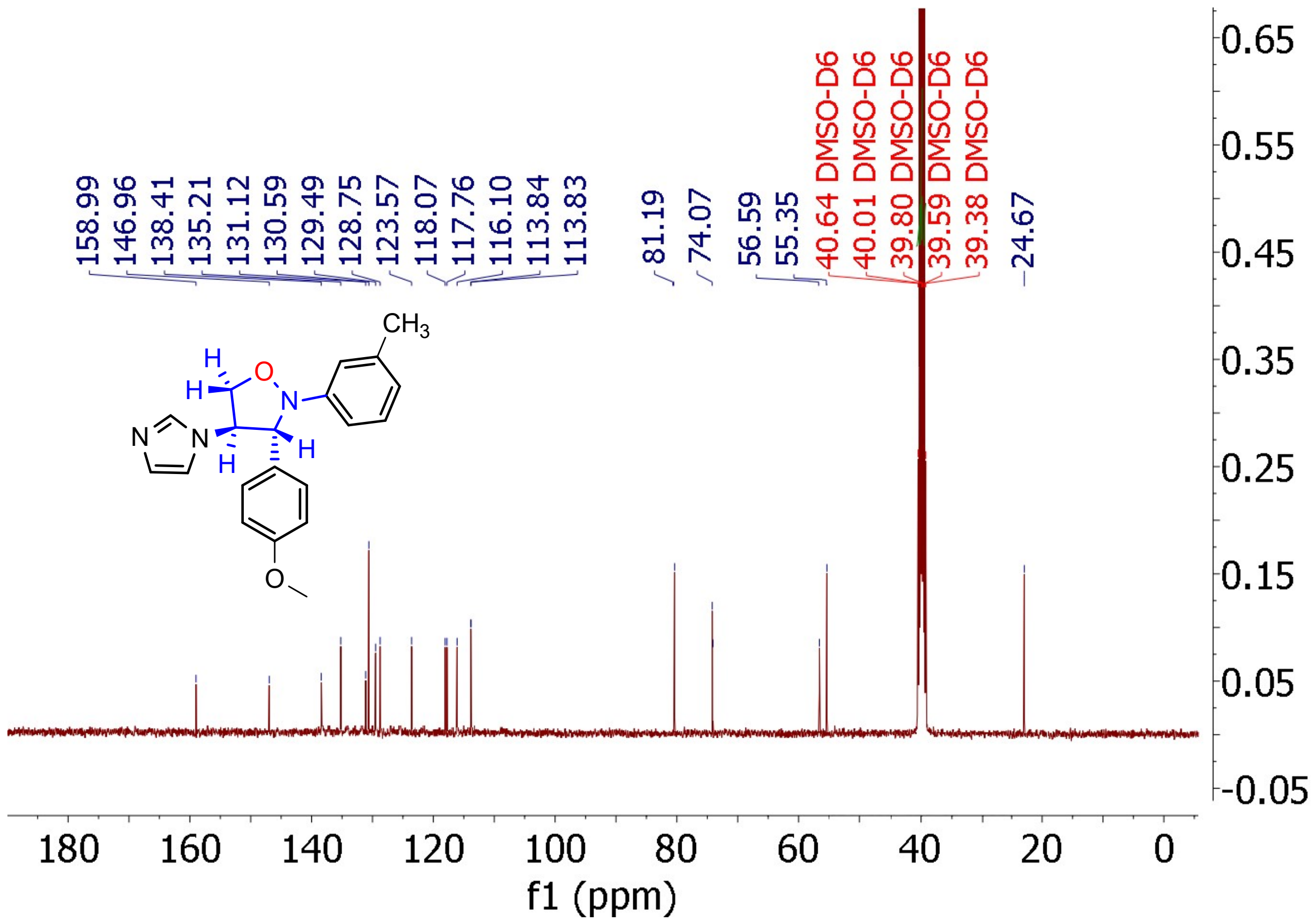

Figure 44: ^{13}C-NMR for isoxazolidine (3i)

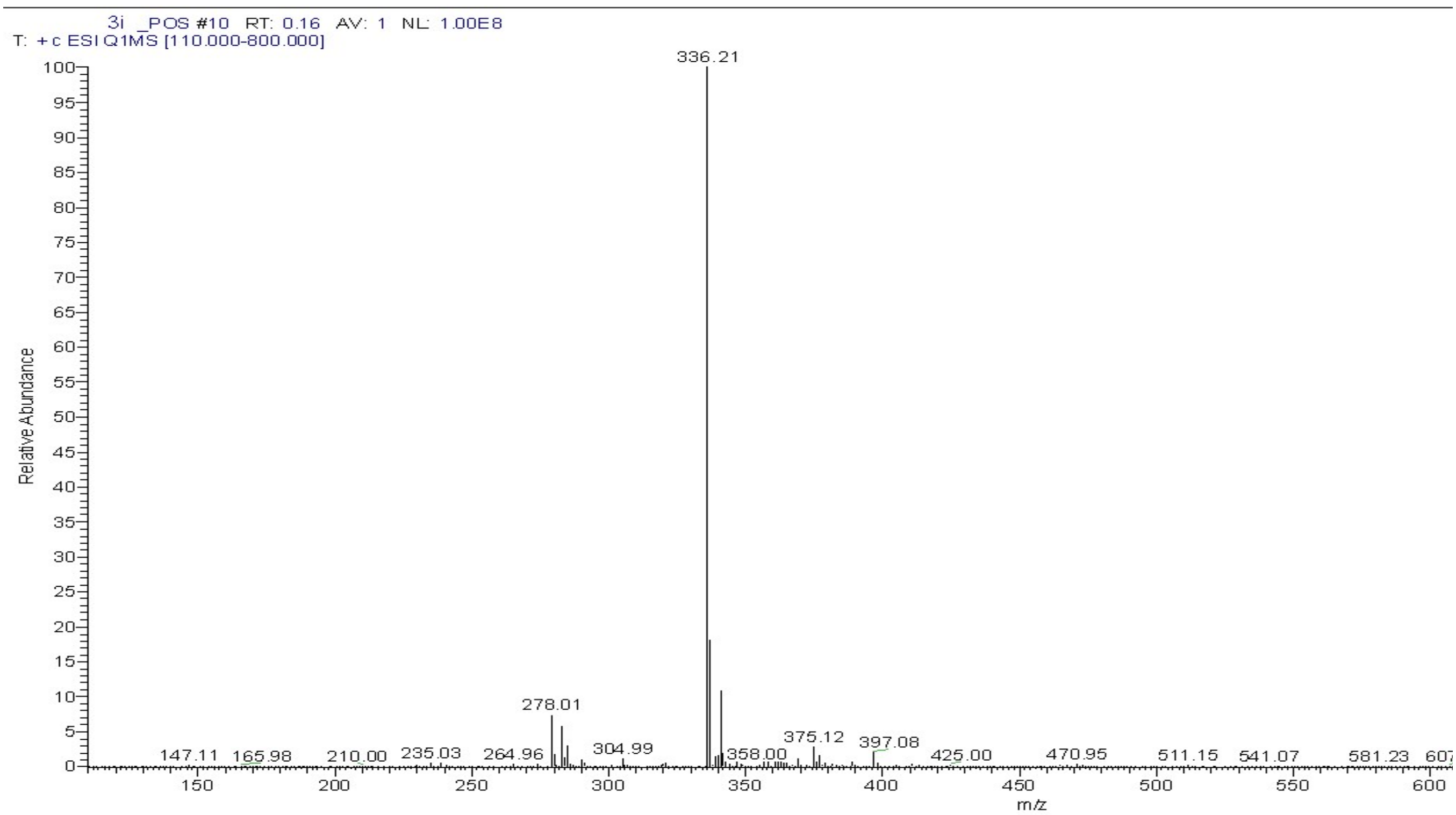

Figure 45: LC-MS for isoxazolidine (3i)

#	Time	Area	Height	Width	Area%	Symmetry
1	11.33	11683.1	2812.6	0.087	97.553	0.637
2	12.035	168.2	32.8	0.0854	1.118	0.673
3	15.273	200	39.4	0.0846	1.329	0.536

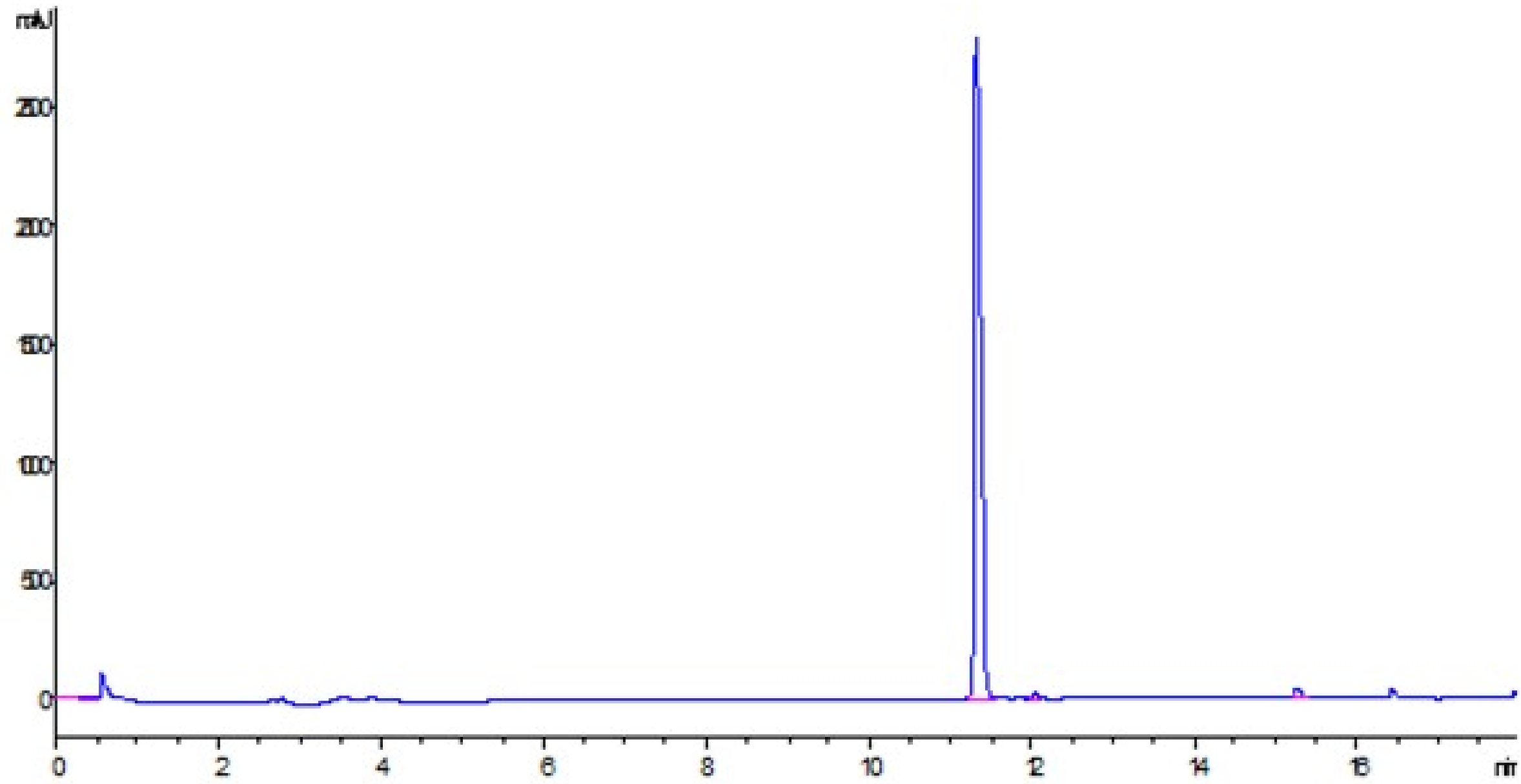

Figure 46: HPLC purity analysis of isoxazolidine (3i)

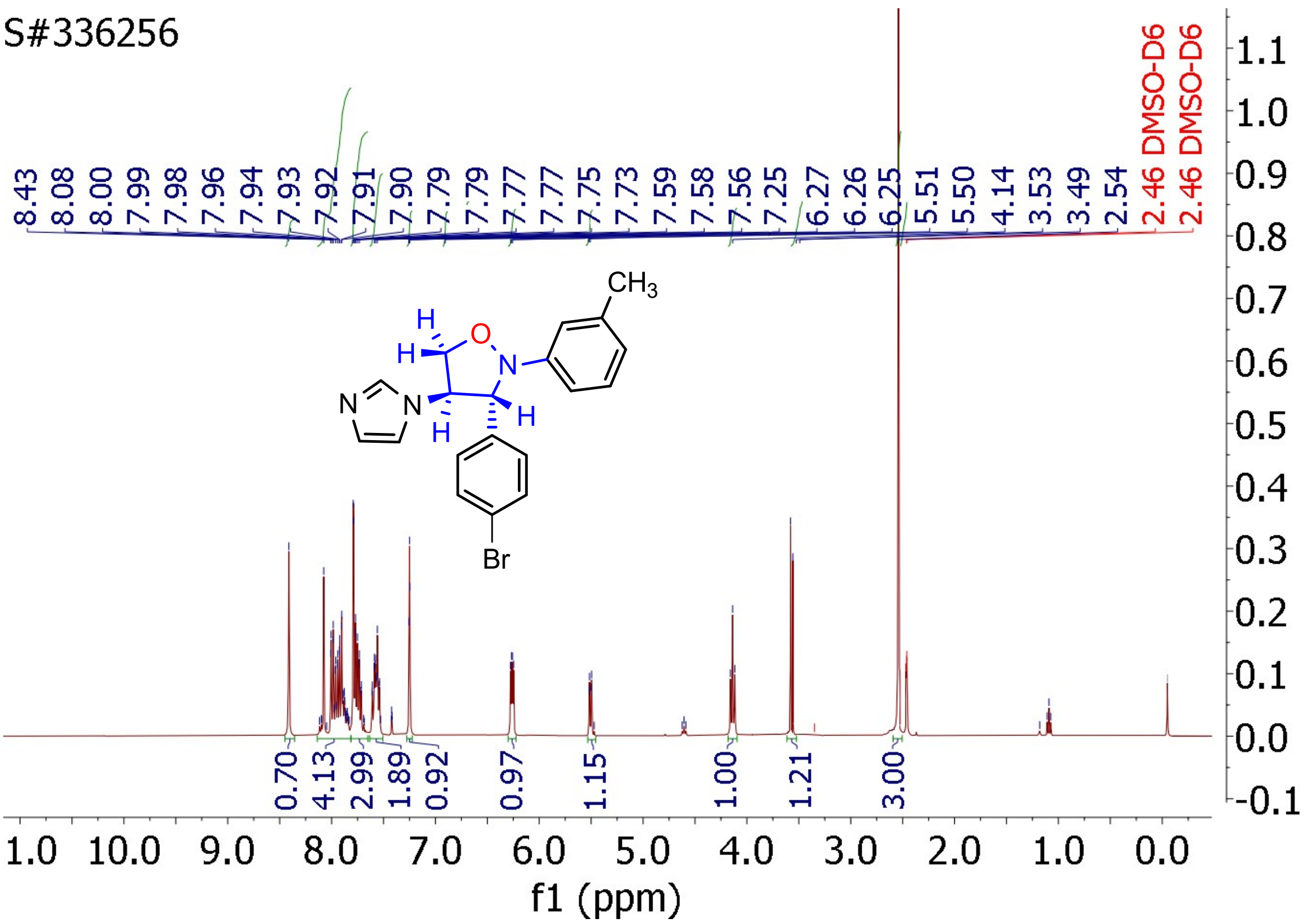

Figure 47: ^{1}H-NMR of isoxazolidine (3j)

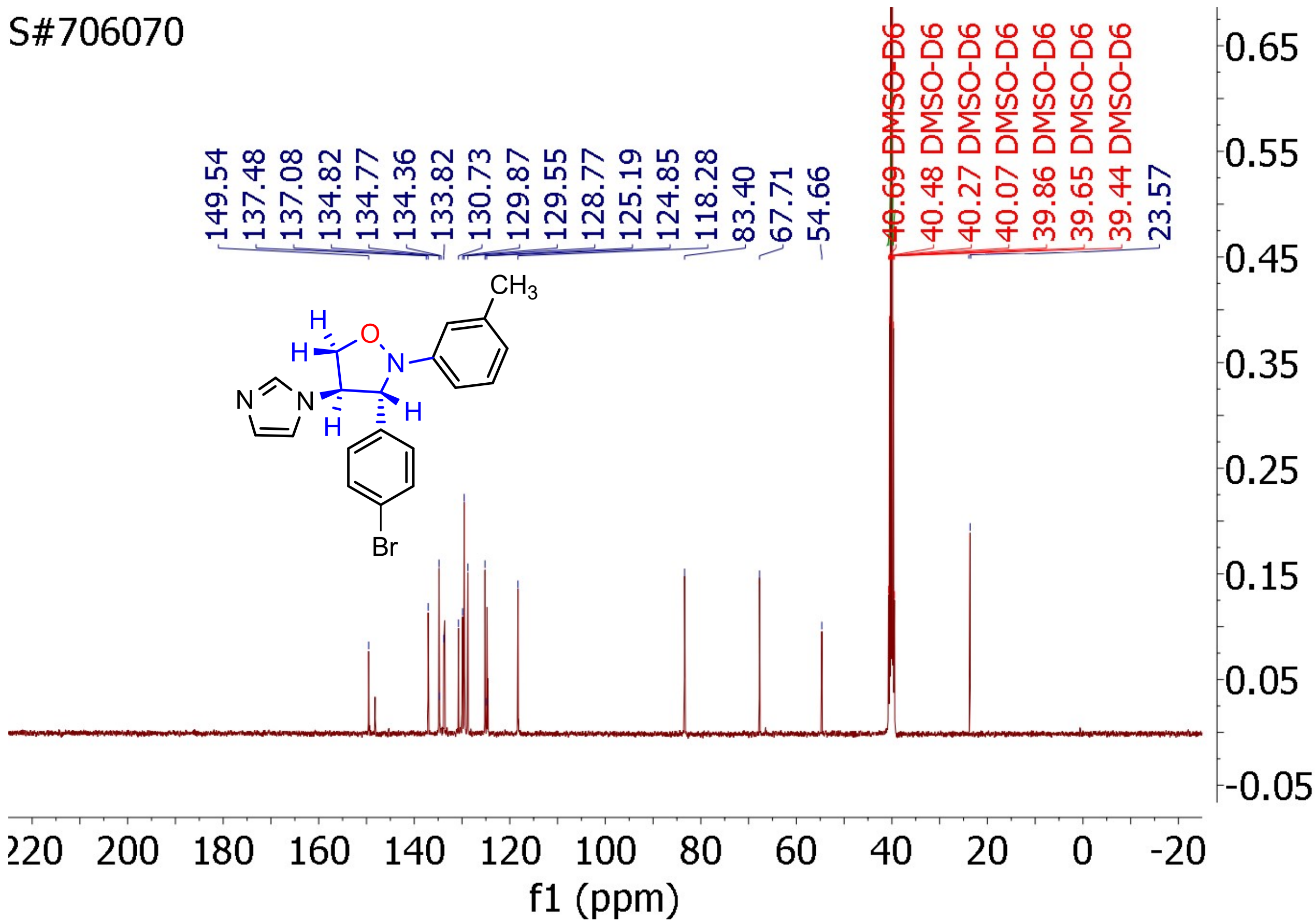

Figure 48: ^{13}C-NMR for isoxazolidine (3j)

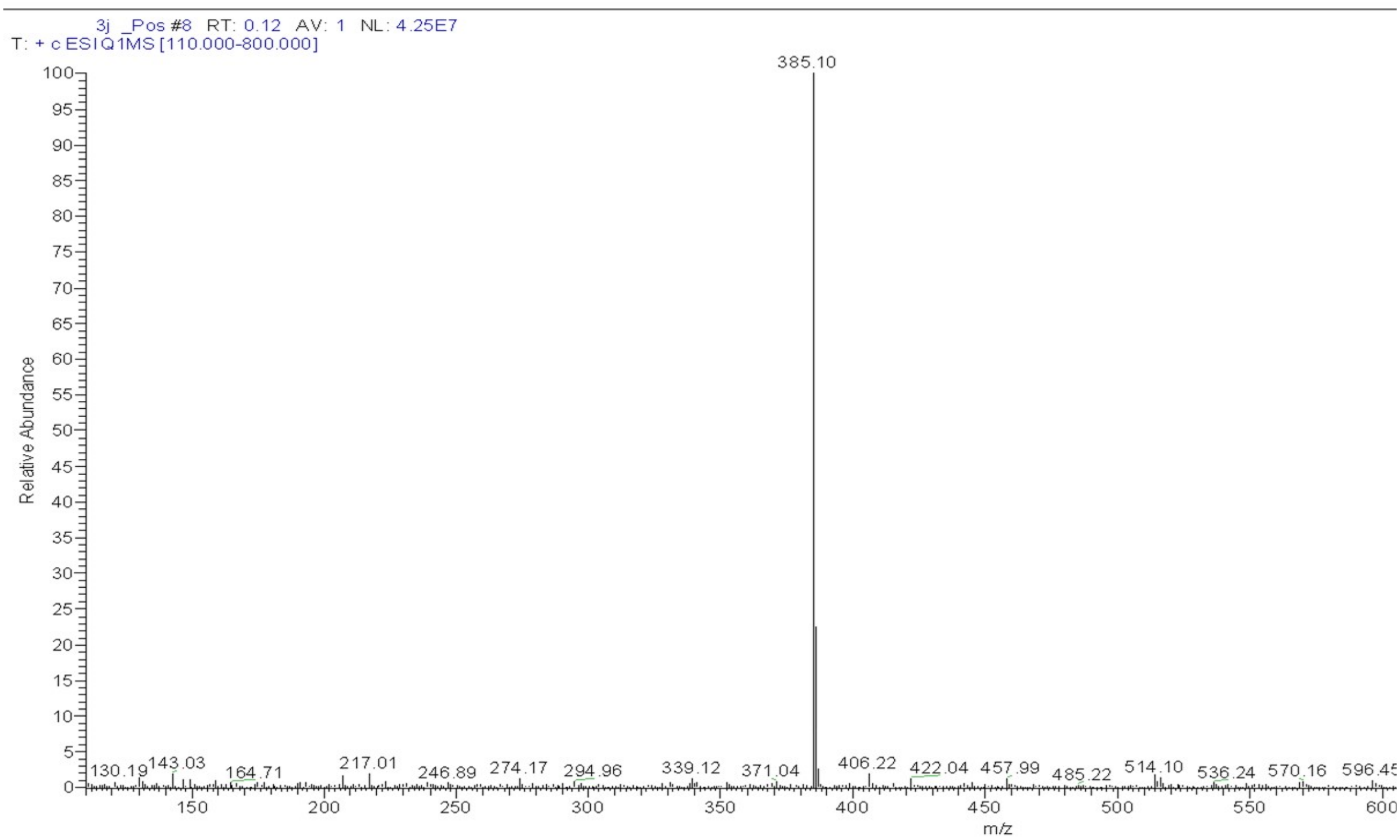

Figure 49: LC-MS for isoxazolidine (3j)

#	Time	Area	Height	Width	Area%	Symmetry
1	12.311	5758.1	1197.5	0.0801	98.130	0.666
2	15.257	4170.8	774.4	0.0816	1.870	0.642

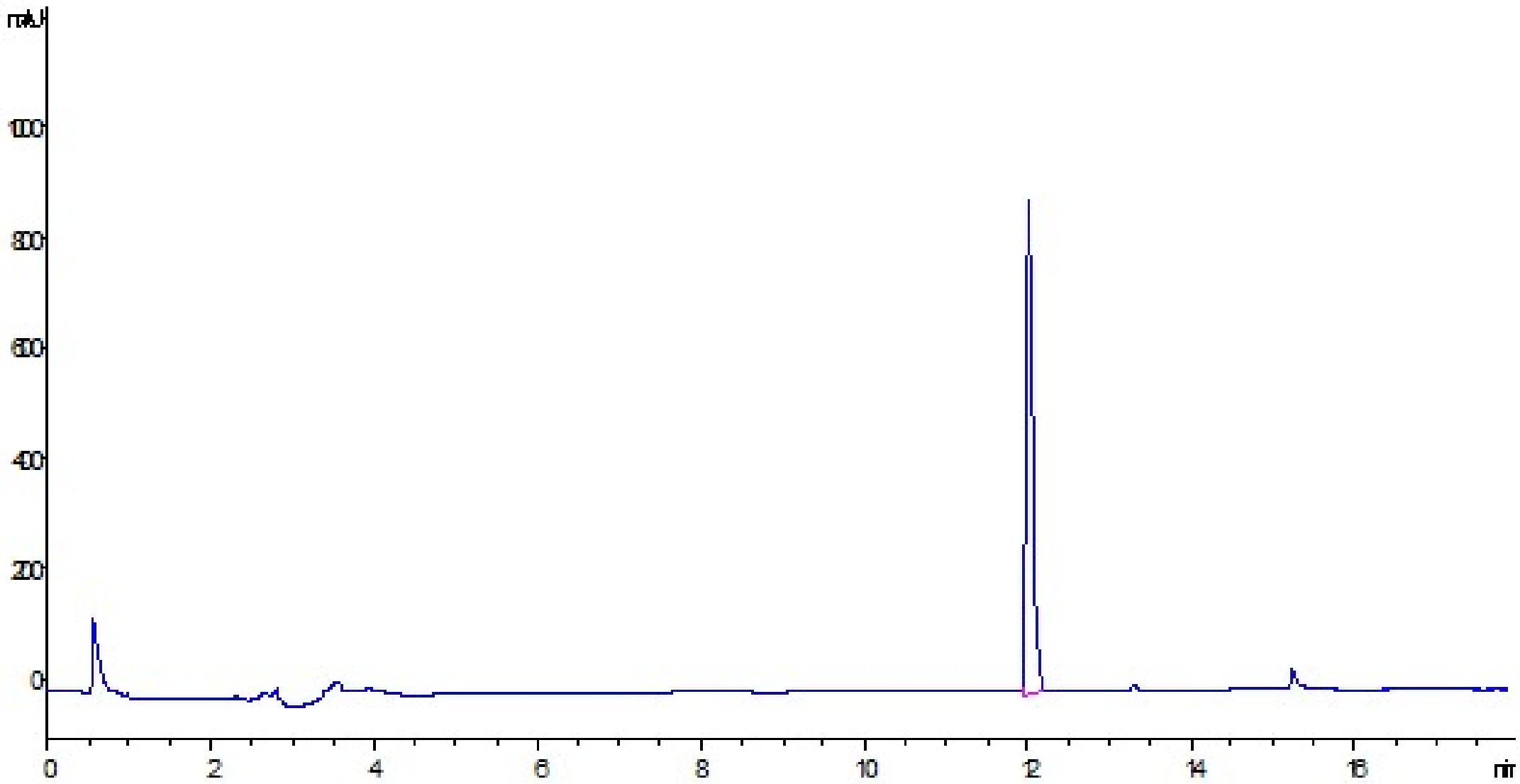

Figure 50: HPLC purity analysis of isoxazolidine (3j)

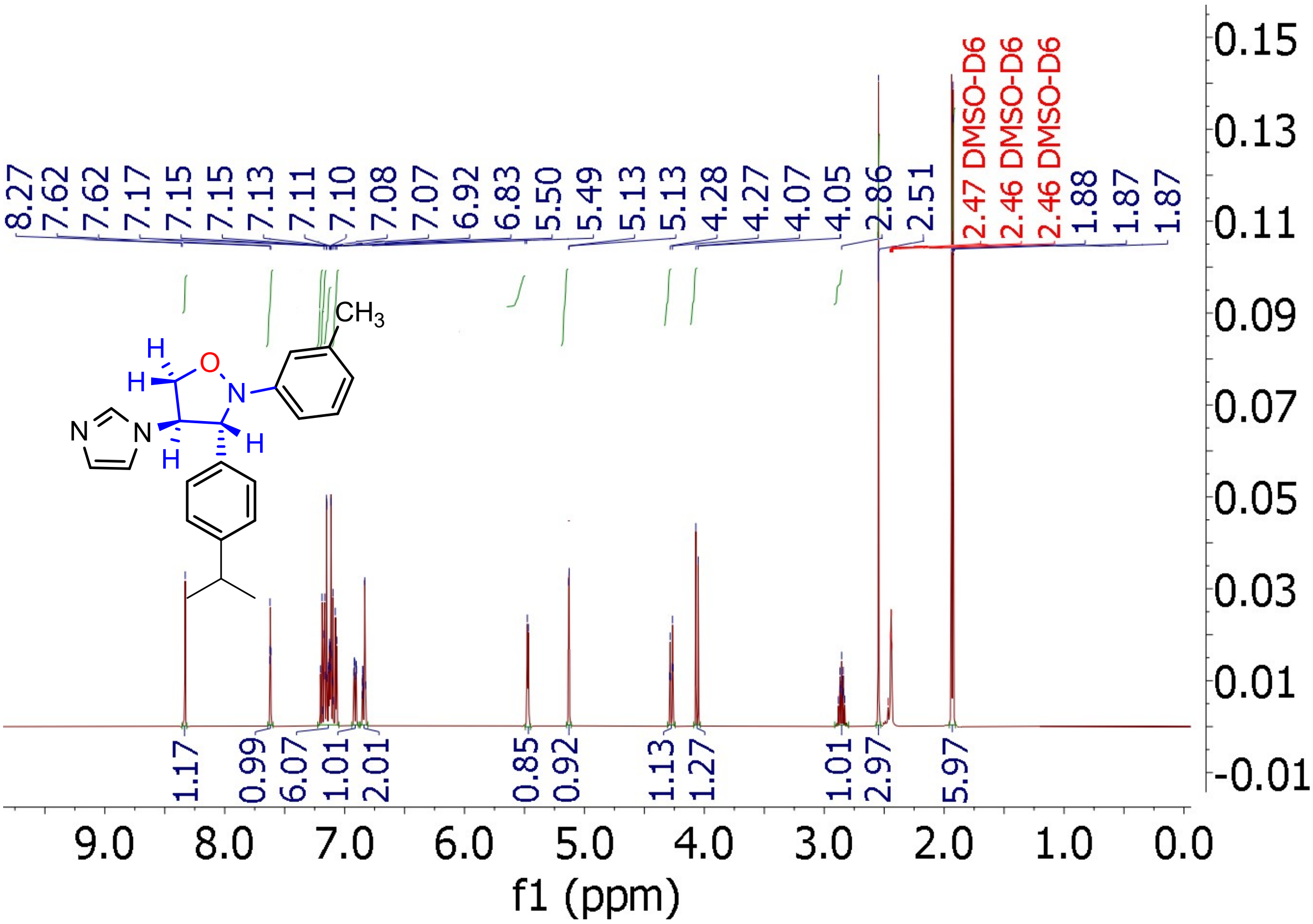

Figure 51: ^{1}H-NMR of isoxazolidine (3k)

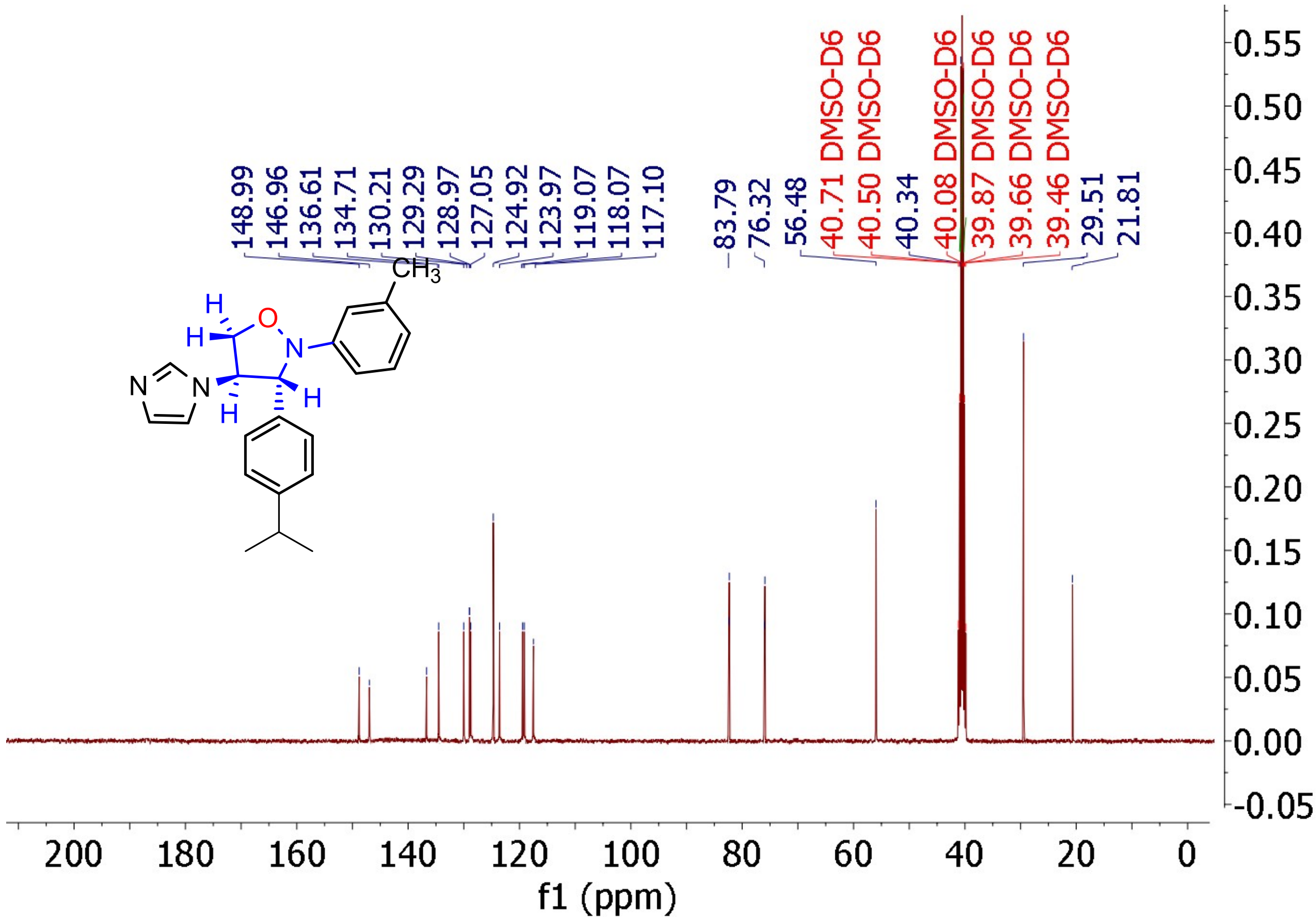

Figure 52: ^{13}C-NMR for isoxazolidine (3k)

#	Time	Area	Height	Width	Area%	Symmetry
1	11.324	2443.2	517.9	0.0786	96.251	0.67

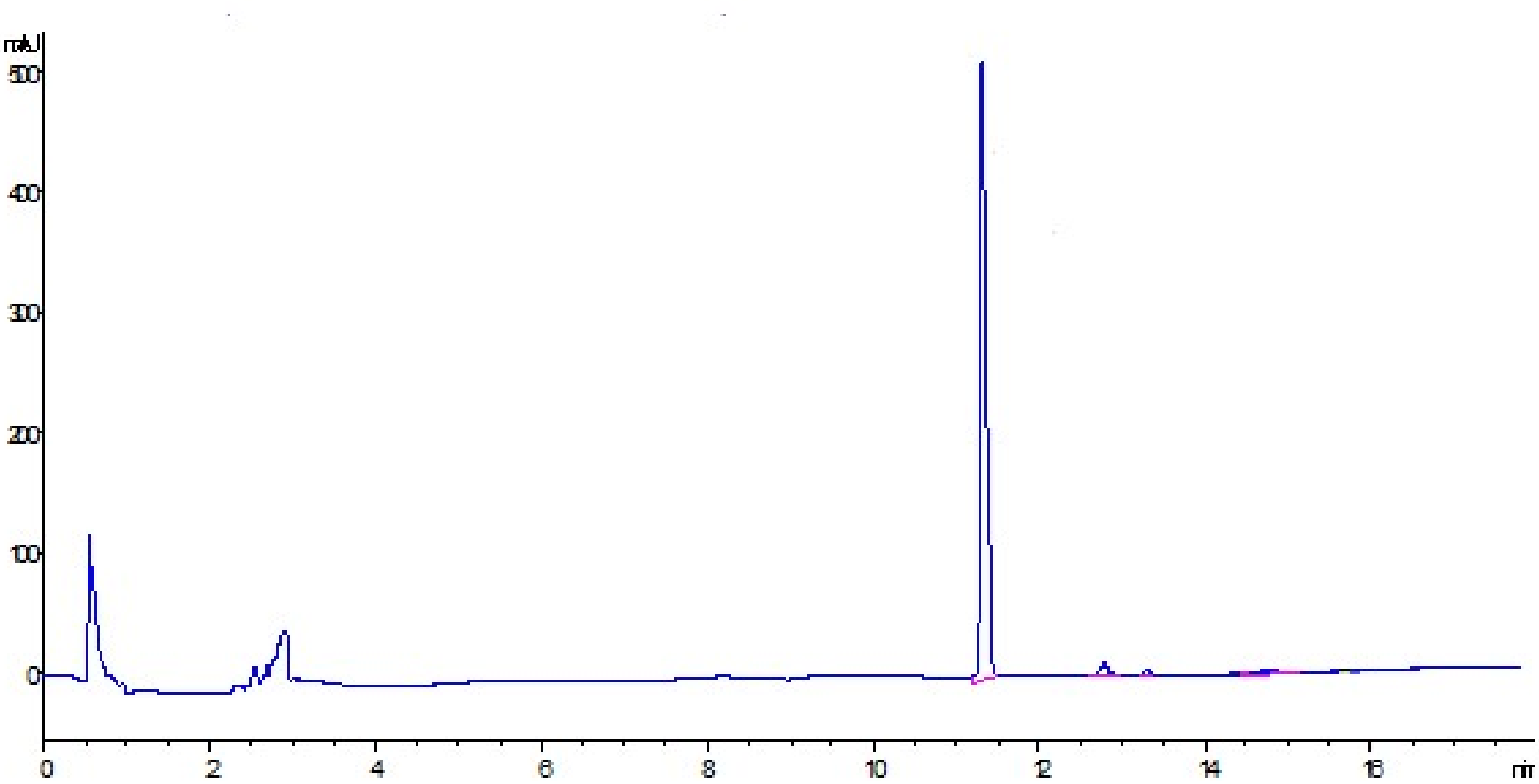

Figure 53: HPLC purity analysis of isoxazolidine (3k)

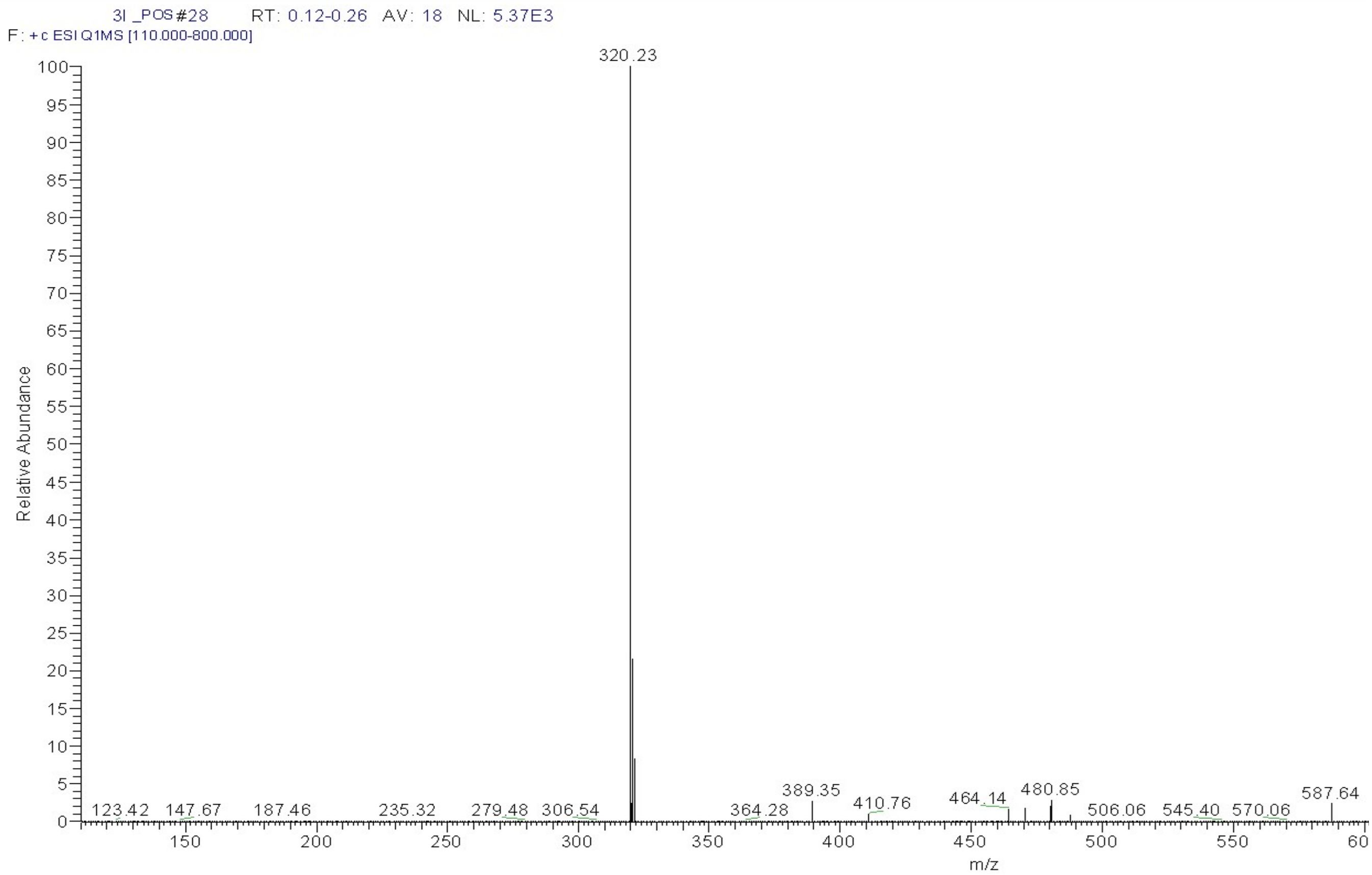

Figure 54: LC-MS for isoxazolidine (3l)

#	Time	Area	Height	Width	Area%	Symmetry
1	11.3061	5723.6	1072	0.0827	97.964	0.842
2	12.072	73.3	17.3	0.0697	1.342	0.964
3	15.42	15	9.8	0.0717	0.722	0.849

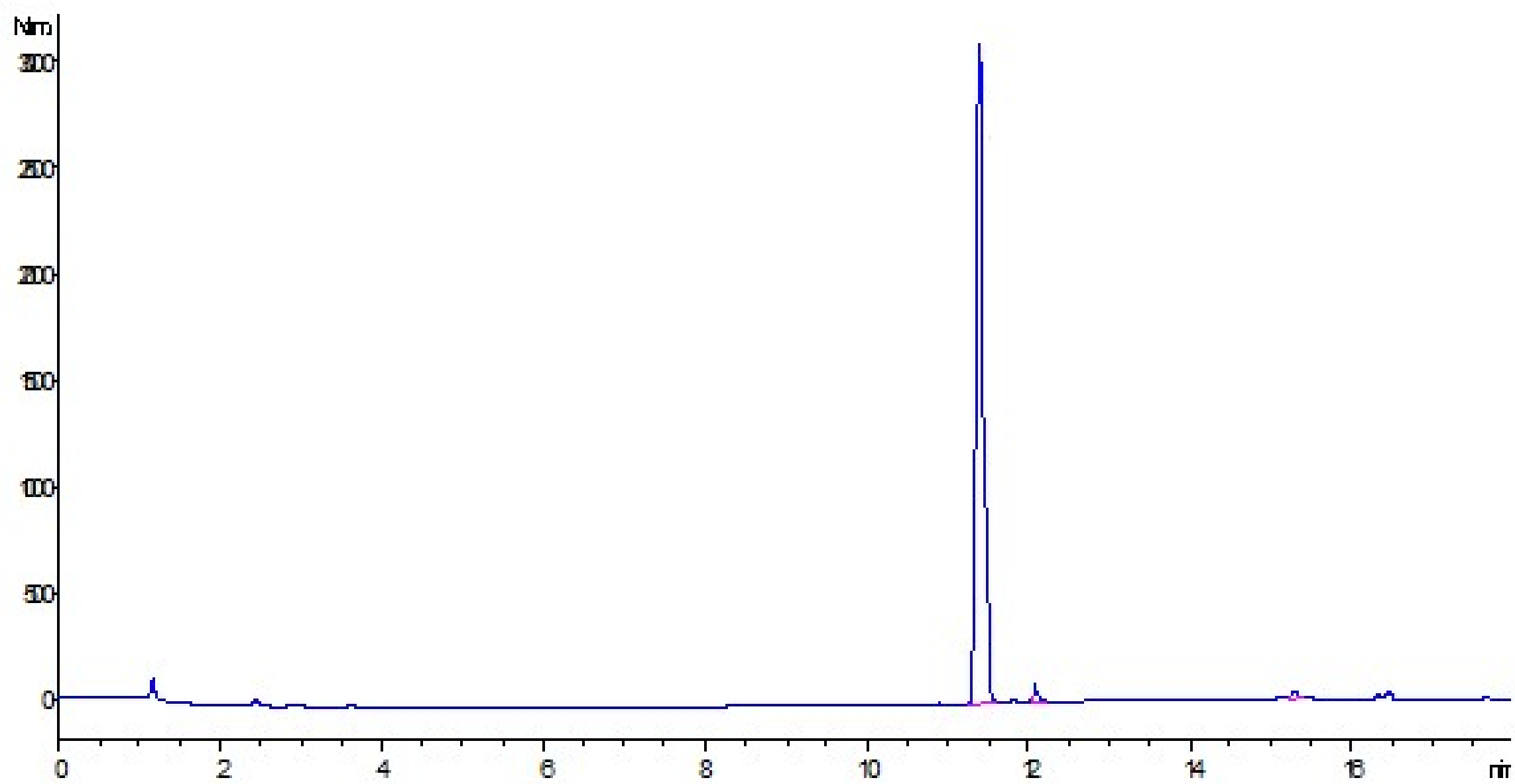

Figure 55: HPLC purity analysis of isoxazolidine (3l)
